# Decoration of Ag nanoparticles on CoMoO_4_ rods for efficient electrochemical reduction of CO_2_

**DOI:** 10.1038/s41598-024-51680-w

**Published:** 2024-01-16

**Authors:** Schindra Kumar Ray, Rabin Dahal, Moses D. Ashie, Bishnu Prasad Bastakoti

**Affiliations:** Department of Chemistry, North Carolina A and T State University, 1601 E Market St, Greensboro, NC 27411 USA

**Keywords:** Chemistry, Catalysis, Inorganic chemistry, Materials chemistry

## Abstract

Hydrothermal and photoreduction/deposition methods were used to fabricate Ag nanoparticles (NPs) decorated CoMoO_4_ rods. Improvement of charge transfer and transportation of ions by making heterostructure was proved by cyclic voltammetry and electrochemical impedance spectroscopy measurements. Linear sweep voltammetry results revealed a fivefold enhancement of current density by fabricating heterostructure. The lowest Tafel slope (112 mV/dec) for heterostructure compared with CoMoO_4_ (273 mV/dec) suggested the improvement of electrocatalytic performance. The electrochemical CO_2_ reduction reaction was performed on an H-type cell. The CoMoO_4_ electrocatalyst possessed the Faraday efficiencies (FEs) of CO and CH_4_ up to 56.80% and 19.80%, respectively at  − 1.3 V versus RHE. In addition, Ag NPs decorated CoMoO_4_ electrocatalyst showed FEs for CO, CH_4_, and C_2_H_6_ were 35.30%, 11.40%, and 44.20%, respectively, at the same potential. It is found that CO_2_ reduction products shifted from CO/CH_4_ to C_2_H_6_ when the Ag NPs deposited on the CoMoO_4_ electrocatalyst. In addition, it demonstrated excellent electrocatalytic stability after a prolonged 25 h amperometric test at  − 1.3 V versus RHE. It can be attributed to a synergistic effect between the Ag NPs and CoMoO_4_ rods. This study highlights the cooperation between Ag NPs on CoMoO_4_ components and provides new insight into the design of heterostructure as an efficient, stable catalyst towards electrocatalytic reduction of CO_2_ to CO, CH_4_, and C_2_H_6_ products.

## Introduction

The dramatic increase in CO_2_ concentration in the atmosphere leads to notable environmental issues^1^. So, the conversion of CO_2_ into valuable chemical products plays a vital role to minimize the greenhouse effect and maintain the global carbon balance that has recently attracted significant attention to researchers^2–6^. Among different CO_2_ conversion strategies, the electrochemical CO_2_ reduction reaction (CO_2_RR) reveals a perfect approach for usable chemicals and fuels productions^7,8^. The combination of carbon-based electro-fuel production from CO_2_RR with renewable energy exhibits the great hope of global carbon neutrality. However, slow reaction kinetics lowers the performance of the electrocatalyst for CO_2_RR^9,10^. Therefore, fabrication of a novel electrocatalyst is urgent to solve the problems related with sluggish kinetics and high overpotential.

Ag has been regarded as a promising CO_2_RR catalyst due to its relatively low overpotential, high selectivity, tendency of lowering the CO_2_ reduction reaction barrier, better solubility of CO_2_, electron transfer, improvement of local catalytic environment, suppression of hydrogen evolution reaction (HER), and appropriate (neither too strong nor too weak) binding strength with products^11–13^. It is considered as a benchmark electrocatalysts for selective conversion of CO_2_ to CO with good Faradic efficiencies. Despite these advantages, pure Ag NPs suffer from maintaining the size/structure, high-cost, and stability due to their high level of surface energy^14^. So, the construction of heterostructure interface between pure metal and low-cost metal oxide materials is a perfect choice because it induces synergistic effects to promote the stability/selectivity/electrocatalytic efficiency, reduce the overpotential, and conversion of CO_2_ to CO, C1 and multi-carbon products^10,14^.

Nowadays, Ag NPs have been coupled with several metal oxides such as TiO_2_, CuOx, Cr_2_O_3_, MnO_2_, SnO_2_, etc. for CO_2_RR. However, these oxide materials suffer from low conductivity and insufficient catalytic performances^15–19^. Also, these heterostructures cannot produce C2 (C_2_H_6_) during electrocatalytic CO_2_RR. To solve these issues, selecting cobalt molybdate (CoMoO_4_) is the best metal oxide material because Co-based oxides provide excellent catalytic activities and Mo-based materials demonstrate outstanding electrical conductivity^20^. Also, the synergistic integration of Co and Mo improves the electrocatalytic properties of CoMoO_4_. In addition, it has several advantages such as stable crystal structure, redox performance, favorable physical/chemical properties, enhancement of electrolyte–electrode surface area, excellent mechanical stability, ionic conductivity, generation of active sites, small overpotential, environmentally friendly, inexpensive, and abundant resources^21–25^. The multiple oxidation states of cobalt involved at the intermediate state for CO_2_RR^26^. Furthermore, rods like structure or nanorods can contribute a higher contact area along with great electron pathways than other morphologies^21^. Although lots of paper has been published for Ag NPs towards CO_2_RR, Ag NPs decorated CoMoO_4_ rod heterostructure has rarely been reported for CO_2_ reduction to CO, C1 and multi-carbon compounds.

In this study, Ag/CoMoO_4_ heterostructure was synthesized by hydrothermal and photoreduction/deposition methods. The hydrothermal method revealed several advantages as compared to others, such as low cost, mass efficiency, high product purity, mild preparation conditions, and simple equipment^20,24,27–31^. In addition, photoreduction process is simple and inexpensive and can be operated at room temperature^32^. The formation of heterostructure is well characterized by ﻿X-ray diffractometry (XRD), field emission scanning electron microscopy (FESEM), transmission electron microscopy (TEM), elemental mapping, ﻿X-ray photoelectron spectroscopy (XPS), Raman spectroscopy, Fourier-transform infrared spectroscopy (FTIR), and Inductively coupled plasma optical emission spectroscopy (ICP-OES). The electrochemical measurements (cyclic voltammetry, electrochemical impedance spectroscopy, linear sweep voltammetry, and chronoamperometry) of catalysts were performed in an H-type cell for electrochemical CO_2_RR. Tafel plots were analyzed. The gaseous products were detected by gas chromatography (GC). ﻿The Faradic efficiencies (FEs) of CO_2_RR was calculated, and possible mechanisms were proposed.

## Experimental section

### Materials

All chemicals consist of analytical grade. These were used without any further purification. Copper foil (CF) with 0.1 mm thickness was purchased from Merck, Germany. Molybdic acid (H_2_MoO_4_) and cobalt nitrate hexahydrate [Co(NO_3_)_2_.6H_2_O], potassium bicarbonate (KHCO_3_), and aqueous ammonia (aq. NH_3_) were used for the synthesis of samples and obtained from the Sigma-Aldrich. Silver nitrate (AgNO_3_) was purchased from Fisher chemical, Belgium.

### Synthesis of CoMoO_4_ and Ag/CoMoO_4_

CoMoO_4_ was synthesized from hydrothermal process. In this synthesis technique, 2 × 10^–2^ mol of H_2_MoO_4_ was placed in 40 mL of water. In addition, 2 × 10^–2^ mol of Co(NO_3_)_2_·6H_2_O was dissolved in 40 mL of water. These were magnetically stirred until clear solution was obtained. Then, the prepared solutions were mixed dropwise by using pipette under magnetic stirring and precipitation was occurred. The pH of the solution was adjusted at pH 7 by using aqueous ammonia (NH_3_). It was magnetically stirred for 4 h. After that, the suspension solution was transferred into a 100 mL Teflon-lined stainless autoclave and kept at 200 °C for 4 h. After completion of hydrothermal treatment, the solution was centrifuged and washed with water and ethanol multiple times. Subsequently, it was dried in a vacuum oven at 70 °C for 8 h. The powder was obtained and calcined at 400 °C for 5 h. At last, the powder sample (CoMoO_4_) was grounded with the help of mortar and piston.

Ag/CoMoO_4_ was fabricated by hydrothermal followed by photoreduction/deposition techniques. According to this technique, hydrothermal synthesized 1 g of CoMoO_4_ powder was taken and placed in 100 mL beaker. 80 mL ethyl alcohol (C_2_H_5_OH) was put in a beaker and magnetically stirred for 2 h. 5 wt% of Ag (source: AgNO_3_) was placed in a beaker and magnetically stirred for 4 h under UV light irradiation. After this step, it was centrifuged and washed with water/C_2_H_5_OH several times. The obtained sample was dried in a vacuum oven for 70 °C for 4 h. Finally, it was grounded. The schematic illustration of material synthesis was presented in Fig. S1.

### Material characterization

The crystal phase was determined using a powder X-ray diffractometer (Rigaku, Miniflex 600) with Cu Kα radiation (2*θ*: 20 to 80°, continuous rate: 1°/minute, and step: 0.02). The morphologies of samples were investigated by field emission scanning electron microscopy (FESEM, JEOL, JSM-IT800). Transmission electron microscopy (TEM), high-resolution transmission electron microscopy (HRTEM), and selected area diffraction patterns (SAED), EDS elemental mapping images were obtained by JEOL 1230. The X-ray photoelectron spectroscopy (XPS) analysis of the samples was performed using Thermo Scientific ESCALAB™ XI (Al Kα and 200 eV). The Raman spectra of samples were measured on Horiba Raman confocal microscope. Fourier-transform infrared spectroscopy (FTIR) of samples were measured on IRTracer-100 (Shimadzu). Inductively coupled plasma optical emission spectroscopy (ICP-OES) was used to find the leaching of Ag NPs after performance of electrochemical CO_2_ RR by samples. It is also used to find out the metal ions in the samples. The detail explanation was provided in supporting information (Figs. S2, S3, and S4). Zeta potential of powder samples was measured by Zetasizer Nano ZS (Malvern Instruments, Malvern, UK). The powder was dispersed in 70% ethanol (15 mL) and placed in an ultrasonic bath for 1 h. The zeta potential was measured after diluting the samples with distilled water.

### Electrochemical characterizations

All the electrochemical measurements were carried out on a CH Instruments with a typical three-electrode system in 0.5 M KHCO_3_ electrolyte solution at room temperature, a platinum electrode (counter electrode), Ag/AgCl electrode (reference electrode), and working electrodes (CoMoO_4_ and Ag/CoMoO_4_). For the synthesis of working electrodes, 0.5 mL of C_2_H_5_OH, 50 μL nafion, and 4 mg of powder sample were dispersed via ultrasonic processing. As a substrate, CF (2 cm × 2 cm) was washed with water and ethanol for 60 min under ultrasonication and dried at 70 °C for 4 h in a vacuum oven. The well-dispersed ink was placed in CF via controllable drop casting. The available working area in the electrode was 1 cm^2^. Then, it was dried in an oven at 70 °C for 4 h.

Cyclic voltammetry (CV) with scan rate 20 to 150 mV/s of samples was measured. In addition, the electrical conductivity of the samples was performed through the electrochemical impedance spectroscopy (EIS) that consists of 0.1 Hz to 100,000 Hz. The linear sweep voltammetry (LSV) of the samples was observed from 0 to  − 0.6 V versus RHE at scan rate 10 mV/s. Tafel plots were obtained at potential  − 1 to 1 V. The reversible hydrogen electrode (RHE) was calculated using following equation: E_RHE_ = E_Ag/AgCl_ + 0.197 + 0.059 pH (0.5 M KHCO_3_ ~ 8.52), where E_Ag/AgCl_ represents potential against the reference electrode and 0.197 V indicates the standard potential of Ag/AgCl at 25 °C^33^.

The electrochemical CO_2_RR was performed on H-type cell. A 50 mL electrochemical cell was used that consists of 35 mL electrolyte. The anodic and cathodic compartments were separated by Nafion 117 membrane. This membrane was washed with acid/water before using in H-type cell. A stream of pure (99.999%) CO_2_ gas was continuously passed in the cell for saturation of electrolyte for 60 min at 5 sccm using mass flow controller (MC-100SCCM-D, Alicat Scientific). The gas outlet of H -type cell (cathodic compartment) was connected to a gas chromatograph (SRI 8610C). The GC is equipped with flame ionization detector (FID) for determining hydrocarbon and CO products during electrochemical CO_2_RR. The carrier gas for FID is helium GC was calibrated using standard gas mixtures (ARC3) under 1 atm and 298 K. Amperometry *i-t* experiments were conducted at fixed potentials  − 1.3 V versus RHE. The first injection of gas in GC was performed 400 s after the start of CO_2_ reduction. The different concentration of gases in ppm was noted and Faradic efficiencies were calculated on  − 1.3 V versus RHE. (Supplementary information). In addition, electrocatalytic stability was evaluated for 25 h under similar applied potential. For comparison of efficiency of electrocatalyst, electrocatalytic performance of the copper foil was carried out.

## Results and discussion

### Characterization of synthetic materials

**Figure 1:**
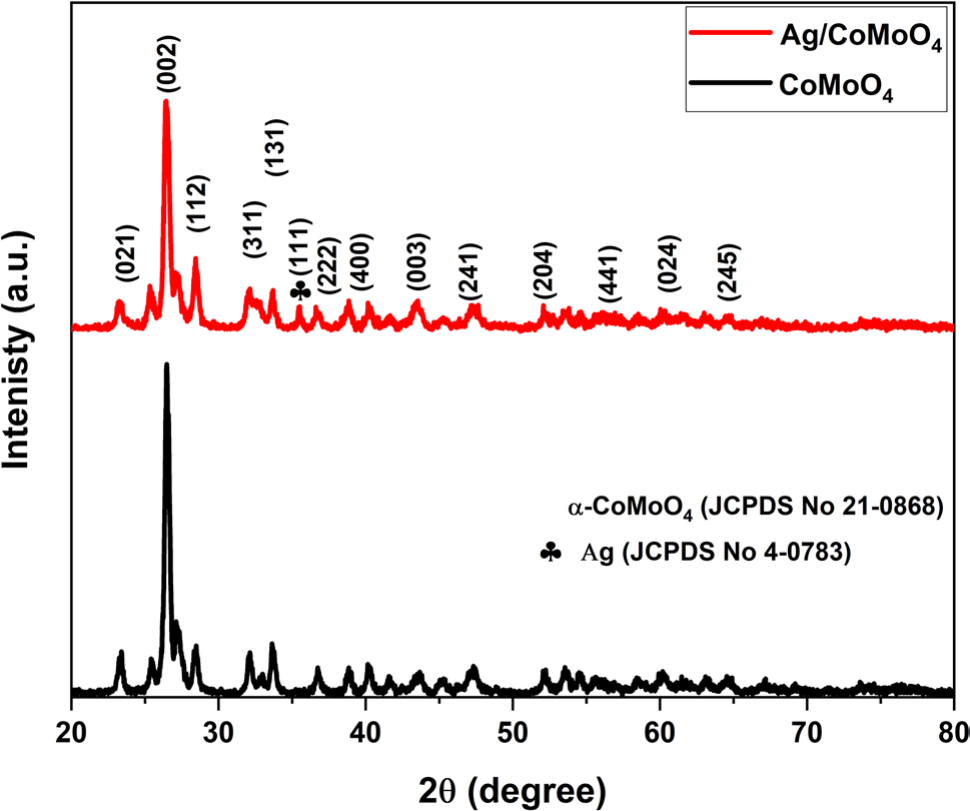
XRD analysis of CoMoO_4_ and Ag/CoMoO_4_ samples.

As shown in Fig. 1, XRD patterns of samples were well matched with monoclinic structure of pure α-CoMoO_4_ with space group *C*2/*m* (JCPDS No. 21-0868)^25^. After deposition of Ag NPs on CoMoO_4_, new crystal plane (111) was appeared that suggests the existence of cubic Ag NPs with JCPDS No. 4-0783^34^. In addition, the intensities of XRD peaks were slightly reduced in Ag/CoMoO_4_ sample. Besides, it should be noted that the peak position of CoMoO_4_ was not shifted, which suggested no substitutional doping. No impurities diffraction peaks were found in samples. The existence of Ag and CoMoO_4_ in Ag/CoMoO_4_ suggest the successful fabrication of heterostructures.

**Figure 2:**
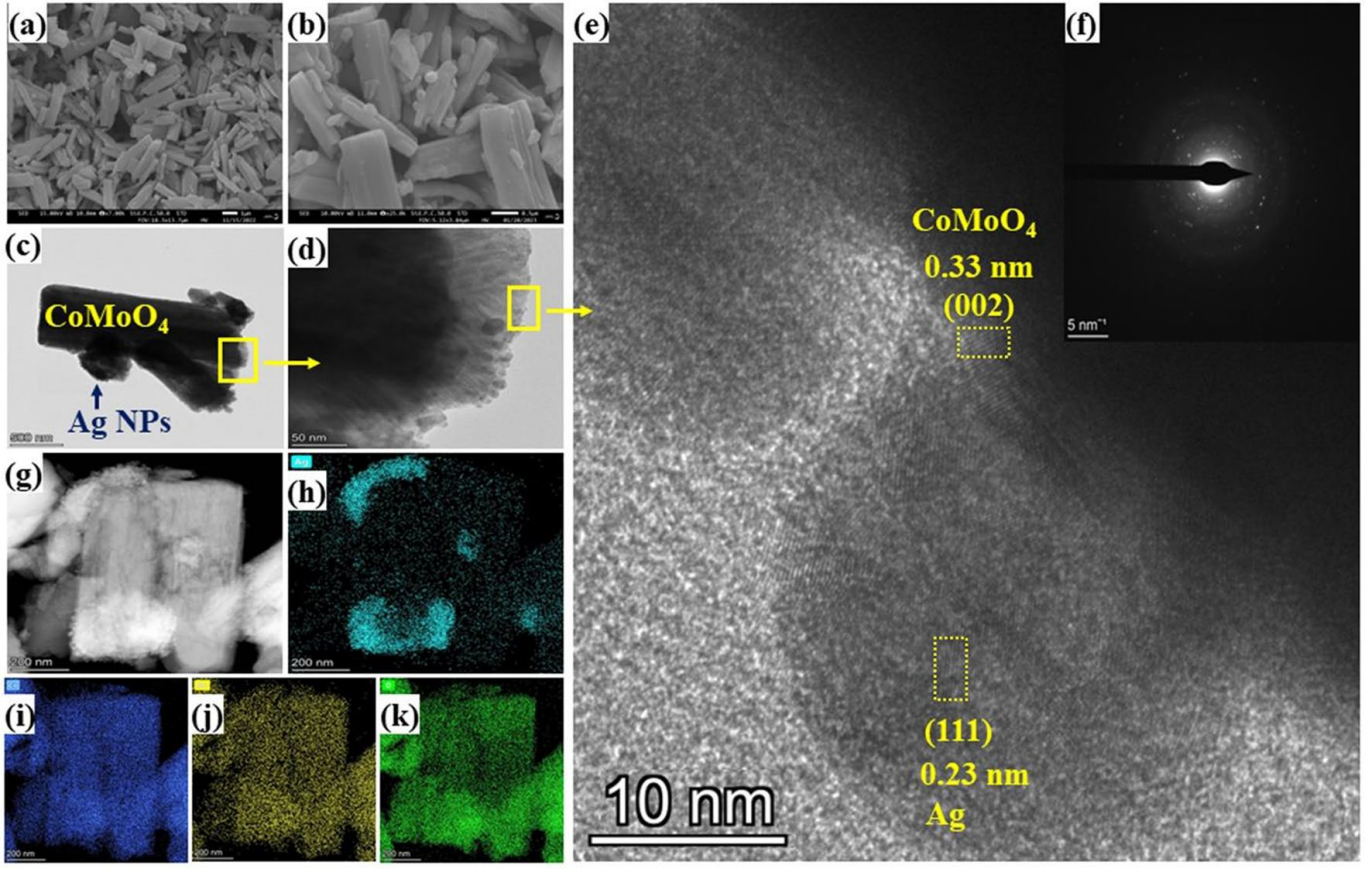
FESEM of (**a**) CoMoO_4_ and (**b**) Ag/CoMoO_4_), (**c** and **d**) TEM, (**e**) HRTEM, (**f**) SAED pattern, (**g**–**k**) TEM elemental mapping (**g**: EDS layered image, **h**: Ag, **i**: Co, **j**: Mo, and **k**: O) images of Ag/CoMoO_4_. Scale bar (**a**: 1 μm, b: 0.5 μm, **c**: 500 nm, **d**: 50 nm, **e**: 10 nm, **f**: 5 nm^−1^, and **g**–**k**: 200 nm).

The morphologies of synthesized materials were investigated by FESEM and TEM. The samples presented rod-like morphology with dimensions of 1–3 μm in length and 0.3–1 μm in width (Fig. 2a and b). The existence of Ag NPs on the CoMoO_4_ rods was also observed in Fig. 2b. In addition, the loading of Ag NPs on CoMoO_4_ rods did not change the morphology of materials. Figure 2c and d presented the TEM image of Ag/CoMoO_4_. This image revealed the decoration of Ag NPs on the surface of CoMoO_4_ rods. It also indicates the uniform distribution of Ag NPs on rods. The interplanar spacing of 0.33 nm and 0.23 nm calculated from Fig. 2e were corresponds to the (002) and (111) crystal planes of CoMoO_4_ and Ag, respectively which are also strongest peak in the XRD spectrum. All the interplanar spacing calculated from HRTEM image are well consistent with crystallographic plane of CoMoO_4_ and Ag. SAED patterns suggested the poly-crystalline nature of Ag/CoMoO_4_ (Fig. 2f). As shown in Figs. 2g–k and S5, the TEM-EDS mapping/spectrum of Ag/CoMoO_4_ indicated the existence as well as homogenous distribution of Co, Mo, O, and Ag (Table S1). According to the results of XRD, FESEM, TEM, HRTEM, SAED, and TEM-EDS elemental mapping images, it was concluded that the heterostructure was successfully formed between CoMoO_4_ and Ag NPs.

**Figure 3:**
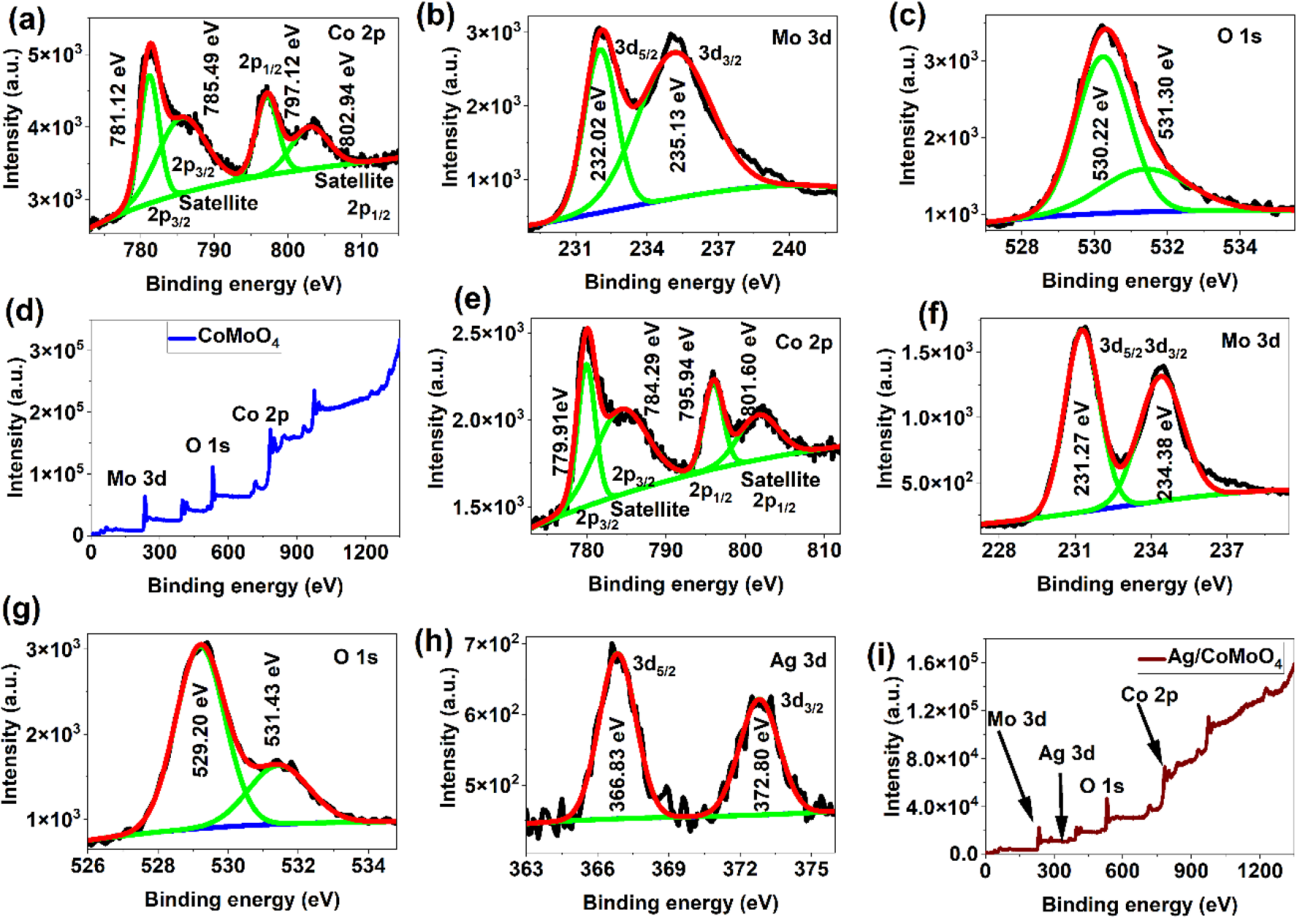
XPS spectra of CoMoO_4_ (**a**–**d**) and Ag/CoMoO_4_ (**e**–**i**).

The existence of elements and oxidation states in CoMoO_4_ and Ag/CoMoO_4_ were investigated using XPS technique (Fig. 3). The Co 2p spectra of samples could be deconvoluted into 2p_3/2_, (CoMoO_4_: 781.12 eV and Ag/CoMoO_4_: 779. 91 eV), satellite 2p_3/2_ (CoMoO_4_: 785.49 eV and Ag/CoMoO_4_: 784.29 eV), 2p_1/2_, (CoMoO_4_: 797.12 eV and Ag/CoMoO_4_: 795.94 eV) and satellite 2p_1/2_ (CoMoO_4_: 802.94 eV and Ag/CoMoO_4_: 801.60 eV) peaks that suggests the Co^2+^ oxidation state in samples (Fig. 3a and e)^35^. The Mo 3d spectra were fitted into two peaks 3d_5/2_ (CoMoO_4_: 232.02 eV and Ag/CoMoO_4_: 231.27 eV) and 3d_3/2_ (CoMoO_4_: 235.13 eV and Ag/CoMoO_4_: 234.38 eV). It clearly shows the presence of Mo^6+^ in samples (Fig. 3b and f)^36^. In addition, 0.75 eV and 1.2 eV shift of the binding energy were observed in Mo 3d and Co 2p peaks, respectively. It suggests the evidence for interaction between the CoMoO_4_ and Ag^37^. According to XPS results of O1s spectra, O^2−^ species in lattice (CoMoO_4_: 530.22 eV and Ag/CoMoO_4_: 529.20 eV) and oxygen vacancies (CoMoO_4_: 531.30 eV and Ag/CoMoO_4_: 531.30 eV) or defects were observed (Fig. 3c and g)^38^. As shown in Fig. 3h, the presence of metallic Ag NPs in Ag/CoMoO_4_ was proved by 3d_5/2_ and 3d_3/2_ peaks at 366.83 eV and 372.80 eV, respectively^39^. Furthermore, the survey spectra suggested the confirmation of Co, Mo, O, and Ag in samples (Fig. 3c and g). Also, the results of ICP-OES indicated the presence of metallic ions (Co, Mo, and Ag) in samples (Supporting information). To find the surface charge in samples, the zeta potential of CoMoO_4_ and Ag/CoMoO_4_ was evaluated. The zeta potential of CoMoO_4_ and Ag/CoMoO_4_ was  − 12.30 mV and  − 15.33 mV, respectively. This results suggests that the surface of both samples are negatively charged.

Raman spectra of CoMoO_4_ and Ag/CoMoO_4_ was shown in Fig. S6. The vibrational modes were found at 926, 869, 808.70, and 355.61 cm^−1^. The Raman mode located at 926.51 cm^−1^ was associated with symmetric stretching mode of doubly coordinated bridging oxygen in Mo–O^40^. The band at 869.20 cm^−1^ was related to the symmetric stretching of Co–O–Mo bond. In addition, the band observed at 808.08 cm^−1^ can be attributed to the asymmetric stretching mode of oxygen in O–Mo–O^41^. The symmetry bending modes of O–Mo–O was observed at 355.61 cm^−1^^42^. The decoration of Ag NPs on CoMoO_4_ did not alter the Raman bands that suggest the fabrication of heterojunction between Ag NPs and CoMoO_4_. Furthermore, FTIR studies were performed of CoMoO_4_ and Ag/CoMoO_4_ over the range 500–4000 cm^−1^ (Fig. S7). The band in lower frequency region (CoMoO_4_: 692.90 cm^−1^ and Ag/CoMoO_4_: 632.96 cm^−1^) was associated with Co–Mo–O stretching vibrations^43^. The peaks appeared in CoMoO_4_ (779.75, 832.78, and 926.54 cm^−1^) and Ag/CoMoO_4_ (779.76, 846.01, and 933.40 cm^−1^) were assigned to Mo–O stretching bands^44^. These bands provided the evidence of CoMoO_4_ in samples.

### Electrochemical CO_2_ reduction

**Figure 4:**
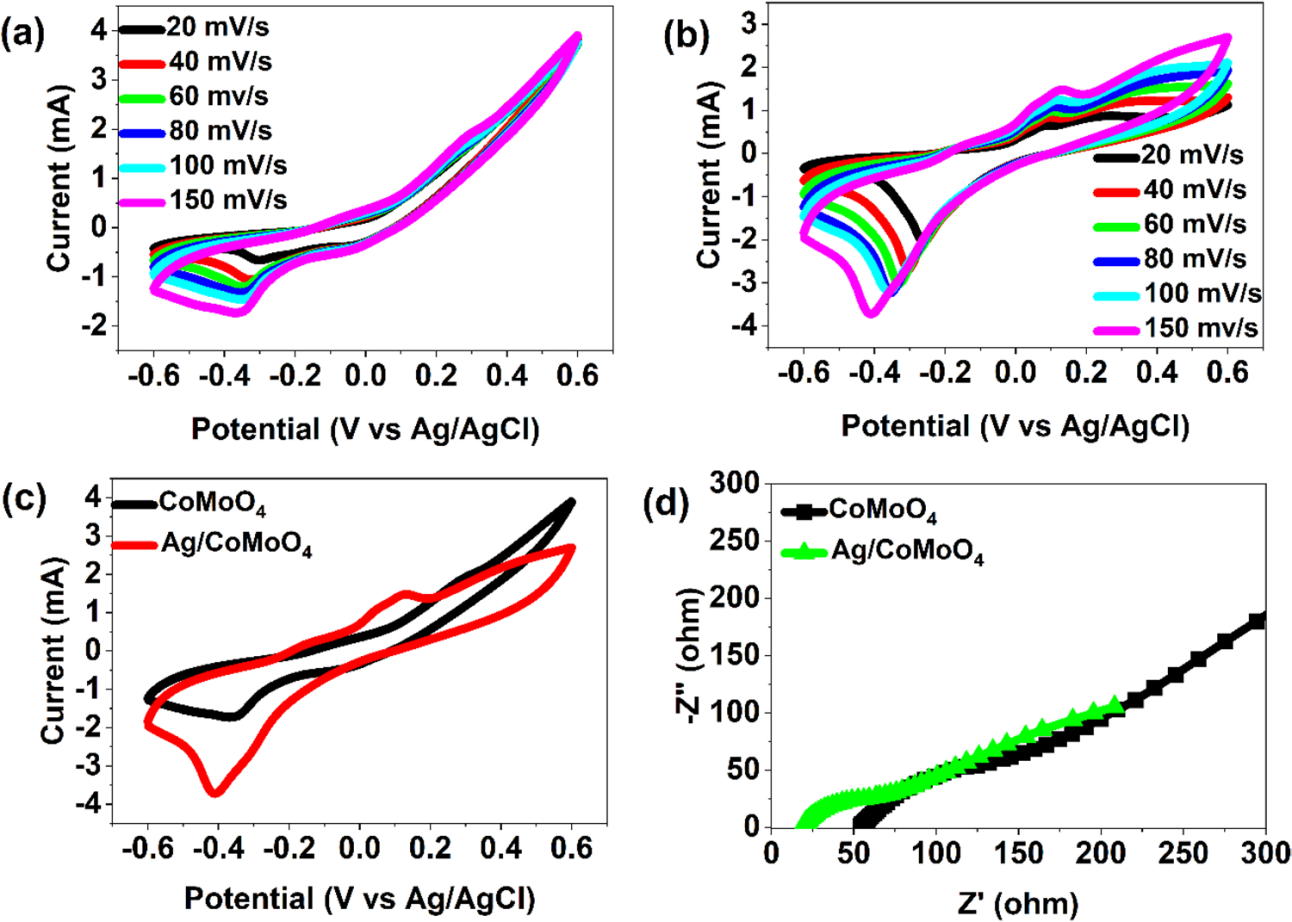
CV (**a**: CoMoO_4_, (**b**) Ag/CoMoO_4_, and (**c**) comparison of CoMoO_4_ and Ag/CoMoO_4_) and EIS (**d**) of samples.

As shown in Fig. 4a and b, CV curves of CoMoO_4_ and Ag/CoMoO_4_ nanorods were recorded in a potential window of  − 0.6 to 0.6 V at different sweeping rates (20 mV/s, 40 mV/s, 60 mV/s, 80 mV/s, 100 mV/s, and 150 mV/s). The observed redox peaks may be attributed to reversibly changing their oxidation states of Co^2+^ and Co^3+^^45^. These redox peaks were obtained from redox mechanism that reveals the Faradic capacitive behavior of the CoMoO_4_ and Ag/CoMoO_4_ electrodes. In addition, the enhancement of conductivity by molybdenum (Mo) can improve the electrochemical performances of electrodes^28^. Also, an increase in sweep rate provided the shifting of the oxidation and reduction peaks of electrodes towards right and left, respectively due to higher internal diffusion resistance. The CV curve area and current increased with increase in scan rate because of fast reaction kinetics^27,46^. The shape of CV peaks did not change at high scan rate that suggests the good rate performance of catalyst.

As depicted in Fig. 4c, compared with CoMoO_4_ electrode, the increased loop of CV curves was observed for Ag/CoMoO_4_ electrodes. In addition, presence of Ag NPs in CoMoO_4_ enhanced the reduction ability. These factor indicate the improvement of charge transfer and transportation of ions by making heterostructure between Ag NPs and CoMoO_4_. So, the fabrication of heterojunction between Ag NPs and CoMoO_4_ rods enhanced the electrocatalytic performance which is beneficial for CO_2_ reduction. To observe the effect of Cu-foil in fabricated electrodes, CV curve of Cu-foil was carried out (Fig. S8). It demonstrates the negligible current as compared to CoMoO_4_ and Ag/CoMoO_4_ CV curves. Also, insignificant contribution of Cu-foil was noted. Also, EIS was measured to observe the interfacial charge transfer on catalysts (Fig. 4d)^47^. The obtained data was fitted, and equivalent circuit was made (Fig. S9). It was composed of solution resistance (R1), charge transfer resistance (R2), electric double layer capacitance (C2), Warburg impedance, and constant phase element (Q). According to the Nyquist plots, Ag/CoMoO_4_ (41.57 Ω) demonstrated lower charge transfer resistance in comparison to CoMoO_4_ (309.50 Ω) suggesting its rapid electron transfer between the interface of electrolyte and electrocatalyst that may allow efficient electron, Ag NPs and CoMoO_4_ interactions (Table S2). Therefore, decoration of Ag NPs on CoMoO_4_ rods could promote the electron transportation between the electrocatalyst and CO_2_ molecules that provides the electrochemical reduction capability of heterostructure.

**Figure 5:**
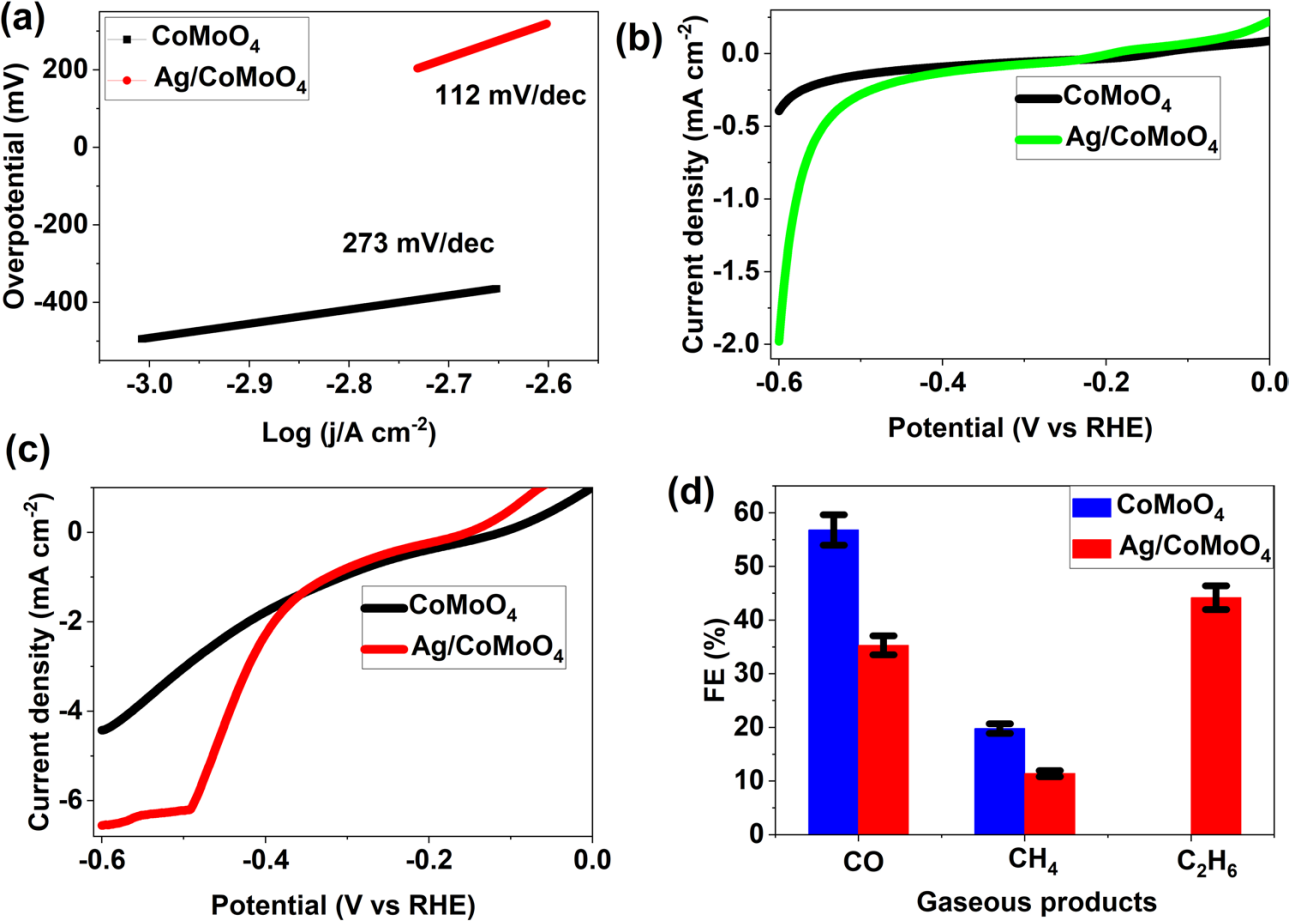
Tafel plots (**a**), LSV (**b**: before CO_2_ saturated and **c**: after CO_2_ saturated in 0.1 M KHCO_3_), and FEs (**d**) of samples at  − 1.3 V versus RHE.

The accelerated CO_2_RR conversion kinetics upon the heterostructure was further conformed by Tafel plots (Fig. 5a). The Tafel slope for CoMoO_4_ and Ag/CoMoO_4_ were estimated to be 273 mV/dec and 112 mV/dec, respectively. The lowest Tafel slope for Ag/CoMoO_4_ suggests the enhancement of electrocatalytic activity by fabricating heterostructure between Ag NPs and CoMoO_4_ because of rapid electron transfer from the electrode to electrocatalyst. This result also indicates that the transfer of first electron to adsorbed CO_2_ molecules. It facilitates the production *CO_2_ that can improve a second electron-transfer for *COOH generation^48^. To compare the electrochemical performance of Ag NPs with other non-precious metal particles, Tafel slope was evaluated (Fig. S10). Ag NPs showed lower Tafel slope than Cu indicating great electrochemical performance of Ag NPs that is accordance to published report^49^. Furthermore, the CO_2_RR performance of the as-synthesized electrocatalysts was investigated by LSV graphs (Fig. 5c and d). Ag/CoMoO_4_ revealed higher current density than CoMoO_4_ at  − 0.6 V. Ag NPs decorated CoMoO_4_ rods showed approximately fivefold enhancement of current density in comparison with CoMoO_4_ rods (Fig. 5b). The current density of samples along with ﻿CO_2_-saturated 0.1 M KHCO_3_ electrolyte suggest demonstrated higher current density suggesting better reactivity in CO_2_RR (Fig. 5c).

Steady-state current responses in a CO_2_-saturated electrolyte for 400 s at  − 1.3 V versus RHE of samples were presented in Fig. S11. The obtained current densities for CoMoO_4_ and Ag/CoMoO_4_ were  − 3.35 mA and  − 4.62 mA, respectively. After that, the gas-phase products were detected by using a GC (Table S3). The FEs for various gas formation of CoMoO_4_ and Ag/CoMoO_4_ were calculated (Supporting Information)^33^. According to Fig. 5d, CoMoO_4_ presented FEs for CO and CH_4_ were 56.80% and 19.80%, respectively. In this case, CO and CH_4_ act as a major and minor gaseous products during CO_2_RR, respectively. In addition, Ag/CoMoO_4_ revealed FEs for CO and CH_4_, and C_2_H_6_ were 35.30%, 11.40%, and 44.20%, respectively. It was noted that loading of Ag NPs on CoMoO_4_ reduces the FEs for CO and CH_4_, and C_2_H_6_. These results clearly indicate that C_2_H_6_ is a major product in Ag NPs decorated CoMoO_4_ rods. In addition, electrocatalytic stability of cu-foil was evaluated after a 400 s amperometric test at  − 1.3 V versus RHE (Fig. S12). Low current density along with unstable nature were observed. It suggests the Cu-foil did not contribute the significantly for steady-state current responses. Stability curves of Ag/CoMoO_4_ at  − 1.3 V versus RHE was shown in Fig. S13. Notably, the electrocatalyst exhibited outstanding stability even upto 25 h. Also, the current density was not changed during stability. ICP-OES analysis of electrolyte solution revealed no leaching of Ag ions after 25 h stability test. Table 1 revealed the comparison of FEs of various Ag-based electrocatalysts with Ag/CoMoO_4_^16–19,34,48,50,51^. This Table suggests the fabrication of various morphologies of Ag and Ag-based heterostructure by several techniques for electrochemical reduction of CO_2_ to CO and C_2_H_4_ under different applied potential. Although several gaseous products were found on Ag-based electrocatalysts, electrocatalytic reduction of CO_2_ into C_2_H_6_ by using Ag/CoMoO_4_ heterostructure has not yet been reported in the literature.

**Table 1:** The comparison of the electrochemical CO_2_RR results of this work with other Ag-based electrocatalysts.

Catalyst	Synthesis methods	Morphology	FEs	Potential (V vs. RHE/SCE)	References
Ag-Au	Self-assembly	Nanowires/nanosheet	99.0% (CO)	− 0.9	^50^
Ag	Chemical/photoreduction	Triangular/nanoplates	96.0% (CO)	− 0.856	^51^
Ag@ZnO@rGO	Hydrothermal/functionalization	Dodecahedral	70.0% (CO)	− 1.6	^34^
Ag/CuO	Hydrothermal and impregnation	Nanosphere	40.0% (C_2_H_4_)	− 1.1	^48^
Ag/CuO	Solution-phase	Nanosheet	91.2% (CO)	− 0.7	^16^
Ag/Cr_2_O_3_	Electrochemical	Hexagon	99.6% (CO)	− 0.8	^17^
Ag/SnO_2_	Oil bath	Sphere	99.2%(CO)	− 0.9	^18^
Ag/MOx (M = Cr, Sn, Bi, Cu, Pb, and Mn)	Reduction	Dendritic	98.0% (CO)	− 0.7	^19^
Ag/CoMoO_4_	Hydrothermal and photoreduction	Rods and nanoparticles	35.30% (CO), 11.40% (CH_4_), and 44.20% (C_2_H_6_)	− 1.3	Our work

Based on the above results, the possible reaction mechanisms/pathways were purposed. In CoMoO_4_, Co consists of loosely bonded d-electrons that provides the multiple oxidation state. Moreover, transition of Co (II) to Co (I) is considered as an intermediate state for CO_2_ reduction^26^. At first, Co^2+^ is reduced to Co^+^ under the applied potential. When CO_2_ is adsorbed on the (002) catalyst surface, oxidation of Co^+^ to Co^2+^ is occurred. Due to this reason, electron is transferred to the adsorbed CO_2_ and stabilization of CO_2_ radical is happened. Also, the presence of oxygen vacancy in catalyst can improve the stabilization of CO_2_ radical^15^. After that, CO_2_ radical has ability to capture the proton (H^+^) and electron (e^−^) that may dissociate from HCO_3_^−^ ion to produce COOH* intermediate because of small potential obstacles. Then, this intermediate reacted with H^+^/e^−^ continuously to generate CO molecule. The possible reasons for formation of CO as a major product is related with existence of π-back donation between the center of Co metal and CO_2_ ligand that can enhance the C-O cleavage^52^. Furthermore, *CO may transform into *CHO via hydrogenation^53^. The stabilization of *CHO intermediate play significant role for mitigating the overpotential for CH_4_ production. This *CHO intermediate may convert into *CH_2_O and *CH_3_O during transfer of H^+^/e^−^ during CO_2_RR. Finally, *CH_3_O intermediate transforms into CH_4_^53,54^.

The decoration of Ag NPs (111) on the surface of CoMoO_4_ rod may reduce the energy barrier for conversion of CO_2_ to CO, CH_4_ and C_2_H_6_ products that can change the reactions pathways. C–C coupling mechanism plays a vital role to achieve the high selectivity of C_2_H_6_ species. The possible reason for generation of C_2_H_6_ species may associate with existence of active sites in catalyst. According to this mechanism, *CO dimerization process is occurred at the catalyst surface^53,55^. The double bond between the C and O is broken and proton can attack the O site to form -OH during reaction with H^+^/e^−^. In addition, -OH functional group is eliminated with the reaction with proton to produce H_2_O. Due to this reaction, double bonds are created between C to C and C to O. After transfer of two H^+^/e^−^, 2H^+^ react with carbon to form HC=CH along with attachment of O on the surface of catalyst. Then, H^+^ may react with double bond containing C to form single bond between carbon along with attachment of O with surface and -CH_2_. At last, surface attached O reacts with H^+^ to form H_2_O and C_2_H_6_ is produced. The possible reason for obtaining the higher FEs of C_2_H_6_ than CO and CH_4_ for Ag/CoMoO_4_ may associate with higher chance of protonation (*CO → *COH) than desorption of *CO on interface^56^. Also, decrease in FEs for CO and CH_4_ of Ag/CoMoO_4_ than COMoO_4_ could be related with covering the Ag NPs on CoMoO_4_ rods. The synergistic effect between Ag NPs and CoMoO_4_ rods can be attributed to generate the CO, CH_4_ and C_2_H_6_.

## Conclusion

Ag/CoMoO_4_ electrocatalyst was prepared through hydrothermal and photoreduction/deposition methods. The existence of heterostructure between Ag NPs and CoMoO_4_ rods was shown by structural and physicochemical characterization techniques. The excellent electrochemical behaviors of catalysts were proved by CV, EIS, LSV, chronoamperometry, and Tafel plots. The electrochemical CO_2_RR of CoMoO_4_ favored for CO (FEs: 56.80) and CH_4_ (FEs: 19.80) at  − 1.3 V versus RHE in a H-type cell containing 0.5 M KHCO_3_. However, the heterostructure revealed selectivity for reducing mainly CO_2_ to C_2_H_6_ (FEs: 56.80) along with lower FEs for CO and CH_4_ at same condition. The selectivity for reducing CO_2_ to CO, CH_4_, and C_2_H_6_ by electrocatalyst was attributed to adequate active sites, oxygen vacancies, and excellent conductivities. In addition, the synergistic effect of Ag-CoMoO_4_ active sites provided the C–C coupling for reduction of CO_2_ to C_2_H_6_ electrocatalytically. The electrocatalyst showed excellent stability upto 25 h without reduction of current density that can be applied for practical application towards electrocatalytic CO_2_ reduction. The possible mechanisms/pathways were proposed for CO_2_RR. Finally, the outcomes of this work present a new approach for improving electrochemical performances/reduction of CO_2_ to CO and hydrocarbon by using the Ag/CoMoO_4_ heterostructure catalyst.

### Supplementary Information

Supplementary Information 1.

Supplementary Information 2.

## Data Availability

The data generated or analysed during this study are available within the article and its supplementary material. Raw data of cyclic voltammetry, Electrochemical Impedance, Faradaic Efficiency, IT curves, LSV plot, Tafel plot, Raman, XPS and XRD spectra are provided in supplementary material (Raw data). All other data is available from the corresponding author upon request.
