# Supplementary material for: Decoration of Ag nanoparticles on CoMoO4 rods for efficient electrochemical reduction of CO2
Source: Sci Rep. 2024 Jan 16;14:1406. doi: 10.1038/s41598-024-51680-w (PMC10792071; doi:10.1038/s41598-024-51680-w)
Supplement: Supplementary file 1 — Supplementary Information 1. [file 41598_2024_51680_MOESM1_ESM.zip › raw data for scientific reports/XRD raw data.DOCX]

**XRD raw data of samples**

**CoMoO4**

***RAS_DATA_START --**

***RAS_HEADER_START --**

***CORR_POS_DB_NAME "Si**

***CORR_POS_GONIO_RADIUS ""**

***CORR_POS_NODE_INDEX01 ""**

***CORR_POS_NODE_INDEX02 ""**

***CORR_POS_NODE_INDEX03 ""**

***CORR_POS_NODE_INDEX04 ""**

***CORR_POS_NODE_INDEX05 ""**

***CORR_POS_NODE_INDEX06 ""**

***CORR_POS_NODE_INDEX07 ""**

***CORR_POS_NODE_INDEX08 ""**

***CORR_POS_NODE_INDEX09 ""**

***CORR_POS_NODE_INDEX10 ""**

***CORR_POS_NODE_INDEX11 ""**

***CORR_POS_NODE_LABEL01 "Si(111)"**

***CORR_POS_NODE_LABEL02 "Si(220)"**

***CORR_POS_NODE_LABEL03 "Si(311)"**

***CORR_POS_NODE_LABEL04 "Si(400)"**

***CORR_POS_NODE_LABEL05 "Si(331)"**

***CORR_POS_NODE_LABEL06 "Si(422)"**

***CORR_POS_NODE_LABEL07 "Si(511)"**

***CORR_POS_NODE_LABEL08 "Si(440)"**

***CORR_POS_NODE_LABEL09 "Si(531)"**

***CORR_POS_NODE_LABEL10 "Si(620)"**

***CORR_POS_NODE_LABEL11 "Si(533)"**

***CORR_POS_NODE_POS_CALC01 "28.377"**

***CORR_POS_NODE_POS_CALC02 "47.2506"**

***CORR_POS_NODE_POS_CALC03 "56.0725"**

***CORR_POS_NODE_POS_CALC04 "69.0822"**

***CORR_POS_NODE_POS_CALC05 "76.3294"**

***CORR_POS_NODE_POS_CALC06 "87.9853"**

***CORR_POS_NODE_POS_CALC07 "94.9091"**

***CORR_POS_NODE_POS_CALC08 "106.668"**

***CORR_POS_NODE_POS_CALC09 "114.054"**

***CORR_POS_NODE_POS_CALC10 "127.511"**

***CORR_POS_NODE_POS_CALC11 "136.864"**

***CORR_POS_NODE_POS_MEAS01 "28.3931"**

***CORR_POS_NODE_POS_MEAS02 "47.2458"**

***CORR_POS_NODE_POS_MEAS03 "56.0729"**

***CORR_POS_NODE_POS_MEAS04 "69.0852"**

***CORR_POS_NODE_POS_MEAS05 "76.3288"**

***CORR_POS_NODE_POS_MEAS06 "87.9726"**

***CORR_POS_NODE_POS_MEAS07 "94.8953"**

***CORR_POS_NODE_POS_MEAS08 "106.657"**

***CORR_POS_NODE_POS_MEAS09 "114.038"**

***CORR_POS_NODE_POS_MEAS10 "127.496"**

***CORR_POS_NODE_POS_MEAS11 "136.855"**

***CORR_POS_OPT_ATTR "W@"**

***CORR_POS_PARAM_MU ""**

***CORR_POS_PARAM_PL ""**

***CORR_POS_SEMODEL_TYPE "0"**

***CORR_POS_STANDARD_TYPE ""**

***CORR_POS_VALUE_ANALYZER "None"**

***CORR_POS_VALUE_CMONO "None"**

***CORR_POS_VALUE_IMONO "None"**

***CORR_POS_VALUE_IS "1.250deg"**

***CORR_POS_VALUE_ISL "10.0mm"**

***CORR_POS_VALUE_ISOLLER "5.0deg"**

***CORR_POS_VALUE_RS1 "1.250deg"**

***CORR_POS_VALUE_RS2 "0.3mm"**

***CORR_POS_VALUE_RSOLLER "5.0deg"**

***CORR_POS_XG_FOCUS_L ""**

***CORR_POS_XG_WAVE_TYPE ""**

***CORR_POS_XG_WAVE_VALUE ""**

***DISP_FMT_X "%.2f"**

***DISP_FMT_Y "%.0f"**

***DISP_FULLSCALE "635.0000000000"**

***DISP_LINE_COLOR "255.0000000000"**

***DISP_LINE_STYLE "0.0000000000"**

***DISP_LINE_WIDTH "0.2500000000"**

***DISP_NOTE "Collected**

***DISP_OFFSET_Y "0.0000000000"**

***DISP_PEAKSEARCH_UNIT_Y "cps"**

***DISP_RANGE_BOTTOM "0.0000000000"**

***DISP_RANGE_LEFT "10.0000000000"**

***DISP_RANGE_LOGCUTOFF "0.0100000000"**

***DISP_RANGE_RIGHT "80.0000000000"**

***DISP_RANGE_TOP "635.0000000000"**

***DISP_SCALE_MODE_Y "0.0000000000"**

***DISP_TAB_NAME "Theta/2-Theta"**

***DISP_TITLE_Y "Intensity"**

***DISP_UNIT_Y "cps"**

***DISP_WINDOW_NAME "MSG_MACROENGINE"**

***FILE_COMMENT "**

***FILE_MD5 ""**

***FILE_MEMO "**

***FILE_OPERATOR "Administrator"**

***FILE_SAMPLE "CoMoO4pH7"**

***FILE_TYPE "RAS_RAW"**

***FILE_USERGROUP "System**

***FILE_VERSION "1.0000000000"**

***HW_ATTACHMENT_ID "ATT1025"**

***HW_ATTACHMENT_NAME "W¿ä|Standard**

***HW_COUNTER_ID-0 "CUT0021"**

***HW_COUNTER_ID-1 "CUT0020"**

***HW_COUNTER_ID-2 "CUT0020"**

***HW_COUNTER_ID-3 "CMC0021"**

***HW_COUNTER_NAME-0 "SC-70|SC-70"**

***HW_COUNTER_NAME-1 "None|None"**

***HW_COUNTER_NAME-2 "None|None"**

***HW_COUNTER_NAME-3 "oímN[^|Detector**

***HW_COUNTER_SELECT_NAME "SC-70|SC-70"**

***HW_ECP_HV "700.0000000000"**

***HW_ECP_HV_UNIT "V"**

***HW_GONIOMETER_ID "GON0070"**

***HW_GONIOMETER_NAME "MiniFlex**

***HW_GONIOMETER_RADIUS-0 "79.0"**

***HW_GONIOMETER_RADIUS-1 "91.5"**

***HW_GONIOMETER_RADIUS-2 "150.0"**

***HW_GONIOMETER_RADIUS-3 "0"**

***HW_GONIOMETER_RADIUS-4 "103.4"**

***HW_GONIOMETER_RADIUS-5 "150.0"**

***HW_GONIOMETER_RADIUS-6 "46.6"**

***HW_GONIOMETER_RADIUS-7 "160.0"**

***HW_I_OPT_ID-0 "ISL0021"**

***HW_I_OPT_ID-1 "ISO0021"**

***HW_I_OPT_NAME-0 "­UXbg|Divergence**

***HW_I_OPT_NAME-1 "üË½sXbg|Incident**

***HW_R_ATTENUATER_AUTOMODE "0"**

***HW_R_OPT_ID-0 "RSS0021"**

***HW_R_OPT_ID-1 "RSO0021"**

***HW_R_OPT_ID-2 "RRS0021"**

***HW_R_OPT_NAME-0 "UXbg|Scattering**

***HW_R_OPT_NAME-1 "óõ½sXbg|Receiving**

***HW_R_OPT_NAME-2 "óõXbg|Receiving**

***HW_SAMPLE_HOLDER_ID "SMP0000"**

***HW_SAMPLE_HOLDER_NAME "³µ|None"**

***HW_XG_CURRENT_MAX "15"**

***HW_XG_CURRENT_MIN "2"**

***HW_XG_CURRENT_RESOLUTION "1"**

***HW_XG_CURRENT_UNIT "mA"**

***HW_XG_FOCUS "1mm**

***HW_XG_FOCUS_TYPE "Normal"**

***HW_XG_ID ""**

***HW_XG_NAME "300W/600W**

***HW_XG_TARGET_ATOMIC_NUMBER "74.0000000000"**

***HW_XG_TARGET_NAME "W"**

***HW_XG_TYPE "Hermetic"**

***HW_XG_VOLTAGE_MAX "40"**

***HW_XG_VOLTAGE_MIN "20"**

***HW_XG_VOLTAGE_RESOLUTION "1"**

***HW_XG_VOLTAGE_UNIT "kV"**

***HW_XG_WATT_MAX "0.600"**

***HW_XG_WATT_UNIT "kW"**

***HW_XG_WAVE_LENGTH_ALPHA1 "0.209013"**

***HW_XG_WAVE_LENGTH_ALPHA2 "0.213831"**

***HW_XG_WAVE_LENGTH_BETA "0.184377"**

***MEAS_COND_AXIS_NAME-0 "Theta/2-Theta"**

***MEAS_COND_AXIS_NAME-1 "Soller(inc.)"**

***MEAS_COND_AXIS_NAME-2 "IHS"**

***MEAS_COND_AXIS_NAME-3 "DS"**

***MEAS_COND_AXIS_NAME-4 "SS"**

***MEAS_COND_AXIS_NAME-5 "Soller(rec.)"**

***MEAS_COND_AXIS_NAME-6 "RS"**

***MEAS_COND_AXIS_NAME-7 "Filter"**

***MEAS_COND_AXIS_NAME-8 "Monochromator"**

***MEAS_COND_AXIS_NAME-9 "HV"**

***MEAS_COND_AXIS_NAME-10 "PHA"**

***MEAS_COND_AXIS_NAME_INTERNAL-0 "TwoThetaTheta"**

***MEAS_COND_AXIS_NAME_INTERNAL-1 "IncidentSollerSlit"**

***MEAS_COND_AXIS_NAME_INTERNAL-2 "IncidentAxdSlit"**

***MEAS_COND_AXIS_NAME_INTERNAL-3 "IncidentSlitBox"**

***MEAS_COND_AXIS_NAME_INTERNAL-4 "ReceivingSlitBox1"**

***MEAS_COND_AXIS_NAME_INTERNAL-5 "ReceivingSollerSlit"**

***MEAS_COND_AXIS_NAME_INTERNAL-6 "ReceivingSlitBox2"**

***MEAS_COND_AXIS_NAME_INTERNAL-7 "Filter"**

***MEAS_COND_AXIS_NAME_INTERNAL-8 "CounterMonochromator"**

***MEAS_COND_AXIS_NAME_INTERNAL-9 "HV"**

***MEAS_COND_AXIS_NAME_INTERNAL-10 "PHA"**

***MEAS_COND_AXIS_NAME_MAGICNO-0 "268632064.0000000000"**

***MEAS_COND_AXIS_NAME_MAGICNO-1 "20971521.0000000000"**

***MEAS_COND_AXIS_NAME_MAGICNO-2 "22020097.0000000000"**

***MEAS_COND_AXIS_NAME_MAGICNO-3 "22020097.0000000000"**

***MEAS_COND_AXIS_NAME_MAGICNO-4 "23068673.0000000000"**

***MEAS_COND_AXIS_NAME_MAGICNO-5 "20971521.0000000000"**

***MEAS_COND_AXIS_NAME_MAGICNO-6 "23068673.0000000000"**

***MEAS_COND_AXIS_NAME_MAGICNO-7 "20971521.0000000000"**

***MEAS_COND_AXIS_NAME_MAGICNO-8 "20971521.0000000000"**

***MEAS_COND_AXIS_NAME_MAGICNO-9 "0.0000000000"**

***MEAS_COND_AXIS_NAME_MAGICNO-10 "0.0000000000"**

***MEAS_COND_AXIS_OFFSET-0 "0.0000000000"**

***MEAS_COND_AXIS_OFFSET-1 "-"**

***MEAS_COND_AXIS_OFFSET-2 "-"**

***MEAS_COND_AXIS_OFFSET-3 "-"**

***MEAS_COND_AXIS_OFFSET-4 "-"**

***MEAS_COND_AXIS_OFFSET-5 "-"**

***MEAS_COND_AXIS_OFFSET-6 "-"**

***MEAS_COND_AXIS_OFFSET-7 "-"**

***MEAS_COND_AXIS_OFFSET-8 "-"**

***MEAS_COND_AXIS_OFFSET-9 "-"**

***MEAS_COND_AXIS_OFFSET-10 "-"**

***MEAS_COND_AXIS_POSITION-0 "10.0000000000"**

***MEAS_COND_AXIS_POSITION-1 "5.0deg"**

***MEAS_COND_AXIS_POSITION-2 "10.0mm"**

***MEAS_COND_AXIS_POSITION-3 "1.250deg"**

***MEAS_COND_AXIS_POSITION-4 "13.0mm"**

***MEAS_COND_AXIS_POSITION-5 "5.0deg"**

***MEAS_COND_AXIS_POSITION-6 "13.0mm"**

***MEAS_COND_AXIS_POSITION-7 "None"**

***MEAS_COND_AXIS_POSITION-8 "None"**

***MEAS_COND_AXIS_POSITION-9 "700.0000000000"**

***MEAS_COND_AXIS_POSITION-10 "500.0000000000"**

***MEAS_COND_AXIS_UNIT-0 "deg"**

***MEAS_COND_AXIS_UNIT-1 ""**

***MEAS_COND_AXIS_UNIT-2 ""**

***MEAS_COND_AXIS_UNIT-3 ""**

***MEAS_COND_AXIS_UNIT-4 ""**

***MEAS_COND_AXIS_UNIT-5 ""**

***MEAS_COND_AXIS_UNIT-6 ""**

***MEAS_COND_AXIS_UNIT-7 ""**

***MEAS_COND_AXIS_UNIT-8 ""**

***MEAS_COND_AXIS_UNIT-9 "V"**

***MEAS_COND_AXIS_UNIT-10 "mV"**

***MEAS_COND_COUNTER_CNTLOSS_CORR "0.0000000000"**

***MEAS_COND_COUNTER_PHABASE "500.0000000000"**

***MEAS_COND_COUNTER_PHAWINDOW "1000.0000000000"**

***MEAS_COND_COUNTER_PHA_UNIT "mV"**

***MEAS_COND_COUNTER_TAUDIFF "0.2500000000"**

***MEAS_COND_COUNTER_TAUINT "0.7500000000"**

***MEAS_COND_IRRADIATION_MODE "OFF"**

***MEAS_COND_IRRADIATION_WIDTH ""**

***MEAS_COND_OPT_ATTR "**

***MEAS_COND_OPT_NAME ""**

***MEAS_COND_UNSYMMETRIC_MODE "OFF"**

***MEAS_COND_XG_CURRENT "15.0000000000"**

***MEAS_COND_XG_VOLTAGE "40.0000000000"**

***MEAS_COND_XG_WAVE_TYPE "Ka"**

***MEAS_DATA_COUNT "3501.0000000000"**

***MEAS_SCAN_AXIS_X "Theta/2-Theta"**

***MEAS_SCAN_AXIS_X_INTERNAL "TwoThetaTheta"**

***MEAS_SCAN_END_TIME "02/16/23**

***MEAS_SCAN_MODE "CONTINUOUS"**

***MEAS_SCAN_MONITOR "0"**

***MEAS_SCAN_RESOLUTION_X "0.0025"**

***MEAS_SCAN_SPEED "1.0000"**

***MEAS_SCAN_SPEED_UNIT "deg/min"**

***MEAS_SCAN_START "10.0000000000"**

***MEAS_SCAN_START_TIME "02/16/23**

***MEAS_SCAN_STEP "0.0200000000"**

***MEAS_SCAN_STOP "80.0000000000"**

***MEAS_SCAN_UNIT_X "deg"**

***MEAS_SCAN_UNIT_Y "counts"**

***SLIT_SYSTEM "0"**

***RAS_HEADER_END --**

***RAS_INT_START --**

**10 11**

**10.02 5**

**10.04 7**

**10.06 7**

**10.08 10**

**10.1 8**

**10.12 11**

**10.14 12**

**10.16 9**

**10.18 6**

**10.2 12**

**10.22 8**

**10.24 10**

**10.26 12**

**10.28 12**

**10.3 7**

**10.32 10**

**10.34 9**

**10.36 9**

**10.38 6**

**10.4 6**

**10.42 7**

**10.44 9**

**10.46 10**

**10.48 6**

**10.5 7**

**10.52 7**

**10.54 8**

**10.56 12**

**10.58 10**

**10.6 8**

**10.62 9**

**10.64 14**

**10.66 12**

**10.68 9**

**10.7 8**

**10.72 8**

**10.74 11**

**10.76 10**

**10.78 10**

**10.8 12**

**10.82 6**

**10.84 8**

**10.86 9**

**10.88 8**

**10.9 9**

**10.92 12**

**10.94 6**

**10.96 8**

**10.98 5**

**11 7**

**11.02 9**

**11.04 5**

**11.06 10**

**11.08 10**

**11.1 9**

**11.12 8**

**11.14 8**

**11.16 5**

**11.18 14**

**11.2 13**

**11.22 8**

**11.24 7**

**11.26 9**

**11.28 11**

**11.3 9**

**11.32 12**

**11.34 8**

**11.36 7**

**11.38 6**

**11.4 12**

**11.42 10**

**11.44 6**

**11.46 10**

**11.48 3**

**11.5 14**

**11.52 8**

**11.54 11**

**11.56 9**

**11.58 11**

**11.6 9**

**11.62 11**

**11.64 8**

**11.66 6**

**11.68 11**

**11.7 14**

**11.72 11**

**11.74 11**

**11.76 12**

**11.78 11**

**11.8 8**

**11.82 13**

**11.84 4**

**11.86 7**

**11.88 8**

**11.9 10**

**11.92 7**

**11.94 9**

**11.96 14**

**11.98 13**

**12 13**

**12.02 10**

**12.04 11**

**12.06 11**

**12.08 8**

**12.1 8**

**12.12 9**

**12.14 10**

**12.16 7**

**12.18 13**

**12.2 11**

**12.22 11**

**12.24 9**

**12.26 11**

**12.28 7**

**12.3 6**

**12.32 8**

**12.34 10**

**12.36 13**

**12.38 11**

**12.4 5**

**12.42 11**

**12.44 8**

**12.46 12**

**12.48 7**

**12.5 10**

**12.52 14**

**12.54 13**

**12.56 9**

**12.58 7**

**12.6 9**

**12.62 12**

**12.64 10**

**12.66 14**

**12.68 11**

**12.7 16**

**12.72 7**

**12.74 8**

**12.76 12**

**12.78 12**

**12.8 14**

**12.82 14**

**12.84 15**

**12.86 13**

**12.88 17**

**12.9 19**

**12.92 21**

**12.94 24**

**12.96 19**

**12.98 27**

**13 27**

**13.02 24**

**13.04 28**

**13.06 36**

**13.08 38**

**13.1 42**

**13.12 42**

**13.14 51**

**13.16 51**

**13.18 53**

**13.2 44**

**13.22 37**

**13.24 42**

**13.26 36**

**13.28 35**

**13.3 28**

**13.32 28**

**13.34 24**

**13.36 29**

**13.38 18**

**13.4 14**

**13.42 18**

**13.44 16**

**13.46 14**

**13.48 12**

**13.5 15**

**13.52 14**

**13.54 15**

**13.56 13**

**13.58 10**

**13.6 11**

**13.62 10**

**13.64 12**

**13.66 13**

**13.68 12**

**13.7 16**

**13.72 16**

**13.74 18**

**13.76 12**

**13.78 14**

**13.8 11**

**13.82 14**

**13.84 13**

**13.86 15**

**13.88 8**

**13.9 8**

**13.92 10**

**13.94 8**

**13.96 7**

**13.98 10**

**14 15**

**14.02 18**

**14.04 17**

**14.06 14**

**14.08 13**

**14.1 12**

**14.12 12**

**14.14 17**

**14.16 16**

**14.18 13**

**14.2 18**

**14.22 17**

**14.24 17**

**14.26 15**

**14.28 16**

**14.3 17**

**14.32 12**

**14.34 13**

**14.36 15**

**14.38 13**

**14.4 12**

**14.42 16**

**14.44 11**

**14.46 8**

**14.48 13**

**14.5 9**

**14.52 6**

**14.54 5**

**14.56 5**

**14.58 10**

**14.6 9**

**14.62 12**

**14.64 9**

**14.66 11**

**14.68 7**

**14.7 9**

**14.72 11**

**14.74 6**

**14.76 9**

**14.78 6**

**14.8 7**

**14.82 8**

**14.84 10**

**14.86 9**

**14.88 7**

**14.9 12**

**14.92 9**

**14.94 6**

**14.96 9**

**14.98 9**

**15 9**

**15.02 10**

**15.04 6**

**15.06 6**

**15.08 10**

**15.1 11**

**15.12 12**

**15.14 9**

**15.16 9**

**15.18 7**

**15.2 7**

**15.22 9**

**15.24 6**

**15.26 6**

**15.28 7**

**15.3 9**

**15.32 10**

**15.34 12**

**15.36 8**

**15.38 7**

**15.4 6**

**15.42 5**

**15.44 8**

**15.46 8**

**15.48 9**

**15.5 6**

**15.52 8**

**15.54 8**

**15.56 7**

**15.58 7**

**15.6 6**

**15.62 5**

**15.64 5**

**15.66 7**

**15.68 9**

**15.7 10**

**15.72 9**

**15.74 8**

**15.76 7**

**15.78 8**

**15.8 9**

**15.82 10**

**15.84 8**

**15.86 5**

**15.88 8**

**15.9 8**

**15.92 12**

**15.94 12**

**15.96 9**

**15.98 7**

**16 5**

**16.02 8**

**16.04 8**

**16.06 6**

**16.08 11**

**16.1 10**

**16.12 8**

**16.14 11**

**16.16 12**

**16.18 9**

**16.2 9**

**16.22 9**

**16.24 8**

**16.26 10**

**16.28 7**

**16.3 6**

**16.32 10**

**16.34 11**

**16.36 8**

**16.38 6**

**16.4 9**

**16.42 9**

**16.44 7**

**16.46 13**

**16.48 13**

**16.5 7**

**16.52 9**

**16.54 6**

**16.56 7**

**16.58 6**

**16.6 4**

**16.62 7**

**16.64 8**

**16.66 4**

**16.68 4**

**16.7 5**

**16.72 8**

**16.74 7**

**16.76 9**

**16.78 9**

**16.8 8**

**16.82 9**

**16.84 10**

**16.86 8**

**16.88 7**

**16.9 8**

**16.92 5**

**16.94 5**

**16.96 6**

**16.98 6**

**17 5**

**17.02 7**

**17.04 5**

**17.06 4**

**17.08 5**

**17.1 6**

**17.12 11**

**17.14 13**

**17.16 9**

**17.18 7**

**17.2 5**

**17.22 6**

**17.24 9**

**17.26 7**

**17.28 7**

**17.3 7**

**17.32 11**

**17.34 9**

**17.36 8**

**17.38 9**

**17.4 9**

**17.42 8**

**17.44 8**

**17.46 11**

**17.48 10**

**17.5 9**

**17.52 11**

**17.54 7**

**17.56 8**

**17.58 9**

**17.6 8**

**17.62 6**

**17.64 6**

**17.66 6**

**17.68 6**

**17.7 9**

**17.72 10**

**17.74 9**

**17.76 8**

**17.78 7**

**17.8 8**

**17.82 7**

**17.84 11**

**17.86 9**

**17.88 7**

**17.9 6**

**17.92 7**

**17.94 9**

**17.96 9**

**17.98 10**

**18 10**

**18.02 8**

**18.04 9**

**18.06 7**

**18.08 6**

**18.1 7**

**18.12 12**

**18.14 7**

**18.16 7**

**18.18 7**

**18.2 5**

**18.22 10**

**18.24 11**

**18.26 11**

**18.28 7**

**18.3 8**

**18.32 8**

**18.34 9**

**18.36 9**

**18.38 7**

**18.4 6**

**18.42 10**

**18.44 10**

**18.46 11**

**18.48 12**

**18.5 11**

**18.52 13**

**18.54 12**

**18.56 8**

**18.58 8**

**18.6 12**

**18.62 10**

**18.64 12**

**18.66 11**

**18.68 9**

**18.7 7**

**18.72 9**

**18.74 10**

**18.76 10**

**18.78 13**

**18.8 12**

**18.82 13**

**18.84 15**

**18.86 16**

**18.88 14**

**18.9 14**

**18.92 17**

**18.94 20**

**18.96 20**

**18.98 16**

**19 19**

**19.02 26**

**19.04 25**

**19.06 24**

**19.08 23**

**19.1 21**

**19.12 24**

**19.14 25**

**19.16 22**

**19.18 17**

**19.2 15**

**19.22 14**

**19.24 13**

**19.26 14**

**19.28 10**

**19.3 11**

**19.32 16**

**19.34 15**

**19.36 12**

**19.38 13**

**19.4 10**

**19.42 9**

**19.44 8**

**19.46 7**

**19.48 6**

**19.5 8**

**19.52 9**

**19.54 9**

**19.56 10**

**19.58 14**

**19.6 12**

**19.62 7**

**19.64 10**

**19.66 12**

**19.68 11**

**19.7 14**

**19.72 13**

**19.74 12**

**19.76 13**

**19.78 9**

**19.8 6**

**19.82 5**

**19.84 6**

**19.86 8**

**19.88 11**

**19.9 8**

**19.92 8**

**19.94 11**

**19.96 12**

**19.98 10**

**20 10**

**20.02 10**

**20.04 11**

**20.06 11**

**20.08 10**

**20.1 9**

**20.12 12**

**20.14 14**

**20.16 9**

**20.18 6**

**20.2 6**

**20.22 7**

**20.24 7**

**20.26 7**

**20.28 8**

**20.3 12**

**20.32 14**

**20.34 11**

**20.36 7**

**20.38 4**

**20.4 6**

**20.42 7**

**20.44 8**

**20.46 10**

**20.48 10**

**20.5 12**

**20.52 15**

**20.54 12**

**20.56 10**

**20.58 9**

**20.6 8**

**20.62 8**

**20.64 7**

**20.66 7**

**20.68 6**

**20.7 7**

**20.72 10**

**20.74 11**

**20.76 7**

**20.78 7**

**20.8 6**

**20.82 8**

**20.84 8**

**20.86 7**

**20.88 7**

**20.9 8**

**20.92 10**

**20.94 8**

**20.96 7**

**20.98 7**

**21 5**

**21.02 4**

**21.04 5**

**21.06 9**

**21.08 10**

**21.1 10**

**21.12 9**

**21.14 8**

**21.16 6**

**21.18 9**

**21.2 7**

**21.22 9**

**21.24 9**

**21.26 13**

**21.28 13**

**21.3 5**

**21.32 7**

**21.34 8**

**21.36 5**

**21.38 3**

**21.4 6**

**21.42 9**

**21.44 11**

**21.46 10**

**21.48 7**

**21.5 9**

**21.52 11**

**21.54 10**

**21.56 6**

**21.58 7**

**21.6 7**

**21.62 8**

**21.64 8**

**21.66 7**

**21.68 9**

**21.7 9**

**21.72 9**

**21.74 8**

**21.76 10**

**21.78 12**

**21.8 10**

**21.82 10**

**21.84 14**

**21.86 7**

**21.88 6**

**21.9 7**

**21.92 8**

**21.94 8**

**21.96 10**

**21.98 14**

**22 7**

**22.02 9**

**22.04 11**

**22.06 9**

**22.08 8**

**22.1 8**

**22.12 9**

**22.14 9**

**22.16 5**

**22.18 4**

**22.2 8**

**22.22 10**

**22.24 9**

**22.26 11**

**22.28 11**

**22.3 14**

**22.32 14**

**22.34 11**

**22.36 9**

**22.38 12**

**22.4 10**

**22.42 10**

**22.44 9**

**22.46 13**

**22.48 8**

**22.5 7**

**22.52 11**

**22.54 15**

**22.56 11**

**22.58 14**

**22.6 12**

**22.62 9**

**22.64 10**

**22.66 12**

**22.68 13**

**22.7 12**

**22.72 13**

**22.74 14**

**22.76 12**

**22.78 11**

**22.8 13**

**22.82 17**

**22.84 17**

**22.86 14**

**22.88 16**

**22.9 15**

**22.92 15**

**22.94 17**

**22.96 19**

**22.98 16**

**23 21**

**23.02 26**

**23.04 31**

**23.06 32**

**23.08 35**

**23.1 40**

**23.12 37**

**23.14 43**

**23.16 43**

**23.18 48**

**23.2 54**

**23.22 66**

**23.24 70**

**23.26 75**

**23.28 72**

**23.3 68**

**23.32 73**

**23.34 73**

**23.36 69**

**23.38 76**

**23.4 87**

**23.42 84**

**23.44 66**

**23.46 57**

**23.48 46**

**23.5 42**

**23.52 43**

**23.54 50**

**23.56 28**

**23.58 31**

**23.6 35**

**23.62 20**

**23.64 20**

**23.66 23**

**23.68 21**

**23.7 21**

**23.72 14**

**23.74 22**

**23.76 20**

**23.78 17**

**23.8 17**

**23.82 8**

**23.84 13**

**23.86 18**

**23.88 20**

**23.9 19**

**23.92 13**

**23.94 13**

**23.96 16**

**23.98 14**

**24 14**

**24.02 9**

**24.04 13**

**24.06 7**

**24.08 9**

**24.1 10**

**24.12 14**

**24.14 16**

**24.16 13**

**24.18 12**

**24.2 14**

**24.22 10**

**24.24 11**

**24.26 13**

**24.28 7**

**24.3 9**

**24.32 11**

**24.34 11**

**24.36 12**

**24.38 6**

**24.4 17**

**24.42 13**

**24.44 10**

**24.46 10**

**24.48 12**

**24.5 10**

**24.52 11**

**24.54 19**

**24.56 10**

**24.58 12**

**24.6 10**

**24.62 10**

**24.64 14**

**24.66 12**

**24.68 14**

**24.7 15**

**24.72 9**

**24.74 13**

**24.76 13**

**24.78 15**

**24.8 17**

**24.82 18**

**24.84 13**

**24.86 10**

**24.88 18**

**24.9 20**

**24.92 16**

**24.94 8**

**24.96 10**

**24.98 18**

**25 20**

**25.02 16**

**25.04 19**

**25.06 20**

**25.08 22**

**25.1 18**

**25.12 20**

**25.14 27**

**25.16 21**

**25.18 23**

**25.2 28**

**25.22 30**

**25.24 27**

**25.26 39**

**25.28 35**

**25.3 42**

**25.32 46**

**25.34 54**

**25.36 54**

**25.38 43**

**25.4 54**

**25.42 73**

**25.44 58**

**25.46 72**

**25.48 51**

**25.5 49**

**25.52 67**

**25.54 48**

**25.56 61**

**25.58 50**

**25.6 47**

**25.62 40**

**25.64 38**

**25.66 50**

**25.68 43**

**25.7 45**

**25.72 33**

**25.74 27**

**25.76 33**

**25.78 41**

**25.8 45**

**25.82 32**

**25.84 30**

**25.86 28**

**25.88 30**

**25.9 46**

**25.92 43**

**25.94 44**

**25.96 45**

**25.98 40**

**26 55**

**26.02 55**

**26.04 56**

**26.06 67**

**26.08 71**

**26.1 75**

**26.12 94**

**26.14 84**

**26.16 98**

**26.18 132**

**26.2 151**

**26.22 160**

**26.24 152**

**26.26 193**

**26.28 210**

**26.3 258**

**26.32 319**

**26.34 345**

**26.36 398**

**26.38 434**

**26.4 511**

**26.42 547**

**26.44 555**

**26.46 623**

**26.48 635**

**26.5 626**

**26.52 591**

**26.54 550**

**26.56 528**

**26.58 506**

**26.6 539**

**26.62 512**

**26.64 451**

**26.66 398**

**26.68 359**

**26.7 314**

**26.72 305**

**26.74 215**

**26.76 214**

**26.78 168**

**26.8 174**

**26.82 108**

**26.84 127**

**26.86 103**

**26.88 107**

**26.9 84**

**26.92 79**

**26.94 73**

**26.96 80**

**26.98 87**

**27 95**

**27.02 83**

**27.04 114**

**27.06 102**

**27.08 109**

**27.1 131**

**27.12 144**

**27.14 138**

**27.16 125**

**27.18 119**

**27.2 134**

**27.22 125**

**27.24 129**

**27.26 128**

**27.28 113**

**27.3 116**

**27.32 111**

**27.34 125**

**27.36 113**

**27.38 107**

**27.4 99**

**27.42 86**

**27.44 85**

**27.46 86**

**27.48 81**

**27.5 79**

**27.52 75**

**27.54 68**

**27.56 61**

**27.58 61**

**27.6 69**

**27.62 63**

**27.64 62**

**27.66 53**

**27.68 39**

**27.7 33**

**27.72 41**

**27.74 39**

**27.76 41**

**27.78 32**

**27.8 45**

**27.82 30**

**27.84 32**

**27.86 25**

**27.88 25**

**27.9 26**

**27.92 20**

**27.94 28**

**27.96 22**

**27.98 18**

**28 20**

**28.02 20**

**28.04 26**

**28.06 22**

**28.08 29**

**28.1 24**

**28.12 35**

**28.14 30**

**28.16 28**

**28.18 39**

**28.2 39**

**28.22 48**

**28.24 47**

**28.26 64**

**28.28 63**

**28.3 62**

**28.32 71**

**28.34 88**

**28.36 82**

**28.38 86**

**28.4 77**

**28.42 68**

**28.44 79**

**28.46 88**

**28.48 98**

**28.5 87**

**28.52 81**

**28.54 79**

**28.56 66**

**28.58 75**

**28.6 65**

**28.62 57**

**28.64 43**

**28.66 42**

**28.68 45**

**28.7 44**

**28.72 32**

**28.74 27**

**28.76 29**

**28.78 25**

**28.8 22**

**28.82 22**

**28.84 20**

**28.86 14**

**28.88 17**

**28.9 20**

**28.92 22**

**28.94 18**

**28.96 20**

**28.98 16**

**29 18**

**29.02 16**

**29.04 14**

**29.06 16**

**29.08 19**

**29.1 19**

**29.12 17**

**29.14 13**

**29.16 10**

**29.18 12**

**29.2 12**

**29.22 15**

**29.24 17**

**29.26 10**

**29.28 11**

**29.3 16**

**29.32 14**

**29.34 9**

**29.36 9**

**29.38 15**

**29.4 10**

**29.42 9**

**29.44 11**

**29.46 12**

**29.48 13**

**29.5 16**

**29.52 14**

**29.54 13**

**29.56 7**

**29.58 7**

**29.6 7**

**29.62 8**

**29.64 9**

**29.66 15**

**29.68 9**

**29.7 10**

**29.72 12**

**29.74 10**

**29.76 9**

**29.78 8**

**29.8 10**

**29.82 9**

**29.84 7**

**29.86 11**

**29.88 8**

**29.9 8**

**29.92 10**

**29.94 11**

**29.96 8**

**29.98 7**

**30 11**

**30.02 7**

**30.04 11**

**30.06 10**

**30.08 9**

**30.1 13**

**30.12 9**

**30.14 8**

**30.16 16**

**30.18 16**

**30.2 13**

**30.22 10**

**30.24 12**

**30.26 11**

**30.28 12**

**30.3 10**

**30.32 7**

**30.34 9**

**30.36 9**

**30.38 7**

**30.4 10**

**30.42 8**

**30.44 6**

**30.46 10**

**30.48 8**

**30.5 10**

**30.52 8**

**30.54 6**

**30.56 6**

**30.58 5**

**30.6 11**

**30.62 10**

**30.64 10**

**30.66 9**

**30.68 6**

**30.7 11**

**30.72 10**

**30.74 6**

**30.76 7**

**30.78 7**

**30.8 9**

**30.82 12**

**30.84 8**

**30.86 10**

**30.88 11**

**30.9 9**

**30.92 10**

**30.94 6**

**30.96 9**

**30.98 8**

**31 6**

**31.02 7**

**31.04 8**

**31.06 10**

**31.08 12**

**31.1 14**

**31.12 11**

**31.14 7**

**31.16 7**

**31.18 10**

**31.2 8**

**31.22 7**

**31.24 9**

**31.26 9**

**31.28 9**

**31.3 8**

**31.32 5**

**31.34 7**

**31.36 8**

**31.38 9**

**31.4 9**

**31.42 9**

**31.44 8**

**31.46 7**

**31.48 10**

**31.5 11**

**31.52 10**

**31.54 9**

**31.56 12**

**31.58 15**

**31.6 12**

**31.62 15**

**31.64 15**

**31.66 14**

**31.68 13**

**31.7 16**

**31.72 21**

**31.74 20**

**31.76 20**

**31.78 17**

**31.8 19**

**31.82 23**

**31.84 26**

**31.86 25**

**31.88 27**

**31.9 31**

**31.92 36**

**31.94 43**

**31.96 43**

**31.98 51**

**32 66**

**32.02 74**

**32.04 75**

**32.06 78**

**32.08 74**

**32.1 75**

**32.12 81**

**32.14 85**

**32.16 79**

**32.18 61**

**32.2 65**

**32.22 68**

**32.24 58**

**32.26 51**

**32.28 55**

**32.3 55**

**32.32 44**

**32.34 37**

**32.36 32**

**32.38 30**

**32.4 28**

**32.42 32**

**32.44 31**

**32.46 26**

**32.48 24**

**32.5 24**

**32.52 22**

**32.54 19**

**32.56 25**

**32.58 27**

**32.6 23**

**32.62 24**

**32.64 23**

**32.66 21**

**32.68 22**

**32.7 25**

**32.72 26**

**32.74 28**

**32.76 31**

**32.78 36**

**32.8 38**

**32.82 31**

**32.84 29**

**32.86 26**

**32.88 30**

**32.9 28**

**32.92 30**

**32.94 31**

**32.96 29**

**32.98 44**

**33 44**

**33.02 37**

**33.04 39**

**33.06 38**

**33.08 25**

**33.1 21**

**33.12 24**

**33.14 24**

**33.16 29**

**33.18 27**

**33.2 24**

**33.22 22**

**33.24 20**

**33.26 23**

**33.28 24**

**33.3 20**

**33.32 28**

**33.34 24**

**33.36 17**

**33.38 20**

**33.4 24**

**33.42 30**

**33.44 29**

**33.46 35**

**33.48 43**

**33.5 46**

**33.52 49**

**33.54 51**

**33.56 54**

**33.58 70**

**33.6 82**

**33.62 91**

**33.64 102**

**33.66 92**

**33.68 96**

**33.7 90**

**33.72 84**

**33.74 79**

**33.76 84**

**33.78 87**

**33.8 79**

**33.82 65**

**33.84 60**

**33.86 62**

**33.88 57**

**33.9 51**

**33.92 48**

**33.94 46**

**33.96 32**

**33.98 32**

**34 28**

**34.02 21**

**34.04 27**

**34.06 24**

**34.08 18**

**34.1 18**

**34.12 15**

**34.14 21**

**34.16 19**

**34.18 14**

**34.2 15**

**34.22 16**

**34.24 10**

**34.26 9**

**34.28 12**

**34.3 14**

**34.32 8**

**34.34 13**

**34.36 12**

**34.38 16**

**34.4 17**

**34.42 14**

**34.44 14**

**34.46 11**

**34.48 11**

**34.5 12**

**34.52 12**

**34.54 9**

**34.56 10**

**34.58 10**

**34.6 8**

**34.62 11**

**34.64 8**

**34.66 4**

**34.68 6**

**34.7 8**

**34.72 7**

**34.74 6**

**34.76 10**

**34.78 10**

**34.8 10**

**34.82 8**

**34.84 8**

**34.86 7**

**34.88 5**

**34.9 5**

**34.92 7**

**34.94 7**

**34.96 9**

**34.98 11**

**35 10**

**35.02 5**

**35.04 5**

**35.06 6**

**35.08 11**

**35.1 7**

**35.12 7**

**35.14 8**

**35.16 10**

**35.18 10**

**35.2 9**

**35.22 9**

**35.24 15**

**35.26 11**

**35.28 11**

**35.3 10**

**35.32 13**

**35.34 14**

**35.36 8**

**35.38 5**

**35.4 10**

**35.42 10**

**35.44 8**

**35.46 10**

**35.48 10**

**35.5 13**

**35.52 14**

**35.54 10**

**35.56 7**

**35.58 6**

**35.6 9**

**35.62 7**

**35.64 5**

**35.66 7**

**35.68 6**

**35.7 8**

**35.72 3**

**35.74 7**

**35.76 6**

**35.78 10**

**35.8 11**

**35.82 16**

**35.84 10**

**35.86 8**

**35.88 10**

**35.9 10**

**35.92 11**

**35.94 12**

**35.96 9**

**35.98 10**

**36 5**

**36.02 6**

**36.04 4**

**36.06 4**

**36.08 5**

**36.1 9**

**36.12 8**

**36.14 6**

**36.16 9**

**36.18 14**

**36.2 19**

**36.22 9**

**36.24 11**

**36.26 11**

**36.28 10**

**36.3 14**

**36.32 18**

**36.34 10**

**36.36 18**

**36.38 14**

**36.4 8**

**36.42 20**

**36.44 23**

**36.46 15**

**36.48 14**

**36.5 23**

**36.52 27**

**36.54 25**

**36.56 26**

**36.58 25**

**36.6 27**

**36.62 34**

**36.64 38**

**36.66 37**

**36.68 34**

**36.7 43**

**36.72 48**

**36.74 56**

**36.76 46**

**36.78 51**

**36.8 45**

**36.82 47**

**36.84 39**

**36.86 34**

**36.88 39**

**36.9 37**

**36.92 33**

**36.94 35**

**36.96 28**

**36.98 19**

**37 28**

**37.02 17**

**37.04 24**

**37.06 27**

**37.08 20**

**37.1 17**

**37.12 14**

**37.14 12**

**37.16 11**

**37.18 12**

**37.2 12**

**37.22 11**

**37.24 11**

**37.26 20**

**37.28 18**

**37.3 17**

**37.32 13**

**37.34 10**

**37.36 7**

**37.38 9**

**37.4 5**

**37.42 14**

**37.44 16**

**37.46 15**

**37.48 16**

**37.5 11**

**37.52 11**

**37.54 10**

**37.56 12**

**37.58 10**

**37.6 7**

**37.62 7**

**37.64 8**

**37.66 11**

**37.68 7**

**37.7 6**

**37.72 9**

**37.74 13**

**37.76 9**

**37.78 8**

**37.8 14**

**37.82 5**

**37.84 5**

**37.86 11**

**37.88 12**

**37.9 8**

**37.92 12**

**37.94 4**

**37.96 13**

**37.98 11**

**38 13**

**38.02 14**

**38.04 5**

**38.06 8**

**38.08 5**

**38.1 7**

**38.12 8**

**38.14 9**

**38.16 7**

**38.18 8**

**38.2 9**

**38.22 10**

**38.24 17**

**38.26 6**

**38.28 8**

**38.3 7**

**38.32 7**

**38.34 12**

**38.36 15**

**38.38 6**

**38.4 5**

**38.42 17**

**38.44 9**

**38.46 14**

**38.48 16**

**38.5 14**

**38.52 11**

**38.54 16**

**38.56 12**

**38.58 19**

**38.6 15**

**38.62 34**

**38.64 29**

**38.66 23**

**38.68 32**

**38.7 38**

**38.72 43**

**38.74 29**

**38.76 43**

**38.78 40**

**38.8 46**

**38.82 52**

**38.84 54**

**38.86 56**

**38.88 43**

**38.9 46**

**38.92 38**

**38.94 42**

**38.96 46**

**38.98 36**

**39 48**

**39.02 46**

**39.04 46**

**39.06 21**

**39.08 22**

**39.1 27**

**39.12 26**

**39.14 23**

**39.16 19**

**39.18 25**

**39.2 23**

**39.22 12**

**39.24 8**

**39.26 14**

**39.28 22**

**39.3 15**

**39.32 15**

**39.34 14**

**39.36 12**

**39.38 14**

**39.4 6**

**39.42 9**

**39.44 15**

**39.46 11**

**39.48 13**

**39.5 6**

**39.52 14**

**39.54 11**

**39.56 10**

**39.58 5**

**39.6 5**

**39.62 12**

**39.64 6**

**39.66 8**

**39.68 11**

**39.7 19**

**39.72 16**

**39.74 9**

**39.76 14**

**39.78 18**

**39.8 17**

**39.82 11**

**39.84 12**

**39.86 19**

**39.88 14**

**39.9 14**

**39.92 19**

**39.94 18**

**39.96 26**

**39.98 27**

**40 29**

**40.02 29**

**40.04 27**

**40.06 32**

**40.08 38**

**40.1 47**

**40.12 43**

**40.14 63**

**40.16 58**

**40.18 51**

**40.2 59**

**40.22 54**

**40.24 59**

**40.26 52**

**40.28 53**

**40.3 57**

**40.32 50**

**40.34 55**

**40.36 51**

**40.38 44**

**40.4 44**

**40.42 33**

**40.44 42**

**40.46 26**

**40.48 23**

**40.5 22**

**40.52 21**

**40.54 19**

**40.56 16**

**40.58 23**

**40.6 20**

**40.62 16**

**40.64 26**

**40.66 17**

**40.68 17**

**40.7 10**

**40.72 13**

**40.74 18**

**40.76 10**

**40.78 11**

**40.8 14**

**40.82 8**

**40.84 14**

**40.86 13**

**40.88 10**

**40.9 9**

**40.92 8**

**40.94 11**

**40.96 18**

**40.98 15**

**41 16**

**41.02 12**

**41.04 13**

**41.06 10**

**41.08 8**

**41.1 9**

**41.12 9**

**41.14 13**

**41.16 10**

**41.18 7**

**41.2 11**

**41.22 17**

**41.24 19**

**41.26 15**

**41.28 16**

**41.3 17**

**41.32 17**

**41.34 10**

**41.36 15**

**41.38 15**

**41.4 19**

**41.42 18**

**41.44 26**

**41.46 25**

**41.48 24**

**41.5 33**

**41.52 18**

**41.54 26**

**41.56 36**

**41.58 40**

**41.6 34**

**41.62 30**

**41.64 33**

**41.66 38**

**41.68 23**

**41.7 24**

**41.72 27**

**41.74 25**

**41.76 22**

**41.78 24**

**41.8 28**

**41.82 25**

**41.84 21**

**41.86 23**

**41.88 24**

**41.9 19**

**41.92 16**

**41.94 19**

**41.96 22**

**41.98 16**

**42 22**

**42.02 11**

**42.04 9**

**42.06 4**

**42.08 14**

**42.1 14**

**42.12 9**

**42.14 13**

**42.16 10**

**42.18 9**

**42.2 12**

**42.22 14**

**42.24 9**

**42.26 13**

**42.28 12**

**42.3 16**

**42.32 13**

**42.34 8**

**42.36 11**

**42.38 13**

**42.4 18**

**42.42 11**

**42.44 17**

**42.46 22**

**42.48 15**

**42.5 14**

**42.52 18**

**42.54 18**

**42.56 17**

**42.58 13**

**42.6 18**

**42.62 17**

**42.64 18**

**42.66 16**

**42.68 14**

**42.7 12**

**42.72 12**

**42.74 10**

**42.76 9**

**42.78 15**

**42.8 14**

**42.82 16**

**42.84 13**

**42.86 17**

**42.88 19**

**42.9 16**

**42.92 16**

**42.94 16**

**42.96 12**

**42.98 18**

**43 18**

**43.02 17**

**43.04 19**

**43.06 18**

**43.08 19**

**43.1 22**

**43.12 29**

**43.14 18**

**43.16 17**

**43.18 26**

**43.2 31**

**43.22 30**

**43.24 27**

**43.26 29**

**43.28 23**

**43.3 34**

**43.32 40**

**43.34 39**

**43.36 31**

**43.38 28**

**43.4 28**

**43.42 38**

**43.44 32**

**43.46 29**

**43.48 39**

**43.5 44**

**43.52 27**

**43.54 29**

**43.56 36**

**43.58 38**

**43.6 43**

**43.62 42**

**43.64 42**

**43.66 45**

**43.68 48**

**43.7 38**

**43.72 44**

**43.74 42**

**43.76 43**

**43.78 32**

**43.8 31**

**43.82 42**

**43.84 32**

**43.86 27**

**43.88 29**

**43.9 26**

**43.92 25**

**43.94 23**

**43.96 26**

**43.98 18**

**44 13**

**44.02 16**

**44.04 17**

**44.06 22**

**44.08 16**

**44.1 16**

**44.12 13**

**44.14 13**

**44.16 16**

**44.18 17**

**44.2 14**

**44.22 11**

**44.24 7**

**44.26 11**

**44.28 11**

**44.3 14**

**44.32 15**

**44.34 8**

**44.36 11**

**44.38 9**

**44.4 10**

**44.42 10**

**44.44 6**

**44.46 8**

**44.48 11**

**44.5 6**

**44.52 7**

**44.54 9**

**44.56 11**

**44.58 10**

**44.6 8**

**44.62 5**

**44.64 10**

**44.66 13**

**44.68 11**

**44.7 12**

**44.72 13**

**44.74 9**

**44.76 12**

**44.78 19**

**44.8 22**

**44.82 15**

**44.84 19**

**44.86 22**

**44.88 22**

**44.9 17**

**44.92 22**

**44.94 23**

**44.96 26**

**44.98 22**

**45 25**

**45.02 25**

**45.04 33**

**45.06 27**

**45.08 27**

**45.1 30**

**45.12 33**

**45.14 23**

**45.16 26**

**45.18 30**

**45.2 29**

**45.22 28**

**45.24 31**

**45.26 30**

**45.28 31**

**45.3 35**

**45.32 31**

**45.34 27**

**45.36 24**

**45.38 24**

**45.4 34**

**45.42 25**

**45.44 24**

**45.46 21**

**45.48 23**

**45.5 18**

**45.52 13**

**45.54 17**

**45.56 19**

**45.58 14**

**45.6 18**

**45.62 18**

**45.64 13**

**45.66 12**

**45.68 18**

**45.7 23**

**45.72 19**

**45.74 14**

**45.76 13**

**45.78 10**

**45.8 17**

**45.82 14**

**45.84 17**

**45.86 11**

**45.88 7**

**45.9 8**

**45.92 10**

**45.94 16**

**45.96 16**

**45.98 10**

**46 14**

**46.02 12**

**46.04 6**

**46.06 13**

**46.08 15**

**46.1 16**

**46.12 14**

**46.14 16**

**46.16 24**

**46.18 20**

**46.2 16**

**46.22 18**

**46.24 16**

**46.26 13**

**46.28 24**

**46.3 16**

**46.32 23**

**46.34 21**

**46.36 17**

**46.38 21**

**46.4 17**

**46.42 16**

**46.44 15**

**46.46 19**

**46.48 15**

**46.5 15**

**46.52 21**

**46.54 20**

**46.56 19**

**46.58 17**

**46.6 17**

**46.62 16**

**46.64 15**

**46.66 18**

**46.68 19**

**46.7 14**

**46.72 14**

**46.74 20**

**46.76 27**

**46.78 25**

**46.8 25**

**46.82 29**

**46.84 28**

**46.86 24**

**46.88 32**

**46.9 31**

**46.92 30**

**46.94 41**

**46.96 42**

**46.98 47**

**47 45**

**47.02 43**

**47.04 35**

**47.06 39**

**47.08 39**

**47.1 46**

**47.12 50**

**47.14 42**

**47.16 48**

**47.18 42**

**47.2 47**

**47.22 47**

**47.24 48**

**47.26 49**

**47.28 44**

**47.3 56**

**47.32 59**

**47.34 37**

**47.36 45**

**47.38 48**

**47.4 47**

**47.42 42**

**47.44 55**

**47.46 42**

**47.48 49**

**47.5 51**

**47.52 45**

**47.54 43**

**47.56 41**

**47.58 38**

**47.6 32**

**47.62 34**

**47.64 33**

**47.66 28**

**47.68 32**

**47.7 20**

**47.72 24**

**47.74 26**

**47.76 21**

**47.78 27**

**47.8 22**

**47.82 22**

**47.84 17**

**47.86 14**

**47.88 21**

**47.9 15**

**47.92 10**

**47.94 19**

**47.96 15**

**47.98 11**

**48 16**

**48.02 23**

**48.04 16**

**48.06 13**

**48.08 18**

**48.1 17**

**48.12 10**

**48.14 9**

**48.16 13**

**48.18 14**

**48.2 16**

**48.22 18**

**48.24 20**

**48.26 12**

**48.28 10**

**48.3 11**

**48.32 9**

**48.34 12**

**48.36 15**

**48.38 14**

**48.4 17**

**48.42 16**

**48.44 14**

**48.46 15**

**48.48 12**

**48.5 6**

**48.52 8**

**48.54 4**

**48.56 12**

**48.58 12**

**48.6 13**

**48.62 8**

**48.64 10**

**48.66 12**

**48.68 10**

**48.7 11**

**48.72 11**

**48.74 12**

**48.76 14**

**48.78 14**

**48.8 13**

**48.82 14**

**48.84 15**

**48.86 20**

**48.88 14**

**48.9 11**

**48.92 15**

**48.94 14**

**48.96 13**

**48.98 18**

**49 16**

**49.02 10**

**49.04 12**

**49.06 11**

**49.08 8**

**49.1 6**

**49.12 8**

**49.14 7**

**49.16 5**

**49.18 7**

**49.2 9**

**49.22 5**

**49.24 5**

**49.26 13**

**49.28 8**

**49.3 4**

**49.32 6**

**49.34 11**

**49.36 14**

**49.38 12**

**49.4 13**

**49.42 11**

**49.44 10**

**49.46 7**

**49.48 9**

**49.5 13**

**49.52 8**

**49.54 9**

**49.56 12**

**49.58 11**

**49.6 13**

**49.62 10**

**49.64 7**

**49.66 7**

**49.68 9**

**49.7 4**

**49.72 9**

**49.74 11**

**49.76 11**

**49.78 12**

**49.8 10**

**49.82 14**

**49.84 12**

**49.86 14**

**49.88 11**

**49.9 8**

**49.92 5**

**49.94 8**

**49.96 8**

**49.98 10**

**50 5**

**50.02 11**

**50.04 8**

**50.06 10**

**50.08 11**

**50.1 8**

**50.12 12**

**50.14 11**

**50.16 9**

**50.18 7**

**50.2 7**

**50.22 9**

**50.24 7**

**50.26 9**

**50.28 7**

**50.3 9**

**50.32 5**

**50.34 6**

**50.36 7**

**50.38 7**

**50.4 5**

**50.42 6**

**50.44 12**

**50.46 10**

**50.48 7**

**50.5 10**

**50.52 4**

**50.54 10**

**50.56 8**

**50.58 7**

**50.6 4**

**50.62 10**

**50.64 7**

**50.66 7**

**50.68 9**

**50.7 10**

**50.72 8**

**50.74 6**

**50.76 5**

**50.78 7**

**50.8 6**

**50.82 5**

**50.84 7**

**50.86 8**

**50.88 11**

**50.9 12**

**50.92 7**

**50.94 7**

**50.96 4**

**50.98 10**

**51 6**

**51.02 7**

**51.04 7**

**51.06 9**

**51.08 9**

**51.1 5**

**51.12 3**

**51.14 10**

**51.16 6**

**51.18 8**

**51.2 8**

**51.22 8**

**51.24 7**

**51.26 10**

**51.28 14**

**51.3 14**

**51.32 12**

**51.34 10**

**51.36 9**

**51.38 12**

**51.4 12**

**51.42 7**

**51.44 11**

**51.46 12**

**51.48 11**

**51.5 10**

**51.52 13**

**51.54 12**

**51.56 17**

**51.58 8**

**51.6 14**

**51.62 13**

**51.64 9**

**51.66 15**

**51.68 16**

**51.7 13**

**51.72 14**

**51.74 11**

**51.76 14**

**51.78 19**

**51.8 13**

**51.82 15**

**51.84 22**

**51.86 15**

**51.88 17**

**51.9 16**

**51.92 19**

**51.94 25**

**51.96 26**

**51.98 26**

**52 31**

**52.02 27**

**52.04 34**

**52.06 50**

**52.08 45**

**52.1 45**

**52.12 35**

**52.14 38**

**52.16 47**

**52.18 37**

**52.2 33**

**52.22 40**

**52.24 52**

**52.26 34**

**52.28 35**

**52.3 34**

**52.32 25**

**52.34 29**

**52.36 30**

**52.38 31**

**52.4 27**

**52.42 35**

**52.44 22**

**52.46 24**

**52.48 22**

**52.5 17**

**52.52 27**

**52.54 20**

**52.56 12**

**52.58 16**

**52.6 21**

**52.62 23**

**52.64 21**

**52.66 16**

**52.68 19**

**52.7 11**

**52.72 14**

**52.74 23**

**52.76 15**

**52.78 20**

**52.8 10**

**52.82 23**

**52.84 18**

**52.86 15**

**52.88 20**

**52.9 19**

**52.92 14**

**52.94 8**

**52.96 14**

**52.98 15**

**53 14**

**53.02 23**

**53.04 12**

**53.06 20**

**53.08 20**

**53.1 23**

**53.12 17**

**53.14 22**

**53.16 22**

**53.18 25**

**53.2 15**

**53.22 15**

**53.24 18**

**53.26 29**

**53.28 24**

**53.3 29**

**53.32 32**

**53.34 34**

**53.36 36**

**53.38 31**

**53.4 38**

**53.42 48**

**53.44 43**

**53.46 38**

**53.48 48**

**53.5 46**

**53.52 41**

**53.54 57**

**53.56 50**

**53.58 42**

**53.6 47**

**53.62 41**

**53.64 37**

**53.66 50**

**53.68 42**

**53.7 36**

**53.72 35**

**53.74 49**

**53.76 42**

**53.78 39**

**53.8 45**

**53.82 34**

**53.84 38**

**53.86 30**

**53.88 24**

**53.9 16**

**53.92 33**

**53.94 22**

**53.96 13**

**53.98 26**

**54 29**

**54.02 33**

**54.04 26**

**54.06 17**

**54.08 17**

**54.1 22**

**54.12 22**

**54.14 22**

**54.16 24**

**54.18 20**

**54.2 18**

**54.22 20**

**54.24 25**

**54.26 27**

**54.28 24**

**54.3 26**

**54.32 22**

**54.34 18**

**54.36 27**

**54.38 30**

**54.4 34**

**54.42 44**

**54.44 38**

**54.46 41**

**54.48 39**

**54.5 47**

**54.52 46**

**54.54 43**

**54.56 41**

**54.58 45**

**54.6 31**

**54.62 31**

**54.64 45**

**54.66 29**

**54.68 31**

**54.7 38**

**54.72 32**

**54.74 23**

**54.76 39**

**54.78 27**

**54.8 23**

**54.82 25**

**54.84 16**

**54.86 26**

**54.88 23**

**54.9 25**

**54.92 16**

**54.94 19**

**54.96 20**

**54.98 15**

**55 18**

**55.02 21**

**55.04 16**

**55.06 9**

**55.08 17**

**55.1 18**

**55.12 18**

**55.14 16**

**55.16 15**

**55.18 19**

**55.2 14**

**55.22 17**

**55.24 15**

**55.26 22**

**55.28 24**

**55.3 20**

**55.32 16**

**55.34 17**

**55.36 18**

**55.38 29**

**55.4 19**

**55.42 35**

**55.44 25**

**55.46 30**

**55.48 35**

**55.5 23**

**55.52 30**

**55.54 35**

**55.56 25**

**55.58 31**

**55.6 38**

**55.62 32**

**55.64 31**

**55.66 38**

**55.68 28**

**55.7 36**

**55.72 27**

**55.74 27**

**55.76 31**

**55.78 24**

**55.8 26**

**55.82 27**

**55.84 19**

**55.86 20**

**55.88 34**

**55.9 24**

**55.92 19**

**55.94 25**

**55.96 24**

**55.98 19**

**56 28**

**56.02 29**

**56.04 34**

**56.06 26**

**56.08 29**

**56.1 28**

**56.12 26**

**56.14 20**

**56.16 23**

**56.18 32**

**56.2 31**

**56.22 16**

**56.24 34**

**56.26 24**

**56.28 16**

**56.3 25**

**56.32 19**

**56.34 19**

**56.36 24**

**56.38 27**

**56.4 20**

**56.42 29**

**56.44 16**

**56.46 23**

**56.48 10**

**56.5 25**

**56.52 18**

**56.54 13**

**56.56 18**

**56.58 19**

**56.6 18**

**56.62 26**

**56.64 26**

**56.66 17**

**56.68 26**

**56.7 21**

**56.72 22**

**56.74 13**

**56.76 27**

**56.78 27**

**56.8 20**

**56.82 20**

**56.84 12**

**56.86 23**

**56.88 17**

**56.9 29**

**56.92 18**

**56.94 21**

**56.96 19**

**56.98 19**

**57 22**

**57.02 20**

**57.04 21**

**57.06 23**

**57.08 18**

**57.1 22**

**57.12 19**

**57.14 23**

**57.16 16**

**57.18 15**

**57.2 18**

**57.22 22**

**57.24 18**

**57.26 14**

**57.28 13**

**57.3 15**

**57.32 11**

**57.34 18**

**57.36 17**

**57.38 19**

**57.4 13**

**57.42 15**

**57.44 3**

**57.46 8**

**57.48 16**

**57.5 11**

**57.52 13**

**57.54 17**

**57.56 17**

**57.58 11**

**57.6 14**

**57.62 15**

**57.64 13**

**57.66 20**

**57.68 12**

**57.7 12**

**57.72 12**

**57.74 19**

**57.76 17**

**57.78 13**

**57.8 14**

**57.82 11**

**57.84 18**

**57.86 13**

**57.88 16**

**57.9 11**

**57.92 12**

**57.94 11**

**57.96 16**

**57.98 13**

**58 17**

**58.02 11**

**58.04 16**

**58.06 13**

**58.08 16**

**58.1 17**

**58.12 15**

**58.14 13**

**58.16 17**

**58.18 18**

**58.2 24**

**58.22 26**

**58.24 18**

**58.26 27**

**58.28 27**

**58.3 31**

**58.32 32**

**58.34 18**

**58.36 24**

**58.38 31**

**58.4 25**

**58.42 37**

**58.44 31**

**58.46 32**

**58.48 27**

**58.5 29**

**58.52 29**

**58.54 36**

**58.56 30**

**58.58 31**

**58.6 27**

**58.62 29**

**58.64 30**

**58.66 28**

**58.68 30**

**58.7 20**

**58.72 29**

**58.74 28**

**58.76 24**

**58.78 25**

**58.8 27**

**58.82 26**

**58.84 22**

**58.86 27**

**58.88 27**

**58.9 22**

**58.92 20**

**58.94 23**

**58.96 24**

**58.98 21**

**59 26**

**59.02 22**

**59.04 14**

**59.06 29**

**59.08 20**

**59.1 24**

**59.12 17**

**59.14 22**

**59.16 17**

**59.18 18**

**59.2 17**

**59.22 19**

**59.24 20**

**59.26 15**

**59.28 18**

**59.3 14**

**59.32 17**

**59.34 18**

**59.36 14**

**59.38 15**

**59.4 12**

**59.42 16**

**59.44 20**

**59.46 19**

**59.48 23**

**59.5 22**

**59.52 17**

**59.54 20**

**59.56 13**

**59.58 13**

**59.6 17**

**59.62 13**

**59.64 13**

**59.66 12**

**59.68 15**

**59.7 19**

**59.72 28**

**59.74 20**

**59.76 17**

**59.78 28**

**59.8 27**

**59.82 24**

**59.84 28**

**59.86 24**

**59.88 32**

**59.9 32**

**59.92 45**

**59.94 27**

**59.96 31**

**59.98 36**

**60 27**

**60.02 25**

**60.04 38**

**60.06 37**

**60.08 28**

**60.1 33**

**60.12 32**

**60.14 34**

**60.16 44**

**60.18 49**

**60.2 33**

**60.22 41**

**60.24 42**

**60.26 45**

**60.28 43**

**60.3 43**

**60.32 36**

**60.34 40**

**60.36 42**

**60.38 37**

**60.4 35**

**60.42 39**

**60.44 30**

**60.46 32**

**60.48 38**

**60.5 27**

**60.52 30**

**60.54 25**

**60.56 28**

**60.58 27**

**60.6 23**

**60.62 19**

**60.64 25**

**60.66 21**

**60.68 27**

**60.7 24**

**60.72 23**

**60.74 21**

**60.76 16**

**60.78 20**

**60.8 23**

**60.82 23**

**60.84 20**

**60.86 15**

**60.88 19**

**60.9 21**

**60.92 15**

**60.94 24**

**60.96 18**

**60.98 21**

**61 20**

**61.02 22**

**61.04 26**

**61.06 22**

**61.08 12**

**61.1 27**

**61.12 20**

**61.14 28**

**61.16 16**

**61.18 16**

**61.2 20**

**61.22 23**

**61.24 17**

**61.26 18**

**61.28 24**

**61.3 23**

**61.32 17**

**61.34 21**

**61.36 26**

**61.38 28**

**61.4 20**

**61.42 17**

**61.44 25**

**61.46 30**

**61.48 39**

**61.5 26**

**61.52 18**

**61.54 24**

**61.56 27**

**61.58 24**

**61.6 15**

**61.62 17**

**61.64 24**

**61.66 22**

**61.68 22**

**61.7 23**

**61.72 20**

**61.74 18**

**61.76 25**

**61.78 33**

**61.8 29**

**61.82 21**

**61.84 20**

**61.86 17**

**61.88 20**

**61.9 25**

**61.92 24**

**61.94 21**

**61.96 24**

**61.98 23**

**62 20**

**62.02 27**

**62.04 14**

**62.06 22**

**62.08 24**

**62.1 29**

**62.12 25**

**62.14 16**

**62.16 18**

**62.18 21**

**62.2 17**

**62.22 23**

**62.24 16**

**62.26 20**

**62.28 22**

**62.3 15**

**62.32 21**

**62.34 16**

**62.36 18**

**62.38 16**

**62.4 21**

**62.42 15**

**62.44 15**

**62.46 18**

**62.48 19**

**62.5 15**

**62.52 16**

**62.54 18**

**62.56 16**

**62.58 13**

**62.6 18**

**62.62 14**

**62.64 9**

**62.66 11**

**62.68 17**

**62.7 13**

**62.72 15**

**62.74 13**

**62.76 17**

**62.78 23**

**62.8 15**

**62.82 14**

**62.84 20**

**62.86 27**

**62.88 24**

**62.9 15**

**62.92 18**

**62.94 24**

**62.96 30**

**62.98 31**

**63 26**

**63.02 30**

**63.04 29**

**63.06 29**

**63.08 24**

**63.1 39**

**63.12 27**

**63.14 26**

**63.16 29**

**63.18 24**

**63.2 33**

**63.22 36**

**63.24 24**

**63.26 24**

**63.28 20**

**63.3 29**

**63.32 36**

**63.34 33**

**63.36 29**

**63.38 33**

**63.4 20**

**63.42 15**

**63.44 21**

**63.46 24**

**63.48 24**

**63.5 23**

**63.52 21**

**63.54 22**

**63.56 22**

**63.58 24**

**63.6 15**

**63.62 13**

**63.64 15**

**63.66 14**

**63.68 22**

**63.7 20**

**63.72 16**

**63.74 20**

**63.76 13**

**63.78 20**

**63.8 19**

**63.82 12**

**63.84 15**

**63.86 16**

**63.88 17**

**63.9 17**

**63.92 13**

**63.94 11**

**63.96 19**

**63.98 17**

**64 18**

**64.02 11**

**64.04 20**

**64.06 24**

**64.08 20**

**64.1 17**

**64.12 22**

**64.14 16**

**64.16 19**

**64.18 18**

**64.2 20**

**64.22 18**

**64.24 29**

**64.26 25**

**64.28 21**

**64.3 19**

**64.32 23**

**64.34 26**

**64.36 27**

**64.38 22**

**64.4 27**

**64.42 29**

**64.44 32**

**64.46 25**

**64.48 28**

**64.5 27**

**64.52 31**

**64.54 40**

**64.56 35**

**64.58 36**

**64.6 24**

**64.62 23**

**64.64 33**

**64.66 36**

**64.68 37**

**64.7 28**

**64.72 27**

**64.74 29**

**64.76 27**

**64.78 23**

**64.8 27**

**64.82 25**

**64.84 19**

**64.86 23**

**64.88 25**

**64.9 37**

**64.92 22**

**64.94 21**

**64.96 19**

**64.98 19**

**65 16**

**65.02 16**

**65.04 23**

**65.06 15**

**65.08 13**

**65.1 15**

**65.12 19**

**65.14 15**

**65.16 14**

**65.18 9**

**65.2 11**

**65.22 14**

**65.24 18**

**65.26 12**

**65.28 17**

**65.3 10**

**65.32 12**

**65.34 14**

**65.36 14**

**65.38 8**

**65.4 11**

**65.42 11**

**65.44 12**

**65.46 8**

**65.48 7**

**65.5 12**

**65.52 9**

**65.54 6**

**65.56 8**

**65.58 10**

**65.6 12**

**65.62 9**

**65.64 7**

**65.66 6**

**65.68 8**

**65.7 13**

**65.72 10**

**65.74 10**

**65.76 11**

**65.78 15**

**65.8 15**

**65.82 8**

**65.84 8**

**65.86 9**

**65.88 14**

**65.9 13**

**65.92 9**

**65.94 10**

**65.96 8**

**65.98 7**

**66 13**

**66.02 12**

**66.04 8**

**66.06 15**

**66.08 14**

**66.1 12**

**66.12 11**

**66.14 12**

**66.16 11**

**66.18 17**

**66.2 13**

**66.22 18**

**66.24 16**

**66.26 11**

**66.28 6**

**66.3 6**

**66.32 10**

**66.34 12**

**66.36 12**

**66.38 12**

**66.4 16**

**66.42 11**

**66.44 10**

**66.46 9**

**66.48 9**

**66.5 10**

**66.52 10**

**66.54 12**

**66.56 13**

**66.58 11**

**66.6 17**

**66.62 13**

**66.64 10**

**66.66 16**

**66.68 21**

**66.7 16**

**66.72 15**

**66.74 23**

**66.76 22**

**66.78 18**

**66.8 15**

**66.82 17**

**66.84 18**

**66.86 21**

**66.88 19**

**66.9 16**

**66.92 21**

**66.94 17**

**66.96 15**

**66.98 25**

**67 23**

**67.02 17**

**67.04 23**

**67.06 15**

**67.08 24**

**67.1 32**

**67.12 22**

**67.14 28**

**67.16 16**

**67.18 35**

**67.2 16**

**67.22 20**

**67.24 14**

**67.26 17**

**67.28 17**

**67.3 17**

**67.32 17**

**67.34 15**

**67.36 14**

**67.38 20**

**67.4 22**

**67.42 24**

**67.44 15**

**67.46 11**

**67.48 13**

**67.5 21**

**67.52 19**

**67.54 19**

**67.56 14**

**67.58 13**

**67.6 12**

**67.62 16**

**67.64 12**

**67.66 12**

**67.68 16**

**67.7 13**

**67.72 21**

**67.74 12**

**67.76 17**

**67.78 23**

**67.8 15**

**67.82 14**

**67.84 15**

**67.86 14**

**67.88 16**

**67.9 12**

**67.92 17**

**67.94 13**

**67.96 10**

**67.98 18**

**68 15**

**68.02 14**

**68.04 7**

**68.06 16**

**68.08 14**

**68.1 16**

**68.12 13**

**68.14 12**

**68.16 13**

**68.18 12**

**68.2 14**

**68.22 20**

**68.24 12**

**68.26 9**

**68.28 11**

**68.3 10**

**68.32 17**

**68.34 12**

**68.36 12**

**68.38 12**

**68.4 11**

**68.42 21**

**68.44 19**

**68.46 11**

**68.48 9**

**68.5 10**

**68.52 9**

**68.54 12**

**68.56 17**

**68.58 13**

**68.6 10**

**68.62 16**

**68.64 11**

**68.66 8**

**68.68 6**

**68.7 6**

**68.72 7**

**68.74 10**

**68.76 7**

**68.78 10**

**68.8 7**

**68.82 13**

**68.84 13**

**68.86 14**

**68.88 14**

**68.9 11**

**68.92 11**

**68.94 13**

**68.96 10**

**68.98 10**

**69 17**

**69.02 21**

**69.04 14**

**69.06 16**

**69.08 13**

**69.1 23**

**69.12 21**

**69.14 12**

**69.16 14**

**69.18 16**

**69.2 26**

**69.22 16**

**69.24 22**

**69.26 13**

**69.28 14**

**69.3 11**

**69.32 12**

**69.34 11**

**69.36 20**

**69.38 13**

**69.4 16**

**69.42 15**

**69.44 12**

**69.46 14**

**69.48 13**

**69.5 18**

**69.52 13**

**69.54 13**

**69.56 15**

**69.58 9**

**69.6 5**

**69.62 11**

**69.64 8**

**69.66 11**

**69.68 11**

**69.7 17**

**69.72 11**

**69.74 14**

**69.76 12**

**69.78 10**

**69.8 7**

**69.82 10**

**69.84 14**

**69.86 15**

**69.88 18**

**69.9 18**

**69.92 13**

**69.94 8**

**69.96 12**

**69.98 15**

**70 6**

**70.02 10**

**70.04 14**

**70.06 14**

**70.08 13**

**70.1 10**

**70.12 10**

**70.14 11**

**70.16 16**

**70.18 13**

**70.2 12**

**70.22 7**

**70.24 10**

**70.26 5**

**70.28 7**

**70.3 9**

**70.32 9**

**70.34 9**

**70.36 7**

**70.38 10**

**70.4 15**

**70.42 7**

**70.44 9**

**70.46 7**

**70.48 7**

**70.5 6**

**70.52 9**

**70.54 10**

**70.56 7**

**70.58 6**

**70.6 9**

**70.62 10**

**70.64 4**

**70.66 7**

**70.68 12**

**70.7 14**

**70.72 6**

**70.74 8**

**70.76 9**

**70.78 9**

**70.8 10**

**70.82 7**

**70.84 6**

**70.86 8**

**70.88 9**

**70.9 9**

**70.92 8**

**70.94 11**

**70.96 12**

**70.98 9**

**71 7**

**71.02 10**

**71.04 8**

**71.06 8**

**71.08 13**

**71.1 11**

**71.12 13**

**71.14 9**

**71.16 7**

**71.18 9**

**71.2 13**

**71.22 13**

**71.24 16**

**71.26 13**

**71.28 16**

**71.3 10**

**71.32 11**

**71.34 10**

**71.36 10**

**71.38 10**

**71.4 18**

**71.42 11**

**71.44 10**

**71.46 11**

**71.48 7**

**71.5 9**

**71.52 10**

**71.54 13**

**71.56 18**

**71.58 15**

**71.6 11**

**71.62 10**

**71.64 13**

**71.66 10**

**71.68 9**

**71.7 15**

**71.72 9**

**71.74 5**

**71.76 5**

**71.78 6**

**71.8 11**

**71.82 8**

**71.84 9**

**71.86 9**

**71.88 13**

**71.9 7**

**71.92 7**

**71.94 6**

**71.96 8**

**71.98 11**

**72 10**

**72.02 9**

**72.04 16**

**72.06 8**

**72.08 13**

**72.1 11**

**72.12 9**

**72.14 10**

**72.16 12**

**72.18 13**

**72.2 6**

**72.22 7**

**72.24 8**

**72.26 8**

**72.28 4**

**72.3 7**

**72.32 8**

**72.34 11**

**72.36 5**

**72.38 8**

**72.4 3**

**72.42 6**

**72.44 7**

**72.46 7**

**72.48 7**

**72.5 6**

**72.52 6**

**72.54 7**

**72.56 7**

**72.58 8**

**72.6 12**

**72.62 14**

**72.64 6**

**72.66 12**

**72.68 9**

**72.7 7**

**72.72 10**

**72.74 7**

**72.76 6**

**72.78 7**

**72.8 7**

**72.82 7**

**72.84 8**

**72.86 13**

**72.88 13**

**72.9 13**

**72.92 11**

**72.94 9**

**72.96 8**

**72.98 10**

**73 10**

**73.02 7**

**73.04 9**

**73.06 11**

**73.08 16**

**73.1 10**

**73.12 4**

**73.14 9**

**73.16 9**

**73.18 10**

**73.2 16**

**73.22 13**

**73.24 9**

**73.26 10**

**73.28 10**

**73.3 10**

**73.32 12**

**73.34 9**

**73.36 15**

**73.38 17**

**73.4 14**

**73.42 20**

**73.44 15**

**73.46 19**

**73.48 17**

**73.5 12**

**73.52 14**

**73.54 19**

**73.56 20**

**73.58 20**

**73.6 29**

**73.62 19**

**73.64 23**

**73.66 27**

**73.68 27**

**73.7 19**

**73.72 14**

**73.74 13**

**73.76 19**

**73.78 16**

**73.8 22**

**73.82 19**

**73.84 16**

**73.86 18**

**73.88 8**

**73.9 12**

**73.92 20**

**73.94 12**

**73.96 19**

**73.98 14**

**74 14**

**74.02 21**

**74.04 18**

**74.06 14**

**74.08 14**

**74.1 18**

**74.12 14**

**74.14 21**

**74.16 18**

**74.18 17**

**74.2 24**

**74.22 18**

**74.24 19**

**74.26 21**

**74.28 13**

**74.3 23**

**74.32 18**

**74.34 12**

**74.36 20**

**74.38 9**

**74.4 22**

**74.42 13**

**74.44 15**

**74.46 17**

**74.48 24**

**74.5 16**

**74.52 22**

**74.54 26**

**74.56 26**

**74.58 21**

**74.6 15**

**74.62 18**

**74.64 15**

**74.66 16**

**74.68 12**

**74.7 8**

**74.72 18**

**74.74 15**

**74.76 15**

**74.78 16**

**74.8 18**

**74.82 16**

**74.84 17**

**74.86 18**

**74.88 15**

**74.9 8**

**74.92 7**

**74.94 11**

**74.96 14**

**74.98 14**

**75 15**

**75.02 13**

**75.04 17**

**75.06 11**

**75.08 11**

**75.1 15**

**75.12 7**

**75.14 9**

**75.16 13**

**75.18 10**

**75.2 18**

**75.22 7**

**75.24 7**

**75.26 12**

**75.28 10**

**75.3 13**

**75.32 5**

**75.34 18**

**75.36 12**

**75.38 5**

**75.4 15**

**75.42 15**

**75.44 14**

**75.46 13**

**75.48 15**

**75.5 11**

**75.52 12**

**75.54 16**

**75.56 18**

**75.58 16**

**75.6 10**

**75.62 12**

**75.64 15**

**75.66 12**

**75.68 17**

**75.7 13**

**75.72 12**

**75.74 13**

**75.76 13**

**75.78 16**

**75.8 16**

**75.82 21**

**75.84 12**

**75.86 12**

**75.88 17**

**75.9 18**

**75.92 15**

**75.94 22**

**75.96 14**

**75.98 12**

**76 15**

**76.02 21**

**76.04 15**

**76.06 19**

**76.08 6**

**76.1 24**

**76.12 19**

**76.14 14**

**76.16 21**

**76.18 22**

**76.2 19**

**76.22 13**

**76.24 17**

**76.26 15**

**76.28 19**

**76.3 16**

**76.32 8**

**76.34 14**

**76.36 14**

**76.38 24**

**76.4 12**

**76.42 25**

**76.44 12**

**76.46 12**

**76.48 15**

**76.5 23**

**76.52 14**

**76.54 9**

**76.56 17**

**76.58 8**

**76.6 12**

**76.62 23**

**76.64 20**

**76.66 16**

**76.68 14**

**76.7 11**

**76.72 18**

**76.74 10**

**76.76 14**

**76.78 8**

**76.8 12**

**76.82 13**

**76.84 15**

**76.86 12**

**76.88 14**

**76.9 16**

**76.92 13**

**76.94 17**

**76.96 14**

**76.98 21**

**77 8**

**77.02 13**

**77.04 19**

**77.06 8**

**77.08 15**

**77.1 16**

**77.12 21**

**77.14 14**

**77.16 17**

**77.18 20**

**77.2 15**

**77.22 14**

**77.24 15**

**77.26 12**

**77.28 20**

**77.3 20**

**77.32 11**

**77.34 14**

**77.36 12**

**77.38 9**

**77.4 15**

**77.42 19**

**77.44 13**

**77.46 11**

**77.48 11**

**77.5 16**

**77.52 18**

**77.54 21**

**77.56 8**

**77.58 16**

**77.6 15**

**77.62 11**

**77.64 13**

**77.66 14**

**77.68 9**

**77.7 11**

**77.72 16**

**77.74 14**

**77.76 17**

**77.78 14**

**77.8 12**

**77.82 8**

**77.84 11**

**77.86 9**

**77.88 5**

**77.9 8**

**77.92 14**

**77.94 16**

**77.96 16**

**77.98 17**

**78 11**

**78.02 13**

**78.04 13**

**78.06 11**

**78.08 14**

**78.1 14**

**78.12 16**

**78.14 11**

**78.16 10**

**78.18 7**

**78.2 11**

**78.22 15**

**78.24 9**

**78.26 10**

**78.28 13**

**78.3 12**

**78.32 12**

**78.34 8**

**78.36 6**

**78.38 8**

**78.4 14**

**78.42 7**

**78.44 8**

**78.46 5**

**78.48 6**

**78.5 13**

**78.52 12**

**78.54 5**

**78.56 8**

**78.58 10**

**78.6 6**

**78.62 11**

**78.64 8**

**78.66 5**

**78.68 7**

**78.7 4**

**78.72 7**

**78.74 10**

**78.76 8**

**78.78 9**

**78.8 7**

**78.82 10**

**78.84 11**

**78.86 10**

**78.88 13**

**78.9 7**

**78.92 5**

**78.94 13**

**78.96 9**

**78.98 7**

**79 6**

**79.02 10**

**79.04 9**

**79.06 6**

**79.08 7**

**79.1 11**

**79.12 6**

**79.14 15**

**79.16 9**

**79.18 5**

**79.2 10**

**79.22 13**

**79.24 9**

**79.26 9**

**79.28 8**

**79.3 6**

**79.32 12**

**79.34 5**

**79.36 8**

**79.38 13**

**79.4 10**

**79.42 9**

**79.44 11**

**79.46 12**

**79.48 9**

**79.5 9**

**79.52 5**

**79.54 6**

**79.56 10**

**79.58 11**

**79.6 5**

**79.62 9**

**79.64 6**

**79.66 15**

**79.68 8**

**79.7 15**

**79.72 11**

**79.74 8**

**79.76 7**

**79.78 5**

**79.8 3**

**79.82 9**

**79.84 8**

**79.86 9**

**79.88 10**

**79.9 10**

**79.92 10**

**79.94 9**

**79.96 13**

**79.98 12**

**80 11**

**Ag/CoMoO4**

***RAS_DATA_START --**

***RAS_HEADER_START --**

***CORR_POS_DB_NAME "Si**

***CORR_POS_GONIO_RADIUS ""**

***CORR_POS_NODE_INDEX01 ""**

***CORR_POS_NODE_INDEX02 ""**

***CORR_POS_NODE_INDEX03 ""**

***CORR_POS_NODE_INDEX04 ""**

***CORR_POS_NODE_INDEX05 ""**

***CORR_POS_NODE_INDEX06 ""**

***CORR_POS_NODE_INDEX07 ""**

***CORR_POS_NODE_INDEX08 ""**

***CORR_POS_NODE_INDEX09 ""**

***CORR_POS_NODE_INDEX10 ""**

***CORR_POS_NODE_INDEX11 ""**

***CORR_POS_NODE_LABEL01 "Si(111)"**

***CORR_POS_NODE_LABEL02 "Si(220)"**

***CORR_POS_NODE_LABEL03 "Si(311)"**

***CORR_POS_NODE_LABEL04 "Si(400)"**

***CORR_POS_NODE_LABEL05 "Si(331)"**

***CORR_POS_NODE_LABEL06 "Si(422)"**

***CORR_POS_NODE_LABEL07 "Si(511)"**

***CORR_POS_NODE_LABEL08 "Si(440)"**

***CORR_POS_NODE_LABEL09 "Si(531)"**

***CORR_POS_NODE_LABEL10 "Si(620)"**

***CORR_POS_NODE_LABEL11 "Si(533)"**

***CORR_POS_NODE_POS_CALC01 "28.377"**

***CORR_POS_NODE_POS_CALC02 "47.2506"**

***CORR_POS_NODE_POS_CALC03 "56.0725"**

***CORR_POS_NODE_POS_CALC04 "69.0822"**

***CORR_POS_NODE_POS_CALC05 "76.3294"**

***CORR_POS_NODE_POS_CALC06 "87.9853"**

***CORR_POS_NODE_POS_CALC07 "94.9091"**

***CORR_POS_NODE_POS_CALC08 "106.668"**

***CORR_POS_NODE_POS_CALC09 "114.054"**

***CORR_POS_NODE_POS_CALC10 "127.511"**

***CORR_POS_NODE_POS_CALC11 "136.864"**

***CORR_POS_NODE_POS_MEAS01 "28.3931"**

***CORR_POS_NODE_POS_MEAS02 "47.2458"**

***CORR_POS_NODE_POS_MEAS03 "56.0729"**

***CORR_POS_NODE_POS_MEAS04 "69.0852"**

***CORR_POS_NODE_POS_MEAS05 "76.3288"**

***CORR_POS_NODE_POS_MEAS06 "87.9726"**

***CORR_POS_NODE_POS_MEAS07 "94.8953"**

***CORR_POS_NODE_POS_MEAS08 "106.657"**

***CORR_POS_NODE_POS_MEAS09 "114.038"**

***CORR_POS_NODE_POS_MEAS10 "127.496"**

***CORR_POS_NODE_POS_MEAS11 "136.855"**

***CORR_POS_OPT_ATTR "W@"**

***CORR_POS_PARAM_MU ""**

***CORR_POS_PARAM_PL ""**

***CORR_POS_SEMODEL_TYPE "0"**

***CORR_POS_STANDARD_TYPE ""**

***CORR_POS_VALUE_ANALYZER "None"**

***CORR_POS_VALUE_CMONO "None"**

***CORR_POS_VALUE_IMONO "None"**

***CORR_POS_VALUE_IS "1.250deg"**

***CORR_POS_VALUE_ISL "10.0mm"**

***CORR_POS_VALUE_ISOLLER "5.0deg"**

***CORR_POS_VALUE_RS1 "1.250deg"**

***CORR_POS_VALUE_RS2 "0.3mm"**

***CORR_POS_VALUE_RSOLLER "5.0deg"**

***CORR_POS_XG_FOCUS_L ""**

***CORR_POS_XG_WAVE_TYPE ""**

***CORR_POS_XG_WAVE_VALUE ""**

***DISP_FMT_X "%.2f"**

***DISP_FMT_Y "%.0f"**

***DISP_FULLSCALE "442.0000000000"**

***DISP_LINE_COLOR "255.0000000000"**

***DISP_LINE_STYLE "0.0000000000"**

***DISP_LINE_WIDTH "0.2500000000"**

***DISP_NOTE "Collected**

***DISP_OFFSET_Y "0.0000000000"**

***DISP_PEAKSEARCH_UNIT_Y "cps"**

***DISP_RANGE_BOTTOM "0.0000000000"**

***DISP_RANGE_LEFT "10.0000000000"**

***DISP_RANGE_LOGCUTOFF "0.0100000000"**

***DISP_RANGE_RIGHT "80.0000000000"**

***DISP_RANGE_TOP "442.0000000000"**

***DISP_SCALE_MODE_Y "0.0000000000"**

***DISP_TAB_NAME "Theta/2-Theta"**

***DISP_TITLE_Y "Intensity"**

***DISP_UNIT_Y "cps"**

***DISP_WINDOW_NAME "MSG_MACROENGINE"**

***FILE_COMMENT "**

***FILE_MD5 ""**

***FILE_MEMO "**

***FILE_OPERATOR "Administrator"**

***FILE_SAMPLE "CoMoO4**

***FILE_TYPE "RAS_RAW"**

***FILE_USERGROUP "System**

***FILE_VERSION "1.0000000000"**

***HW_ATTACHMENT_ID "ATT1025"**

***HW_ATTACHMENT_NAME "W¿ä|Standard**

***HW_COUNTER_ID-0 "CUT0021"**

***HW_COUNTER_ID-1 "CUT0020"**

***HW_COUNTER_ID-2 "CUT0020"**

***HW_COUNTER_ID-3 "CMC0021"**

***HW_COUNTER_NAME-0 "SC-70|SC-70"**

***HW_COUNTER_NAME-1 "None|None"**

***HW_COUNTER_NAME-2 "None|None"**

***HW_COUNTER_NAME-3 "oímN[^|Detector**

***HW_COUNTER_SELECT_NAME "SC-70|SC-70"**

***HW_ECP_HV "700.0000000000"**

***HW_ECP_HV_UNIT "V"**

***HW_GONIOMETER_ID "GON0070"**

***HW_GONIOMETER_NAME "MiniFlex**

***HW_GONIOMETER_RADIUS-0 "79.0"**

***HW_GONIOMETER_RADIUS-1 "91.5"**

***HW_GONIOMETER_RADIUS-2 "150.0"**

***HW_GONIOMETER_RADIUS-3 "0"**

***HW_GONIOMETER_RADIUS-4 "103.4"**

***HW_GONIOMETER_RADIUS-5 "150.0"**

***HW_GONIOMETER_RADIUS-6 "46.6"**

***HW_GONIOMETER_RADIUS-7 "160.0"**

***HW_I_OPT_ID-0 "ISL0021"**

***HW_I_OPT_ID-1 "ISO0021"**

***HW_I_OPT_NAME-0 "­UXbg|Divergence**

***HW_I_OPT_NAME-1 "üË½sXbg|Incident**

***HW_R_ATTENUATER_AUTOMODE "0"**

***HW_R_OPT_ID-0 "RSS0021"**

***HW_R_OPT_ID-1 "RSO0021"**

***HW_R_OPT_ID-2 "RRS0021"**

***HW_R_OPT_NAME-0 "UXbg|Scattering**

***HW_R_OPT_NAME-1 "óõ½sXbg|Receiving**

***HW_R_OPT_NAME-2 "óõXbg|Receiving**

***HW_SAMPLE_HOLDER_ID "SMP0000"**

***HW_SAMPLE_HOLDER_NAME "³µ|None"**

***HW_XG_CURRENT_MAX "15"**

***HW_XG_CURRENT_MIN "2"**

***HW_XG_CURRENT_RESOLUTION "1"**

***HW_XG_CURRENT_UNIT "mA"**

***HW_XG_FOCUS "1mm**

***HW_XG_FOCUS_TYPE "Normal"**

***HW_XG_ID ""**

***HW_XG_NAME "300W/600W**

***HW_XG_TARGET_ATOMIC_NUMBER "74.0000000000"**

***HW_XG_TARGET_NAME "W"**

***HW_XG_TYPE "Hermetic"**

***HW_XG_VOLTAGE_MAX "40"**

***HW_XG_VOLTAGE_MIN "20"**

***HW_XG_VOLTAGE_RESOLUTION "1"**

***HW_XG_VOLTAGE_UNIT "kV"**

***HW_XG_WATT_MAX "0.600"**

***HW_XG_WATT_UNIT "kW"**

***HW_XG_WAVE_LENGTH_ALPHA1 "0.209013"**

***HW_XG_WAVE_LENGTH_ALPHA2 "0.213831"**

***HW_XG_WAVE_LENGTH_BETA "0.184377"**

***MEAS_COND_AXIS_NAME-0 "Theta/2-Theta"**

***MEAS_COND_AXIS_NAME-1 "Soller(inc.)"**

***MEAS_COND_AXIS_NAME-2 "IHS"**

***MEAS_COND_AXIS_NAME-3 "DS"**

***MEAS_COND_AXIS_NAME-4 "SS"**

***MEAS_COND_AXIS_NAME-5 "Soller(rec.)"**

***MEAS_COND_AXIS_NAME-6 "RS"**

***MEAS_COND_AXIS_NAME-7 "Filter"**

***MEAS_COND_AXIS_NAME-8 "Monochromator"**

***MEAS_COND_AXIS_NAME-9 "HV"**

***MEAS_COND_AXIS_NAME-10 "PHA"**

***MEAS_COND_AXIS_NAME_INTERNAL-0 "TwoThetaTheta"**

***MEAS_COND_AXIS_NAME_INTERNAL-1 "IncidentSollerSlit"**

***MEAS_COND_AXIS_NAME_INTERNAL-2 "IncidentAxdSlit"**

***MEAS_COND_AXIS_NAME_INTERNAL-3 "IncidentSlitBox"**

***MEAS_COND_AXIS_NAME_INTERNAL-4 "ReceivingSlitBox1"**

***MEAS_COND_AXIS_NAME_INTERNAL-5 "ReceivingSollerSlit"**

***MEAS_COND_AXIS_NAME_INTERNAL-6 "ReceivingSlitBox2"**

***MEAS_COND_AXIS_NAME_INTERNAL-7 "Filter"**

***MEAS_COND_AXIS_NAME_INTERNAL-8 "CounterMonochromator"**

***MEAS_COND_AXIS_NAME_INTERNAL-9 "HV"**

***MEAS_COND_AXIS_NAME_INTERNAL-10 "PHA"**

***MEAS_COND_AXIS_NAME_MAGICNO-0 "268632064.0000000000"**

***MEAS_COND_AXIS_NAME_MAGICNO-1 "20971521.0000000000"**

***MEAS_COND_AXIS_NAME_MAGICNO-2 "22020097.0000000000"**

***MEAS_COND_AXIS_NAME_MAGICNO-3 "22020097.0000000000"**

***MEAS_COND_AXIS_NAME_MAGICNO-4 "23068673.0000000000"**

***MEAS_COND_AXIS_NAME_MAGICNO-5 "20971521.0000000000"**

***MEAS_COND_AXIS_NAME_MAGICNO-6 "23068673.0000000000"**

***MEAS_COND_AXIS_NAME_MAGICNO-7 "20971521.0000000000"**

***MEAS_COND_AXIS_NAME_MAGICNO-8 "20971521.0000000000"**

***MEAS_COND_AXIS_NAME_MAGICNO-9 "0.0000000000"**

***MEAS_COND_AXIS_NAME_MAGICNO-10 "0.0000000000"**

***MEAS_COND_AXIS_OFFSET-0 "0.0000000000"**

***MEAS_COND_AXIS_OFFSET-1 "-"**

***MEAS_COND_AXIS_OFFSET-2 "-"**

***MEAS_COND_AXIS_OFFSET-3 "-"**

***MEAS_COND_AXIS_OFFSET-4 "-"**

***MEAS_COND_AXIS_OFFSET-5 "-"**

***MEAS_COND_AXIS_OFFSET-6 "-"**

***MEAS_COND_AXIS_OFFSET-7 "-"**

***MEAS_COND_AXIS_OFFSET-8 "-"**

***MEAS_COND_AXIS_OFFSET-9 "-"**

***MEAS_COND_AXIS_OFFSET-10 "-"**

***MEAS_COND_AXIS_POSITION-0 "10.0000000000"**

***MEAS_COND_AXIS_POSITION-1 "5.0deg"**

***MEAS_COND_AXIS_POSITION-2 "10.0mm"**

***MEAS_COND_AXIS_POSITION-3 "1.250deg"**

***MEAS_COND_AXIS_POSITION-4 "13.0mm"**

***MEAS_COND_AXIS_POSITION-5 "5.0deg"**

***MEAS_COND_AXIS_POSITION-6 "13.0mm"**

***MEAS_COND_AXIS_POSITION-7 "None"**

***MEAS_COND_AXIS_POSITION-8 "None"**

***MEAS_COND_AXIS_POSITION-9 "700.0000000000"**

***MEAS_COND_AXIS_POSITION-10 "500.0000000000"**

***MEAS_COND_AXIS_UNIT-0 "deg"**

***MEAS_COND_AXIS_UNIT-1 ""**

***MEAS_COND_AXIS_UNIT-2 ""**

***MEAS_COND_AXIS_UNIT-3 ""**

***MEAS_COND_AXIS_UNIT-4 ""**

***MEAS_COND_AXIS_UNIT-5 ""**

***MEAS_COND_AXIS_UNIT-6 ""**

***MEAS_COND_AXIS_UNIT-7 ""**

***MEAS_COND_AXIS_UNIT-8 ""**

***MEAS_COND_AXIS_UNIT-9 "V"**

***MEAS_COND_AXIS_UNIT-10 "mV"**

***MEAS_COND_COUNTER_CNTLOSS_CORR "0.0000000000"**

***MEAS_COND_COUNTER_PHABASE "500.0000000000"**

***MEAS_COND_COUNTER_PHAWINDOW "1000.0000000000"**

***MEAS_COND_COUNTER_PHA_UNIT "mV"**

***MEAS_COND_COUNTER_TAUDIFF "0.2500000000"**

***MEAS_COND_COUNTER_TAUINT "0.7500000000"**

***MEAS_COND_IRRADIATION_MODE "OFF"**

***MEAS_COND_IRRADIATION_WIDTH ""**

***MEAS_COND_OPT_ATTR "**

***MEAS_COND_OPT_NAME ""**

***MEAS_COND_UNSYMMETRIC_MODE "OFF"**

***MEAS_COND_XG_CURRENT "15.0000000000"**

***MEAS_COND_XG_VOLTAGE "40.0000000000"**

***MEAS_COND_XG_WAVE_TYPE "Ka"**

***MEAS_DATA_COUNT "3501.0000000000"**

***MEAS_SCAN_AXIS_X "Theta/2-Theta"**

***MEAS_SCAN_AXIS_X_INTERNAL "TwoThetaTheta"**

***MEAS_SCAN_END_TIME "02/17/23**

***MEAS_SCAN_MODE "CONTINUOUS"**

***MEAS_SCAN_MONITOR "0"**

***MEAS_SCAN_RESOLUTION_X "0.0025"**

***MEAS_SCAN_SPEED "1.0000"**

***MEAS_SCAN_SPEED_UNIT "deg/min"**

***MEAS_SCAN_START "10.0000000000"**

***MEAS_SCAN_START_TIME "02/17/23**

***MEAS_SCAN_STEP "0.0200000000"**

***MEAS_SCAN_STOP "80.0000000000"**

***MEAS_SCAN_UNIT_X "deg"**

***MEAS_SCAN_UNIT_Y "counts"**

***SLIT_SYSTEM "0"**

***RAS_HEADER_END --**

***RAS_INT_START --**

**10 7**

**10.02 9**

**10.04 6**

**10.06 11**

**10.08 8**

**10.1 6**

**10.12 11**

**10.14 14**

**10.16 9**

**10.18 8**

**10.2 11**

**10.22 13**

**10.24 10**

**10.26 7**

**10.28 8**

**10.3 8**

**10.32 11**

**10.34 11**

**10.36 8**

**10.38 10**

**10.4 8**

**10.42 11**

**10.44 9**

**10.46 10**

**10.48 7**

**10.5 3**

**10.52 10**

**10.54 13**

**10.56 9**

**10.58 8**

**10.6 9**

**10.62 13**

**10.64 9**

**10.66 9**

**10.68 6**

**10.7 8**

**10.72 11**

**10.74 9**

**10.76 6**

**10.78 11**

**10.8 7**

**10.82 9**

**10.84 14**

**10.86 10**

**10.88 7**

**10.9 9**

**10.92 7**

**10.94 7**

**10.96 11**

**10.98 10**

**11 13**

**11.02 15**

**11.04 7**

**11.06 8**

**11.08 5**

**11.1 11**

**11.12 8**

**11.14 7**

**11.16 7**

**11.18 6**

**11.2 15**

**11.22 9**

**11.24 9**

**11.26 11**

**11.28 7**

**11.3 6**

**11.32 6**

**11.34 5**

**11.36 7**

**11.38 10**

**11.4 4**

**11.42 7**

**11.44 8**

**11.46 5**

**11.48 6**

**11.5 8**

**11.52 7**

**11.54 9**

**11.56 9**

**11.58 3**

**11.6 9**

**11.62 6**

**11.64 9**

**11.66 9**

**11.68 10**

**11.7 5**

**11.72 8**

**11.74 7**

**11.76 18**

**11.78 12**

**11.8 8**

**11.82 10**

**11.84 8**

**11.86 12**

**11.88 12**

**11.9 5**

**11.92 14**

**11.94 13**

**11.96 9**

**11.98 8**

**12 6**

**12.02 13**

**12.04 10**

**12.06 10**

**12.08 7**

**12.1 8**

**12.12 8**

**12.14 5**

**12.16 8**

**12.18 9**

**12.2 7**

**12.22 9**

**12.24 9**

**12.26 7**

**12.28 8**

**12.3 8**

**12.32 10**

**12.34 7**

**12.36 5**

**12.38 14**

**12.4 7**

**12.42 12**

**12.44 11**

**12.46 11**

**12.48 11**

**12.5 6**

**12.52 6**

**12.54 8**

**12.56 11**

**12.58 15**

**12.6 11**

**12.62 11**

**12.64 13**

**12.66 11**

**12.68 12**

**12.7 8**

**12.72 9**

**12.74 10**

**12.76 8**

**12.78 16**

**12.8 11**

**12.82 12**

**12.84 10**

**12.86 13**

**12.88 15**

**12.9 13**

**12.92 18**

**12.94 17**

**12.96 23**

**12.98 21**

**13 15**

**13.02 25**

**13.04 26**

**13.06 22**

**13.08 28**

**13.1 28**

**13.12 26**

**13.14 24**

**13.16 34**

**13.18 23**

**13.2 31**

**13.22 30**

**13.24 25**

**13.26 26**

**13.28 18**

**13.3 26**

**13.32 24**

**13.34 14**

**13.36 14**

**13.38 17**

**13.4 15**

**13.42 16**

**13.44 11**

**13.46 15**

**13.48 16**

**13.5 16**

**13.52 10**

**13.54 7**

**13.56 9**

**13.58 8**

**13.6 12**

**13.62 12**

**13.64 14**

**13.66 8**

**13.68 12**

**13.7 10**

**13.72 13**

**13.74 19**

**13.76 14**

**13.78 11**

**13.8 14**

**13.82 11**

**13.84 14**

**13.86 21**

**13.88 21**

**13.9 23**

**13.92 25**

**13.94 28**

**13.96 31**

**13.98 32**

**14 43**

**14.02 39**

**14.04 34**

**14.06 52**

**14.08 44**

**14.1 35**

**14.12 43**

**14.14 30**

**14.16 36**

**14.18 37**

**14.2 31**

**14.22 37**

**14.24 34**

**14.26 38**

**14.28 27**

**14.3 30**

**14.32 30**

**14.34 34**

**14.36 24**

**14.38 15**

**14.4 17**

**14.42 13**

**14.44 11**

**14.46 11**

**14.48 16**

**14.5 9**

**14.52 10**

**14.54 10**

**14.56 12**

**14.58 15**

**14.6 15**

**14.62 15**

**14.64 12**

**14.66 11**

**14.68 9**

**14.7 11**

**14.72 9**

**14.74 12**

**14.76 11**

**14.78 6**

**14.8 8**

**14.82 11**

**14.84 8**

**14.86 9**

**14.88 11**

**14.9 15**

**14.92 8**

**14.94 9**

**14.96 6**

**14.98 10**

**15 9**

**15.02 8**

**15.04 8**

**15.06 6**

**15.08 9**

**15.1 8**

**15.12 6**

**15.14 10**

**15.16 9**

**15.18 6**

**15.2 10**

**15.22 8**

**15.24 7**

**15.26 8**

**15.28 9**

**15.3 9**

**15.32 8**

**15.34 9**

**15.36 9**

**15.38 7**

**15.4 5**

**15.42 5**

**15.44 9**

**15.46 7**

**15.48 7**

**15.5 9**

**15.52 10**

**15.54 9**

**15.56 10**

**15.58 7**

**15.6 7**

**15.62 6**

**15.64 3**

**15.66 4**

**15.68 6**

**15.7 7**

**15.72 8**

**15.74 6**

**15.76 11**

**15.78 10**

**15.8 9**

**15.82 14**

**15.84 10**

**15.86 9**

**15.88 7**

**15.9 9**

**15.92 10**

**15.94 13**

**15.96 13**

**15.98 8**

**16 12**

**16.02 9**

**16.04 9**

**16.06 12**

**16.08 11**

**16.1 10**

**16.12 9**

**16.14 10**

**16.16 11**

**16.18 12**

**16.2 8**

**16.22 9**

**16.24 11**

**16.26 8**

**16.28 6**

**16.3 8**

**16.32 10**

**16.34 16**

**16.36 13**

**16.38 10**

**16.4 7**

**16.42 7**

**16.44 10**

**16.46 9**

**16.48 7**

**16.5 11**

**16.52 12**

**16.54 8**

**16.56 10**

**16.58 6**

**16.6 6**

**16.62 8**

**16.64 9**

**16.66 10**

**16.68 8**

**16.7 8**

**16.72 7**

**16.74 8**

**16.76 7**

**16.78 9**

**16.8 10**

**16.82 11**

**16.84 10**

**16.86 10**

**16.88 8**

**16.9 7**

**16.92 7**

**16.94 6**

**16.96 6**

**16.98 8**

**17 10**

**17.02 8**

**17.04 5**

**17.06 5**

**17.08 5**

**17.1 7**

**17.12 7**

**17.14 6**

**17.16 8**

**17.18 10**

**17.2 9**

**17.22 11**

**17.24 11**

**17.26 12**

**17.28 14**

**17.3 8**

**17.32 3**

**17.34 8**

**17.36 12**

**17.38 8**

**17.4 5**

**17.42 8**

**17.44 9**

**17.46 9**

**17.48 5**

**17.5 6**

**17.52 7**

**17.54 6**

**17.56 4**

**17.58 7**

**17.6 7**

**17.62 7**

**17.64 5**

**17.66 6**

**17.68 9**

**17.7 8**

**17.72 7**

**17.74 8**

**17.76 8**

**17.78 5**

**17.8 4**

**17.82 6**

**17.84 7**

**17.86 11**

**17.88 11**

**17.9 8**

**17.92 8**

**17.94 6**

**17.96 8**

**17.98 7**

**18 6**

**18.02 7**

**18.04 11**

**18.06 9**

**18.08 13**

**18.1 12**

**18.12 8**

**18.14 8**

**18.16 9**

**18.18 6**

**18.2 6**

**18.22 8**

**18.24 10**

**18.26 10**

**18.28 7**

**18.3 6**

**18.32 5**

**18.34 5**

**18.36 7**

**18.38 10**

**18.4 8**

**18.42 10**

**18.44 10**

**18.46 12**

**18.48 13**

**18.5 11**

**18.52 13**

**18.54 14**

**18.56 8**

**18.58 7**

**18.6 11**

**18.62 11**

**18.64 12**

**18.66 13**

**18.68 11**

**18.7 11**

**18.72 12**

**18.74 15**

**18.76 16**

**18.78 16**

**18.8 17**

**18.82 21**

**18.84 20**

**18.86 17**

**18.88 20**

**18.9 24**

**18.92 20**

**18.94 17**

**18.96 18**

**18.98 25**

**19 25**

**19.02 22**

**19.04 24**

**19.06 23**

**19.08 21**

**19.1 17**

**19.12 19**

**19.14 17**

**19.16 14**

**19.18 12**

**19.2 13**

**19.22 16**

**19.24 12**

**19.26 12**

**19.28 13**

**19.3 12**

**19.32 14**

**19.34 15**

**19.36 10**

**19.38 9**

**19.4 10**

**19.42 11**

**19.44 10**

**19.46 9**

**19.48 13**

**19.5 15**

**19.52 12**

**19.54 9**

**19.56 9**

**19.58 10**

**19.6 10**

**19.62 8**

**19.64 6**

**19.66 9**

**19.68 12**

**19.7 8**

**19.72 8**

**19.74 12**

**19.76 13**

**19.78 8**

**19.8 6**

**19.82 9**

**19.84 10**

**19.86 9**

**19.88 7**

**19.9 7**

**19.92 6**

**19.94 10**

**19.96 13**

**19.98 10**

**20 7**

**20.02 8**

**20.04 8**

**20.06 6**

**20.08 7**

**20.1 11**

**20.12 11**

**20.14 8**

**20.16 10**

**20.18 10**

**20.2 10**

**20.22 9**

**20.24 10**

**20.26 14**

**20.28 13**

**20.3 10**

**20.32 8**

**20.34 9**

**20.36 10**

**20.38 9**

**20.4 7**

**20.42 12**

**20.44 11**

**20.46 7**

**20.48 8**

**20.5 9**

**20.52 9**

**20.54 8**

**20.56 7**

**20.58 7**

**20.6 8**

**20.62 8**

**20.64 8**

**20.66 9**

**20.68 8**

**20.7 7**

**20.72 7**

**20.74 9**

**20.76 10**

**20.78 9**

**20.8 7**

**20.82 7**

**20.84 7**

**20.86 8**

**20.88 11**

**20.9 8**

**20.92 10**

**20.94 10**

**20.96 8**

**20.98 12**

**21 13**

**21.02 12**

**21.04 12**

**21.06 10**

**21.08 10**

**21.1 10**

**21.12 7**

**21.14 6**

**21.16 7**

**21.18 9**

**21.2 11**

**21.22 9**

**21.24 11**

**21.26 9**

**21.28 8**

**21.3 10**

**21.32 10**

**21.34 9**

**21.36 10**

**21.38 12**

**21.4 9**

**21.42 9**

**21.44 8**

**21.46 8**

**21.48 8**

**21.5 11**

**21.52 10**

**21.54 13**

**21.56 11**

**21.58 13**

**21.6 14**

**21.62 15**

**21.64 15**

**21.66 10**

**21.68 8**

**21.7 10**

**21.72 10**

**21.74 11**

**21.76 12**

**21.78 7**

**21.8 11**

**21.82 13**

**21.84 14**

**21.86 16**

**21.88 10**

**21.9 6**

**21.92 11**

**21.94 10**

**21.96 7**

**21.98 12**

**22 6**

**22.02 6**

**22.04 7**

**22.06 10**

**22.08 8**

**22.1 6**

**22.12 8**

**22.14 8**

**22.16 9**

**22.18 8**

**22.2 10**

**22.22 10**

**22.24 7**

**22.26 10**

**22.28 14**

**22.3 15**

**22.32 11**

**22.34 8**

**22.36 9**

**22.38 7**

**22.4 7**

**22.42 9**

**22.44 7**

**22.46 6**

**22.48 11**

**22.5 18**

**22.52 10**

**22.54 11**

**22.56 7**

**22.58 8**

**22.6 10**

**22.62 14**

**22.64 16**

**22.66 12**

**22.68 13**

**22.7 13**

**22.72 10**

**22.74 10**

**22.76 11**

**22.78 13**

**22.8 15**

**22.82 14**

**22.84 15**

**22.86 14**

**22.88 21**

**22.9 21**

**22.92 17**

**22.94 17**

**22.96 20**

**22.98 23**

**23 26**

**23.02 29**

**23.04 29**

**23.06 31**

**23.08 38**

**23.1 48**

**23.12 36**

**23.14 47**

**23.16 54**

**23.18 58**

**23.2 53**

**23.22 52**

**23.24 49**

**23.26 60**

**23.28 56**

**23.3 52**

**23.32 57**

**23.34 58**

**23.36 56**

**23.38 52**

**23.4 49**

**23.42 40**

**23.44 45**

**23.46 54**

**23.48 36**

**23.5 35**

**23.52 34**

**23.54 27**

**23.56 24**

**23.58 27**

**23.6 22**

**23.62 27**

**23.64 24**

**23.66 26**

**23.68 26**

**23.7 25**

**23.72 24**

**23.74 23**

**23.76 27**

**23.78 27**

**23.8 20**

**23.82 21**

**23.84 25**

**23.86 16**

**23.88 15**

**23.9 16**

**23.92 18**

**23.94 20**

**23.96 18**

**23.98 15**

**24 21**

**24.02 21**

**24.04 18**

**24.06 20**

**24.08 19**

**24.1 20**

**24.12 21**

**24.14 11**

**24.16 16**

**24.18 14**

**24.2 17**

**24.22 17**

**24.24 11**

**24.26 16**

**24.28 16**

**24.3 15**

**24.32 14**

**24.34 9**

**24.36 15**

**24.38 14**

**24.4 11**

**24.42 11**

**24.44 13**

**24.46 16**

**24.48 15**

**24.5 23**

**24.52 10**

**24.54 15**

**24.56 12**

**24.58 14**

**24.6 11**

**24.62 9**

**24.64 14**

**24.66 11**

**24.68 15**

**24.7 16**

**24.72 17**

**24.74 10**

**24.76 16**

**24.78 13**

**24.8 9**

**24.82 16**

**24.84 8**

**24.86 13**

**24.88 17**

**24.9 14**

**24.92 27**

**24.94 23**

**24.96 13**

**24.98 22**

**25 26**

**25.02 21**

**25.04 27**

**25.06 33**

**25.08 23**

**25.1 39**

**25.12 38**

**25.14 37**

**25.16 46**

**25.18 49**

**25.2 51**

**25.22 48**

**25.24 57**

**25.26 67**

**25.28 76**

**25.3 58**

**25.32 70**

**25.34 89**

**25.36 85**

**25.38 75**

**25.4 77**

**25.42 66**

**25.44 56**

**25.46 62**

**25.48 77**

**25.5 71**

**25.52 50**

**25.54 44**

**25.56 55**

**25.58 67**

**25.6 43**

**25.62 56**

**25.64 58**

**25.66 42**

**25.68 36**

**25.7 37**

**25.72 40**

**25.74 48**

**25.76 35**

**25.78 28**

**25.8 30**

**25.82 30**

**25.84 40**

**25.86 38**

**25.88 38**

**25.9 32**

**25.92 36**

**25.94 43**

**25.96 33**

**25.98 32**

**26 49**

**26.02 47**

**26.04 50**

**26.06 55**

**26.08 76**

**26.1 67**

**26.12 79**

**26.14 80**

**26.16 98**

**26.18 113**

**26.2 126**

**26.22 181**

**26.24 180**

**26.26 240**

**26.28 210**

**26.3 273**

**26.32 318**

**26.34 366**

**26.36 379**

**26.38 418**

**26.4 441**

**26.42 423**

**26.44 442**

**26.46 423**

**26.48 413**

**26.5 428**

**26.52 404**

**26.54 422**

**26.56 389**

**26.58 368**

**26.6 349**

**26.62 365**

**26.64 298**

**26.66 289**

**26.68 256**

**26.7 218**

**26.72 216**

**26.74 171**

**26.76 170**

**26.78 127**

**26.8 125**

**26.82 121**

**26.84 100**

**26.86 90**

**26.88 94**

**26.9 72**

**26.92 84**

**26.94 79**

**26.96 92**

**26.98 75**

**27 80**

**27.02 83**

**27.04 107**

**27.06 103**

**27.08 115**

**27.1 97**

**27.12 104**

**27.14 106**

**27.16 108**

**27.18 92**

**27.2 112**

**27.22 104**

**27.24 94**

**27.26 108**

**27.28 91**

**27.3 108**

**27.32 95**

**27.34 88**

**27.36 83**

**27.38 80**

**27.4 76**

**27.42 64**

**27.44 77**

**27.46 60**

**27.48 47**

**27.5 40**

**27.52 50**

**27.54 58**

**27.56 51**

**27.58 45**

**27.6 46**

**27.62 45**

**27.64 48**

**27.66 46**

**27.68 40**

**27.7 45**

**27.72 37**

**27.74 28**

**27.76 35**

**27.78 32**

**27.8 29**

**27.82 36**

**27.84 27**

**27.86 22**

**27.88 23**

**27.9 25**

**27.92 24**

**27.94 30**

**27.96 33**

**27.98 25**

**28 29**

**28.02 42**

**28.04 39**

**28.06 31**

**28.08 29**

**28.1 36**

**28.12 48**

**28.14 48**

**28.16 43**

**28.18 52**

**28.2 46**

**28.22 57**

**28.24 60**

**28.26 73**

**28.28 74**

**28.3 85**

**28.32 101**

**28.34 105**

**28.36 106**

**28.38 125**

**28.4 124**

**28.42 122**

**28.44 142**

**28.46 125**

**28.48 122**

**28.5 116**

**28.52 118**

**28.54 101**

**28.56 103**

**28.58 111**

**28.6 92**

**28.62 73**

**28.64 81**

**28.66 73**

**28.68 65**

**28.7 69**

**28.72 54**

**28.74 51**

**28.76 41**

**28.78 43**

**28.8 43**

**28.82 35**

**28.84 30**

**28.86 17**

**28.88 17**

**28.9 20**

**28.92 18**

**28.94 28**

**28.96 23**

**28.98 22**

**29 17**

**29.02 17**

**29.04 18**

**29.06 15**

**29.08 20**

**29.1 21**

**29.12 20**

**29.14 16**

**29.16 16**

**29.18 16**

**29.2 15**

**29.22 19**

**29.24 16**

**29.26 14**

**29.28 14**

**29.3 18**

**29.32 14**

**29.34 12**

**29.36 13**

**29.38 17**

**29.4 14**

**29.42 11**

**29.44 9**

**29.46 13**

**29.48 13**

**29.5 13**

**29.52 9**

**29.54 14**

**29.56 13**

**29.58 17**

**29.6 15**

**29.62 21**

**29.64 17**

**29.66 19**

**29.68 22**

**29.7 17**

**29.72 16**

**29.74 15**

**29.76 16**

**29.78 13**

**29.8 14**

**29.82 15**

**29.84 8**

**29.86 14**

**29.88 17**

**29.9 14**

**29.92 10**

**29.94 10**

**29.96 12**

**29.98 16**

**30 13**

**30.02 8**

**30.04 10**

**30.06 11**

**30.08 14**

**30.1 9**

**30.12 12**

**30.14 13**

**30.16 9**

**30.18 10**

**30.2 10**

**30.22 13**

**30.24 10**

**30.26 13**

**30.28 10**

**30.3 11**

**30.32 16**

**30.34 15**

**30.36 12**

**30.38 15**

**30.4 12**

**30.42 7**

**30.44 7**

**30.46 9**

**30.48 11**

**30.5 12**

**30.52 10**

**30.54 10**

**30.56 11**

**30.58 10**

**30.6 7**

**30.62 8**

**30.64 11**

**30.66 10**

**30.68 11**

**30.7 10**

**30.72 11**

**30.74 10**

**30.76 9**

**30.78 11**

**30.8 7**

**30.82 8**

**30.84 14**

**30.86 16**

**30.88 14**

**30.9 13**

**30.92 12**

**30.94 11**

**30.96 11**

**30.98 9**

**31 13**

**31.02 13**

**31.04 8**

**31.06 8**

**31.08 13**

**31.1 10**

**31.12 11**

**31.14 12**

**31.16 9**

**31.18 12**

**31.2 14**

**31.22 11**

**31.24 8**

**31.26 7**

**31.28 10**

**31.3 11**

**31.32 12**

**31.34 10**

**31.36 8**

**31.38 10**

**31.4 13**

**31.42 18**

**31.44 16**

**31.46 10**

**31.48 12**

**31.5 15**

**31.52 15**

**31.54 16**

**31.56 13**

**31.58 15**

**31.6 22**

**31.62 19**

**31.64 15**

**31.66 18**

**31.68 23**

**31.7 21**

**31.72 21**

**31.74 27**

**31.76 34**

**31.78 32**

**31.8 30**

**31.82 31**

**31.84 45**

**31.86 54**

**31.88 46**

**31.9 59**

**31.92 68**

**31.94 62**

**31.96 61**

**31.98 59**

**32 66**

**32.02 77**

**32.04 80**

**32.06 69**

**32.08 59**

**32.1 61**

**32.12 76**

**32.14 84**

**32.16 77**

**32.18 69**

**32.2 63**

**32.22 61**

**32.24 55**

**32.26 65**

**32.28 64**

**32.3 52**

**32.32 59**

**32.34 62**

**32.36 53**

**32.38 53**

**32.4 53**

**32.42 52**

**32.44 54**

**32.46 58**

**32.48 60**

**32.5 55**

**32.52 56**

**32.54 51**

**32.56 54**

**32.58 62**

**32.6 52**

**32.62 48**

**32.64 53**

**32.66 58**

**32.68 51**

**32.7 53**

**32.72 57**

**32.74 58**

**32.76 58**

**32.78 57**

**32.8 56**

**32.82 59**

**32.84 58**

**32.86 48**

**32.88 40**

**32.9 42**

**32.92 45**

**32.94 42**

**32.96 31**

**32.98 30**

**33 32**

**33.02 33**

**33.04 39**

**33.06 34**

**33.08 28**

**33.1 33**

**33.12 31**

**33.14 30**

**33.16 27**

**33.18 25**

**33.2 22**

**33.22 23**

**33.24 30**

**33.26 25**

**33.28 27**

**33.3 26**

**33.32 21**

**33.34 29**

**33.36 31**

**33.38 30**

**33.4 31**

**33.42 32**

**33.44 30**

**33.46 32**

**33.48 34**

**33.5 45**

**33.52 51**

**33.54 51**

**33.56 59**

**33.58 61**

**33.6 63**

**33.62 67**

**33.64 76**

**33.66 75**

**33.68 74**

**33.7 81**

**33.72 66**

**33.74 59**

**33.76 60**

**33.78 54**

**33.8 60**

**33.82 44**

**33.84 37**

**33.86 36**

**33.88 35**

**33.9 32**

**33.92 34**

**33.94 35**

**33.96 35**

**33.98 36**

**34 19**

**34.02 18**

**34.04 19**

**34.06 12**

**34.08 13**

**34.1 20**

**34.12 25**

**34.14 16**

**34.16 15**

**34.18 15**

**34.2 14**

**34.22 14**

**34.24 13**

**34.26 14**

**34.28 14**

**34.3 12**

**34.32 10**

**34.34 13**

**34.36 14**

**34.38 10**

**34.4 10**

**34.42 8**

**34.44 7**

**34.46 8**

**34.48 11**

**34.5 11**

**34.52 10**

**34.54 10**

**34.56 10**

**34.58 10**

**34.6 10**

**34.62 10**

**34.64 13**

**34.66 10**

**34.68 11**

**34.7 6**

**34.72 6**

**34.74 7**

**34.76 11**

**34.78 8**

**34.8 9**

**34.82 8**

**34.84 10**

**34.86 13**

**34.88 9**

**34.9 11**

**34.92 9**

**34.94 16**

**34.96 10**

**34.98 14**

**35 14**

**35.02 13**

**35.04 16**

**35.06 11**

**35.08 11**

**35.1 9**

**35.12 8**

**35.14 8**

**35.16 9**

**35.18 19**

**35.2 10**

**35.22 13**

**35.24 11**

**35.26 13**

**35.28 15**

**35.3 13**

**35.32 14**

**35.34 20**

**35.36 19**

**35.38 26**

**35.4 31**

**35.42 41**

**35.44 41**

**35.46 49**

**35.48 46**

**35.5 43**

**35.52 50**

**35.54 42**

**35.56 31**

**35.58 32**

**35.6 23**

**35.62 19**

**35.64 28**

**35.66 24**

**35.68 25**

**35.7 26**

**35.72 25**

**35.74 23**

**35.76 13**

**35.78 12**

**35.8 17**

**35.82 17**

**35.84 17**

**35.86 9**

**35.88 15**

**35.9 14**

**35.92 15**

**35.94 11**

**35.96 11**

**35.98 11**

**36 4**

**36.02 8**

**36.04 11**

**36.06 12**

**36.08 11**

**36.1 10**

**36.12 14**

**36.14 14**

**36.16 8**

**36.18 12**

**36.2 8**

**36.22 13**

**36.24 14**

**36.26 9**

**36.28 10**

**36.3 15**

**36.32 9**

**36.34 12**

**36.36 17**

**36.38 16**

**36.4 24**

**36.42 16**

**36.44 16**

**36.46 25**

**36.48 27**

**36.5 22**

**36.52 28**

**36.54 28**

**36.56 25**

**36.58 25**

**36.6 30**

**36.62 49**

**36.64 36**

**36.66 36**

**36.68 39**

**36.7 41**

**36.72 34**

**36.74 35**

**36.76 38**

**36.78 34**

**36.8 29**

**36.82 38**

**36.84 38**

**36.86 33**

**36.88 35**

**36.9 33**

**36.92 35**

**36.94 24**

**36.96 30**

**36.98 29**

**37 24**

**37.02 17**

**37.04 19**

**37.06 14**

**37.08 19**

**37.1 11**

**37.12 12**

**37.14 16**

**37.16 16**

**37.18 11**

**37.2 13**

**37.22 13**

**37.24 17**

**37.26 12**

**37.28 14**

**37.3 13**

**37.32 12**

**37.34 13**

**37.36 16**

**37.38 14**

**37.4 8**

**37.42 13**

**37.44 10**

**37.46 8**

**37.48 14**

**37.5 14**

**37.52 9**

**37.54 8**

**37.56 10**

**37.58 11**

**37.6 8**

**37.62 8**

**37.64 5**

**37.66 11**

**37.68 10**

**37.7 9**

**37.72 6**

**37.74 9**

**37.76 11**

**37.78 11**

**37.8 7**

**37.82 9**

**37.84 12**

**37.86 14**

**37.88 11**

**37.9 11**

**37.92 11**

**37.94 11**

**37.96 19**

**37.98 10**

**38 9**

**38.02 17**

**38.04 12**

**38.06 13**

**38.08 12**

**38.1 7**

**38.12 13**

**38.14 14**

**38.16 12**

**38.18 11**

**38.2 15**

**38.22 13**

**38.24 13**

**38.26 16**

**38.28 12**

**38.3 19**

**38.32 15**

**38.34 29**

**38.36 16**

**38.38 15**

**38.4 20**

**38.42 20**

**38.44 20**

**38.46 19**

**38.48 27**

**38.5 18**

**38.52 33**

**38.54 26**

**38.56 27**

**38.58 25**

**38.6 32**

**38.62 31**

**38.64 32**

**38.66 41**

**38.68 42**

**38.7 48**

**38.72 34**

**38.74 41**

**38.76 50**

**38.78 42**

**38.8 44**

**38.82 49**

**38.84 41**

**38.86 61**

**38.88 43**

**38.9 40**

**38.92 52**

**38.94 34**

**38.96 39**

**38.98 33**

**39 43**

**39.02 30**

**39.04 28**

**39.06 36**

**39.08 35**

**39.1 19**

**39.12 24**

**39.14 22**

**39.16 30**

**39.18 20**

**39.2 23**

**39.22 18**

**39.24 12**

**39.26 14**

**39.28 18**

**39.3 5**

**39.32 11**

**39.34 5**

**39.36 6**

**39.38 9**

**39.4 15**

**39.42 8**

**39.44 16**

**39.46 8**

**39.48 11**

**39.5 9**

**39.52 20**

**39.54 16**

**39.56 12**

**39.58 12**

**39.6 13**

**39.62 14**

**39.64 14**

**39.66 11**

**39.68 11**

**39.7 4**

**39.72 10**

**39.74 10**

**39.76 20**

**39.78 21**

**39.8 11**

**39.82 13**

**39.84 28**

**39.86 15**

**39.88 19**

**39.9 16**

**39.92 17**

**39.94 15**

**39.96 27**

**39.98 20**

**40 29**

**40.02 29**

**40.04 26**

**40.06 32**

**40.08 32**

**40.1 28**

**40.12 38**

**40.14 35**

**40.16 57**

**40.18 46**

**40.2 52**

**40.22 51**

**40.24 42**

**40.26 44**

**40.28 38**

**40.3 47**

**40.32 35**

**40.34 37**

**40.36 48**

**40.38 36**

**40.4 29**

**40.42 40**

**40.44 39**

**40.46 37**

**40.48 28**

**40.5 32**

**40.52 23**

**40.54 17**

**40.56 30**

**40.58 15**

**40.6 18**

**40.62 25**

**40.64 11**

**40.66 15**

**40.68 18**

**40.7 22**

**40.72 15**

**40.74 21**

**40.76 21**

**40.78 24**

**40.8 27**

**40.82 30**

**40.84 19**

**40.86 24**

**40.88 29**

**40.9 18**

**40.92 9**

**40.94 15**

**40.96 20**

**40.98 18**

**41 14**

**41.02 15**

**41.04 10**

**41.06 18**

**41.08 16**

**41.1 16**

**41.12 18**

**41.14 13**

**41.16 19**

**41.18 13**

**41.2 16**

**41.22 14**

**41.24 17**

**41.26 20**

**41.28 17**

**41.3 15**

**41.32 13**

**41.34 16**

**41.36 23**

**41.38 24**

**41.4 23**

**41.42 19**

**41.44 27**

**41.46 21**

**41.48 21**

**41.5 17**

**41.52 30**

**41.54 22**

**41.56 25**

**41.58 23**

**41.6 26**

**41.62 23**

**41.64 26**

**41.66 33**

**41.68 21**

**41.7 24**

**41.72 29**

**41.74 29**

**41.76 18**

**41.78 28**

**41.8 17**

**41.82 18**

**41.84 27**

**41.86 19**

**41.88 20**

**41.9 18**

**41.92 21**

**41.94 14**

**41.96 14**

**41.98 22**

**42 19**

**42.02 12**

**42.04 12**

**42.06 9**

**42.08 6**

**42.1 6**

**42.12 9**

**42.14 12**

**42.16 13**

**42.18 10**

**42.2 17**

**42.22 13**

**42.24 6**

**42.26 10**

**42.28 16**

**42.3 12**

**42.32 14**

**42.34 15**

**42.36 9**

**42.38 20**

**42.4 12**

**42.42 17**

**42.44 13**

**42.46 18**

**42.48 15**

**42.5 17**

**42.52 16**

**42.54 18**

**42.56 17**

**42.58 23**

**42.6 18**

**42.62 20**

**42.64 21**

**42.66 20**

**42.68 18**

**42.7 21**

**42.72 14**

**42.74 13**

**42.76 25**

**42.78 23**

**42.8 20**

**42.82 15**

**42.84 23**

**42.86 18**

**42.88 20**

**42.9 21**

**42.92 19**

**42.94 25**

**42.96 23**

**42.98 26**

**43 31**

**43.02 22**

**43.04 18**

**43.06 33**

**43.08 38**

**43.1 32**

**43.12 29**

**43.14 28**

**43.16 27**

**43.18 29**

**43.2 35**

**43.22 50**

**43.24 47**

**43.26 40**

**43.28 40**

**43.3 44**

**43.32 45**

**43.34 55**

**43.36 48**

**43.38 55**

**43.4 51**

**43.42 45**

**43.44 58**

**43.46 55**

**43.48 53**

**43.5 51**

**43.52 54**

**43.54 61**

**43.56 57**

**43.58 49**

**43.6 46**

**43.62 45**

**43.64 43**

**43.66 44**

**43.68 43**

**43.7 38**

**43.72 45**

**43.74 40**

**43.76 40**

**43.78 35**

**43.8 44**

**43.82 39**

**43.84 38**

**43.86 34**

**43.88 36**

**43.9 31**

**43.92 23**

**43.94 21**

**43.96 23**

**43.98 24**

**44 18**

**44.02 22**

**44.04 15**

**44.06 14**

**44.08 12**

**44.1 16**

**44.12 12**

**44.14 16**

**44.16 17**

**44.18 18**

**44.2 15**

**44.22 14**

**44.24 15**

**44.26 10**

**44.28 8**

**44.3 9**

**44.32 13**

**44.34 12**

**44.36 7**

**44.38 10**

**44.4 7**

**44.42 9**

**44.44 10**

**44.46 12**

**44.48 8**

**44.5 9**

**44.52 6**

**44.54 12**

**44.56 11**

**44.58 15**

**44.6 8**

**44.62 15**

**44.64 20**

**44.66 12**

**44.68 12**

**44.7 13**

**44.72 15**

**44.74 12**

**44.76 12**

**44.78 20**

**44.8 15**

**44.82 13**

**44.84 17**

**44.86 16**

**44.88 14**

**44.9 13**

**44.92 10**

**44.94 15**

**44.96 22**

**44.98 21**

**45 21**

**45.02 19**

**45.04 21**

**45.06 18**

**45.08 25**

**45.1 20**

**45.12 23**

**45.14 24**

**45.16 22**

**45.18 26**

**45.2 26**

**45.22 27**

**45.24 29**

**45.26 24**

**45.28 29**

**45.3 21**

**45.32 21**

**45.34 27**

**45.36 23**

**45.38 19**

**45.4 20**

**45.42 20**

**45.44 18**

**45.46 26**

**45.48 23**

**45.5 23**

**45.52 23**

**45.54 25**

**45.56 19**

**45.58 24**

**45.6 20**

**45.62 19**

**45.64 17**

**45.66 16**

**45.68 14**

**45.7 13**

**45.72 18**

**45.74 14**

**45.76 16**

**45.78 11**

**45.8 8**

**45.82 15**

**45.84 14**

**45.86 15**

**45.88 16**

**45.9 14**

**45.92 13**

**45.94 14**

**45.96 14**

**45.98 13**

**46 16**

**46.02 12**

**46.04 18**

**46.06 12**

**46.08 11**

**46.1 11**

**46.12 16**

**46.14 16**

**46.16 14**

**46.18 10**

**46.2 15**

**46.22 10**

**46.24 16**

**46.26 19**

**46.28 21**

**46.3 18**

**46.32 21**

**46.34 12**

**46.36 25**

**46.38 27**

**46.4 20**

**46.42 17**

**46.44 15**

**46.46 12**

**46.48 15**

**46.5 14**

**46.52 15**

**46.54 16**

**46.56 16**

**46.58 15**

**46.6 20**

**46.62 17**

**46.64 12**

**46.66 18**

**46.68 18**

**46.7 22**

**46.72 20**

**46.74 19**

**46.76 12**

**46.78 20**

**46.8 20**

**46.82 24**

**46.84 27**

**46.86 25**

**46.88 34**

**46.9 30**

**46.92 24**

**46.94 31**

**46.96 29**

**46.98 32**

**47 29**

**47.02 33**

**47.04 33**

**47.06 44**

**47.08 39**

**47.1 31**

**47.12 35**

**47.14 42**

**47.16 52**

**47.18 43**

**47.2 36**

**47.22 40**

**47.24 37**

**47.26 52**

**47.28 45**

**47.3 44**

**47.32 37**

**47.34 41**

**47.36 43**

**47.38 39**

**47.4 40**

**47.42 42**

**47.44 38**

**47.46 50**

**47.48 49**

**47.5 36**

**47.52 35**

**47.54 30**

**47.56 36**

**47.58 46**

**47.6 44**

**47.62 37**

**47.64 45**

**47.66 53**

**47.68 40**

**47.7 28**

**47.72 34**

**47.74 34**

**47.76 26**

**47.78 29**

**47.8 28**

**47.82 24**

**47.84 25**

**47.86 20**

**47.88 24**

**47.9 24**

**47.92 26**

**47.94 17**

**47.96 14**

**47.98 18**

**48 19**

**48.02 21**

**48.04 17**

**48.06 18**

**48.08 25**

**48.1 18**

**48.12 16**

**48.14 18**

**48.16 16**

**48.18 14**

**48.2 17**

**48.22 14**

**48.24 11**

**48.26 10**

**48.28 15**

**48.3 14**

**48.32 11**

**48.34 16**

**48.36 13**

**48.38 12**

**48.4 11**

**48.42 10**

**48.44 8**

**48.46 9**

**48.48 8**

**48.5 12**

**48.52 15**

**48.54 13**

**48.56 17**

**48.58 9**

**48.6 14**

**48.62 14**

**48.64 15**

**48.66 10**

**48.68 10**

**48.7 15**

**48.72 13**

**48.74 16**

**48.76 19**

**48.78 16**

**48.8 15**

**48.82 13**

**48.84 14**

**48.86 12**

**48.88 12**

**48.9 14**

**48.92 16**

**48.94 13**

**48.96 10**

**48.98 15**

**49 14**

**49.02 14**

**49.04 10**

**49.06 15**

**49.08 11**

**49.1 18**

**49.12 13**

**49.14 9**

**49.16 15**

**49.18 16**

**49.2 12**

**49.22 13**

**49.24 16**

**49.26 10**

**49.28 10**

**49.3 11**

**49.32 13**

**49.34 8**

**49.36 10**

**49.38 13**

**49.4 12**

**49.42 11**

**49.44 11**

**49.46 13**

**49.48 14**

**49.5 12**

**49.52 18**

**49.54 12**

**49.56 11**

**49.58 13**

**49.6 11**

**49.62 10**

**49.64 12**

**49.66 9**

**49.68 10**

**49.7 12**

**49.72 11**

**49.74 8**

**49.76 8**

**49.78 14**

**49.8 12**

**49.82 12**

**49.84 11**

**49.86 10**

**49.88 16**

**49.9 11**

**49.92 13**

**49.94 12**

**49.96 13**

**49.98 9**

**50 12**

**50.02 7**

**50.04 11**

**50.06 15**

**50.08 16**

**50.1 10**

**50.12 14**

**50.14 9**

**50.16 10**

**50.18 13**

**50.2 12**

**50.22 11**

**50.24 9**

**50.26 9**

**50.28 11**

**50.3 12**

**50.32 10**

**50.34 11**

**50.36 12**

**50.38 10**

**50.4 11**

**50.42 5**

**50.44 6**

**50.46 4**

**50.48 6**

**50.5 7**

**50.52 3**

**50.54 12**

**50.56 6**

**50.58 4**

**50.6 6**

**50.62 4**

**50.64 10**

**50.66 4**

**50.68 6**

**50.7 7**

**50.72 5**

**50.74 9**

**50.76 8**

**50.78 6**

**50.8 11**

**50.82 10**

**50.84 11**

**50.86 15**

**50.88 9**

**50.9 9**

**50.92 6**

**50.94 8**

**50.96 9**

**50.98 12**

**51 11**

**51.02 5**

**51.04 7**

**51.06 10**

**51.08 14**

**51.1 15**

**51.12 18**

**51.14 14**

**51.16 6**

**51.18 9**

**51.2 8**

**51.22 15**

**51.24 8**

**51.26 12**

**51.28 12**

**51.3 16**

**51.32 14**

**51.34 10**

**51.36 10**

**51.38 13**

**51.4 10**

**51.42 12**

**51.44 9**

**51.46 11**

**51.48 14**

**51.5 5**

**51.52 14**

**51.54 12**

**51.56 11**

**51.58 14**

**51.6 10**

**51.62 12**

**51.64 13**

**51.66 15**

**51.68 14**

**51.7 11**

**51.72 13**

**51.74 11**

**51.76 18**

**51.78 16**

**51.8 12**

**51.82 12**

**51.84 24**

**51.86 19**

**51.88 23**

**51.9 14**

**51.92 17**

**51.94 20**

**51.96 18**

**51.98 23**

**52 31**

**52.02 28**

**52.04 29**

**52.06 40**

**52.08 52**

**52.1 35**

**52.12 27**

**52.14 27**

**52.16 35**

**52.18 40**

**52.2 38**

**52.22 37**

**52.24 33**

**52.26 34**

**52.28 34**

**52.3 37**

**52.32 33**

**52.34 22**

**52.36 25**

**52.38 26**

**52.4 30**

**52.42 36**

**52.44 27**

**52.46 18**

**52.48 15**

**52.5 14**

**52.52 28**

**52.54 15**

**52.56 14**

**52.58 22**

**52.6 19**

**52.62 20**

**52.64 25**

**52.66 18**

**52.68 21**

**52.7 18**

**52.72 23**

**52.74 34**

**52.76 27**

**52.78 26**

**52.8 27**

**52.82 27**

**52.84 21**

**52.86 20**

**52.88 22**

**52.9 25**

**52.92 15**

**52.94 20**

**52.96 22**

**52.98 16**

**53 14**

**53.02 16**

**53.04 10**

**53.06 19**

**53.08 18**

**53.1 17**

**53.12 23**

**53.14 22**

**53.16 20**

**53.18 19**

**53.2 18**

**53.22 24**

**53.24 28**

**53.26 26**

**53.28 20**

**53.3 28**

**53.32 28**

**53.34 31**

**53.36 27**

**53.38 26**

**53.4 42**

**53.42 38**

**53.44 32**

**53.46 32**

**53.48 31**

**53.5 37**

**53.52 34**

**53.54 39**

**53.56 34**

**53.58 35**

**53.6 40**

**53.62 41**

**53.64 40**

**53.66 40**

**53.68 32**

**53.7 36**

**53.72 38**

**53.74 34**

**53.76 39**

**53.78 42**

**53.8 26**

**53.82 47**

**53.84 33**

**53.86 20**

**53.88 33**

**53.9 31**

**53.92 29**

**53.94 24**

**53.96 25**

**53.98 16**

**54 23**

**54.02 29**

**54.04 24**

**54.06 14**

**54.08 22**

**54.1 19**

**54.12 18**

**54.14 24**

**54.16 15**

**54.18 16**

**54.2 14**

**54.22 21**

**54.24 12**

**54.26 28**

**54.28 17**

**54.3 22**

**54.32 26**

**54.34 25**

**54.36 31**

**54.38 30**

**54.4 23**

**54.42 29**

**54.44 30**

**54.46 25**

**54.48 15**

**54.5 39**

**54.52 31**

**54.54 36**

**54.56 26**

**54.58 35**

**54.6 29**

**54.62 24**

**54.64 40**

**54.66 27**

**54.68 28**

**54.7 28**

**54.72 35**

**54.74 18**

**54.76 32**

**54.78 20**

**54.8 25**

**54.82 14**

**54.84 22**

**54.86 19**

**54.88 16**

**54.9 8**

**54.92 18**

**54.94 15**

**54.96 25**

**54.98 21**

**55 14**

**55.02 17**

**55.04 12**

**55.06 15**

**55.08 12**

**55.1 15**

**55.12 11**

**55.14 15**

**55.16 14**

**55.18 17**

**55.2 17**

**55.22 21**

**55.24 12**

**55.26 22**

**55.28 11**

**55.3 16**

**55.32 17**

**55.34 15**

**55.36 15**

**55.38 24**

**55.4 16**

**55.42 23**

**55.44 19**

**55.46 14**

**55.48 20**

**55.5 26**

**55.52 34**

**55.54 23**

**55.56 35**

**55.58 27**

**55.6 27**

**55.62 30**

**55.64 28**

**55.66 26**

**55.68 29**

**55.7 20**

**55.72 28**

**55.74 26**

**55.76 35**

**55.78 31**

**55.8 31**

**55.82 25**

**55.84 25**

**55.86 28**

**55.88 24**

**55.9 24**

**55.92 19**

**55.94 24**

**55.96 27**

**55.98 33**

**56 28**

**56.02 34**

**56.04 39**

**56.06 34**

**56.08 23**

**56.1 25**

**56.12 31**

**56.14 39**

**56.16 28**

**56.18 28**

**56.2 34**

**56.22 38**

**56.24 31**

**56.26 22**

**56.28 28**

**56.3 21**

**56.32 32**

**56.34 34**

**56.36 17**

**56.38 18**

**56.4 35**

**56.42 22**

**56.44 25**

**56.46 28**

**56.48 20**

**56.5 21**

**56.52 22**

**56.54 21**

**56.56 28**

**56.58 24**

**56.6 23**

**56.62 31**

**56.64 34**

**56.66 29**

**56.68 23**

**56.7 18**

**56.72 21**

**56.74 19**

**56.76 33**

**56.78 26**

**56.8 21**

**56.82 19**

**56.84 18**

**56.86 22**

**56.88 31**

**56.9 29**

**56.92 34**

**56.94 38**

**56.96 30**

**56.98 26**

**57 17**

**57.02 24**

**57.04 23**

**57.06 24**

**57.08 26**

**57.1 23**

**57.12 26**

**57.14 25**

**57.16 26**

**57.18 31**

**57.2 30**

**57.22 22**

**57.24 27**

**57.26 24**

**57.28 20**

**57.3 16**

**57.32 26**

**57.34 33**

**57.36 27**

**57.38 19**

**57.4 17**

**57.42 24**

**57.44 22**

**57.46 18**

**57.48 18**

**57.5 27**

**57.52 15**

**57.54 17**

**57.56 24**

**57.58 21**

**57.6 20**

**57.62 18**

**57.64 18**

**57.66 19**

**57.68 22**

**57.7 15**

**57.72 19**

**57.74 20**

**57.76 16**

**57.78 19**

**57.8 14**

**57.82 14**

**57.84 16**

**57.86 15**

**57.88 8**

**57.9 11**

**57.92 17**

**57.94 15**

**57.96 15**

**57.98 20**

**58 14**

**58.02 16**

**58.04 15**

**58.06 16**

**58.08 18**

**58.1 12**

**58.12 13**

**58.14 19**

**58.16 25**

**58.18 25**

**58.2 13**

**58.22 18**

**58.24 21**

**58.26 24**

**58.28 24**

**58.3 23**

**58.32 31**

**58.34 19**

**58.36 23**

**58.38 27**

**58.4 19**

**58.42 25**

**58.44 28**

**58.46 22**

**58.48 34**

**58.5 30**

**58.52 36**

**58.54 28**

**58.56 28**

**58.58 16**

**58.6 25**

**58.62 30**

**58.64 30**

**58.66 23**

**58.68 28**

**58.7 24**

**58.72 26**

**58.74 25**

**58.76 21**

**58.78 20**

**58.8 27**

**58.82 24**

**58.84 18**

**58.86 17**

**58.88 27**

**58.9 23**

**58.92 24**

**58.94 21**

**58.96 20**

**58.98 27**

**59 23**

**59.02 17**

**59.04 17**

**59.06 16**

**59.08 16**

**59.1 15**

**59.12 17**

**59.14 21**

**59.16 18**

**59.18 22**

**59.2 20**

**59.22 16**

**59.24 15**

**59.26 14**

**59.28 14**

**59.3 14**

**59.32 13**

**59.34 15**

**59.36 17**

**59.38 14**

**59.4 17**

**59.42 17**

**59.44 10**

**59.46 19**

**59.48 21**

**59.5 17**

**59.52 13**

**59.54 24**

**59.56 24**

**59.58 12**

**59.6 16**

**59.62 15**

**59.64 12**

**59.66 17**

**59.68 19**

**59.7 24**

**59.72 20**

**59.74 17**

**59.76 24**

**59.78 20**

**59.8 20**

**59.82 29**

**59.84 27**

**59.86 16**

**59.88 21**

**59.9 25**

**59.92 22**

**59.94 26**

**59.96 26**

**59.98 35**

**60 37**

**60.02 26**

**60.04 45**

**60.06 35**

**60.08 34**

**60.1 20**

**60.12 23**

**60.14 34**

**60.16 34**

**60.18 32**

**60.2 31**

**60.22 29**

**60.24 33**

**60.26 36**

**60.28 29**

**60.3 32**

**60.32 31**

**60.34 32**

**60.36 42**

**60.38 31**

**60.4 26**

**60.42 27**

**60.44 23**

**60.46 30**

**60.48 26**

**60.5 19**

**60.52 33**

**60.54 30**

**60.56 28**

**60.58 24**

**60.6 28**

**60.62 33**

**60.64 20**

**60.66 29**

**60.68 19**

**60.7 19**

**60.72 22**

**60.74 20**

**60.76 25**

**60.78 23**

**60.8 22**

**60.82 25**

**60.84 27**

**60.86 28**

**60.88 24**

**60.9 26**

**60.92 20**

**60.94 25**

**60.96 29**

**60.98 21**

**61 26**

**61.02 23**

**61.04 24**

**61.06 26**

**61.08 31**

**61.1 27**

**61.12 22**

**61.14 25**

**61.16 23**

**61.18 29**

**61.2 20**

**61.22 24**

**61.24 27**

**61.26 30**

**61.28 29**

**61.3 26**

**61.32 26**

**61.34 21**

**61.36 31**

**61.38 28**

**61.4 31**

**61.42 37**

**61.44 34**

**61.46 29**

**61.48 32**

**61.5 25**

**61.52 34**

**61.54 31**

**61.56 27**

**61.58 34**

**61.6 25**

**61.62 26**

**61.64 28**

**61.66 35**

**61.68 32**

**61.7 30**

**61.72 30**

**61.74 27**

**61.76 26**

**61.78 31**

**61.8 34**

**61.82 25**

**61.84 28**

**61.86 27**

**61.88 15**

**61.9 20**

**61.92 21**

**61.94 23**

**61.96 16**

**61.98 27**

**62 24**

**62.02 26**

**62.04 18**

**62.06 21**

**62.08 25**

**62.1 22**

**62.12 24**

**62.14 21**

**62.16 19**

**62.18 17**

**62.2 21**

**62.22 23**

**62.24 20**

**62.26 23**

**62.28 21**

**62.3 19**

**62.32 15**

**62.34 21**

**62.36 23**

**62.38 23**

**62.4 16**

**62.42 20**

**62.44 15**

**62.46 12**

**62.48 16**

**62.5 18**

**62.52 13**

**62.54 20**

**62.56 14**

**62.58 15**

**62.6 22**

**62.62 20**

**62.64 15**

**62.66 14**

**62.68 14**

**62.7 12**

**62.72 13**

**62.74 21**

**62.76 18**

**62.78 14**

**62.8 16**

**62.82 25**

**62.84 29**

**62.86 23**

**62.88 21**

**62.9 24**

**62.92 23**

**62.94 31**

**62.96 22**

**62.98 30**

**63 38**

**63.02 34**

**63.04 26**

**63.06 21**

**63.08 29**

**63.1 30**

**63.12 22**

**63.14 21**

**63.16 28**

**63.18 27**

**63.2 23**

**63.22 25**

**63.24 23**

**63.26 28**

**63.28 27**

**63.3 21**

**63.32 20**

**63.34 25**

**63.36 24**

**63.38 22**

**63.4 32**

**63.42 23**

**63.44 27**

**63.46 28**

**63.48 17**

**63.5 16**

**63.52 27**

**63.54 22**

**63.56 17**

**63.58 20**

**63.6 19**

**63.62 19**

**63.64 16**

**63.66 13**

**63.68 12**

**63.7 15**

**63.72 16**

**63.74 14**

**63.76 20**

**63.78 14**

**63.8 16**

**63.82 15**

**63.84 21**

**63.86 21**

**63.88 15**

**63.9 13**

**63.92 20**

**63.94 20**

**63.96 16**

**63.98 8**

**64 19**

**64.02 17**

**64.04 20**

**64.06 10**

**64.08 17**

**64.1 18**

**64.12 20**

**64.14 17**

**64.16 20**

**64.18 19**

**64.2 22**

**64.22 19**

**64.24 21**

**64.26 20**

**64.28 19**

**64.3 21**

**64.32 21**

**64.34 24**

**64.36 24**

**64.38 25**

**64.4 20**

**64.42 30**

**64.44 30**

**64.46 32**

**64.48 28**

**64.5 22**

**64.52 30**

**64.54 23**

**64.56 23**

**64.58 25**

**64.6 28**

**64.62 29**

**64.64 29**

**64.66 23**

**64.68 33**

**64.7 27**

**64.72 24**

**64.74 20**

**64.76 32**

**64.78 26**

**64.8 29**

**64.82 25**

**64.84 26**

**64.86 33**

**64.88 26**

**64.9 19**

**64.92 21**

**64.94 21**

**64.96 18**

**64.98 18**

**65 21**

**65.02 20**

**65.04 14**

**65.06 13**

**65.08 12**

**65.1 11**

**65.12 10**

**65.14 12**

**65.16 15**

**65.18 17**

**65.2 13**

**65.22 16**

**65.24 18**

**65.26 16**

**65.28 15**

**65.3 16**

**65.32 12**

**65.34 10**

**65.36 13**

**65.38 19**

**65.4 12**

**65.42 13**

**65.44 8**

**65.46 9**

**65.48 13**

**65.5 9**

**65.52 8**

**65.54 11**

**65.56 13**

**65.58 16**

**65.6 21**

**65.62 13**

**65.64 18**

**65.66 8**

**65.68 13**

**65.7 9**

**65.72 18**

**65.74 20**

**65.76 20**

**65.78 12**

**65.8 12**

**65.82 18**

**65.84 19**

**65.86 21**

**65.88 20**

**65.9 16**

**65.92 17**

**65.94 18**

**65.96 11**

**65.98 10**

**66 17**

**66.02 16**

**66.04 19**

**66.06 16**

**66.08 13**

**66.1 11**

**66.12 12**

**66.14 13**

**66.16 17**

**66.18 13**

**66.2 13**

**66.22 16**

**66.24 11**

**66.26 7**

**66.28 9**

**66.3 10**

**66.32 12**

**66.34 17**

**66.36 17**

**66.38 14**

**66.4 15**

**66.42 14**

**66.44 21**

**66.46 22**

**66.48 17**

**66.5 22**

**66.52 16**

**66.54 20**

**66.56 18**

**66.58 19**

**66.6 18**

**66.62 22**

**66.64 21**

**66.66 18**

**66.68 16**

**66.7 18**

**66.72 20**

**66.74 15**

**66.76 17**

**66.78 22**

**66.8 11**

**66.82 19**

**66.84 26**

**66.86 26**

**66.88 19**

**66.9 15**

**66.92 28**

**66.94 21**

**66.96 26**

**66.98 18**

**67 24**

**67.02 15**

**67.04 15**

**67.06 21**

**67.08 17**

**67.1 19**

**67.12 13**

**67.14 17**

**67.16 22**

**67.18 19**

**67.2 20**

**67.22 21**

**67.24 10**

**67.26 14**

**67.28 25**

**67.3 18**

**67.32 17**

**67.34 19**

**67.36 16**

**67.38 14**

**67.4 19**

**67.42 17**

**67.44 14**

**67.46 10**

**67.48 13**

**67.5 15**

**67.52 17**

**67.54 16**

**67.56 14**

**67.58 21**

**67.6 21**

**67.62 17**

**67.64 11**

**67.66 13**

**67.68 10**

**67.7 6**

**67.72 12**

**67.74 15**

**67.76 13**

**67.78 16**

**67.8 14**

**67.82 19**

**67.84 17**

**67.86 17**

**67.88 19**

**67.9 12**

**67.92 10**

**67.94 14**

**67.96 14**

**67.98 18**

**68 11**

**68.02 13**

**68.04 24**

**68.06 12**

**68.08 13**

**68.1 12**

**68.12 17**

**68.14 14**

**68.16 12**

**68.18 14**

**68.2 15**

**68.22 16**

**68.24 8**

**68.26 9**

**68.28 13**

**68.3 18**

**68.32 15**

**68.34 15**

**68.36 18**

**68.38 15**

**68.4 14**

**68.42 9**

**68.44 14**

**68.46 17**

**68.48 15**

**68.5 7**

**68.52 9**

**68.54 7**

**68.56 7**

**68.58 11**

**68.6 13**

**68.62 6**

**68.64 8**

**68.66 11**

**68.68 7**

**68.7 10**

**68.72 11**

**68.74 16**

**68.76 15**

**68.78 7**

**68.8 8**

**68.82 11**

**68.84 7**

**68.86 10**

**68.88 11**

**68.9 12**

**68.92 10**

**68.94 12**

**68.96 12**

**68.98 14**

**69 13**

**69.02 11**

**69.04 14**

**69.06 9**

**69.08 10**

**69.1 17**

**69.12 13**

**69.14 15**

**69.16 18**

**69.18 15**

**69.2 17**

**69.22 13**

**69.24 9**

**69.26 18**

**69.28 21**

**69.3 10**

**69.32 13**

**69.34 13**

**69.36 16**

**69.38 15**

**69.4 12**

**69.42 13**

**69.44 13**

**69.46 9**

**69.48 12**

**69.5 11**

**69.52 10**

**69.54 12**

**69.56 9**

**69.58 10**

**69.6 11**

**69.62 13**

**69.64 10**

**69.66 10**

**69.68 16**

**69.7 8**

**69.72 12**

**69.74 14**

**69.76 12**

**69.78 11**

**69.8 9**

**69.82 10**

**69.84 15**

**69.86 10**

**69.88 21**

**69.9 15**

**69.92 8**

**69.94 14**

**69.96 11**

**69.98 13**

**70 8**

**70.02 14**

**70.04 17**

**70.06 16**

**70.08 10**

**70.1 10**

**70.12 4**

**70.14 8**

**70.16 9**

**70.18 12**

**70.2 10**

**70.22 13**

**70.24 15**

**70.26 11**

**70.28 9**

**70.3 9**

**70.32 10**

**70.34 8**

**70.36 4**

**70.38 7**

**70.4 7**

**70.42 9**

**70.44 7**

**70.46 10**

**70.48 6**

**70.5 10**

**70.52 8**

**70.54 6**

**70.56 9**

**70.58 5**

**70.6 9**

**70.62 6**

**70.64 9**

**70.66 11**

**70.68 8**

**70.7 9**

**70.72 9**

**70.74 17**

**70.76 14**

**70.78 9**

**70.8 7**

**70.82 10**

**70.84 9**

**70.86 8**

**70.88 7**

**70.9 7**

**70.92 9**

**70.94 6**

**70.96 6**

**70.98 9**

**71 5**

**71.02 10**

**71.04 9**

**71.06 9**

**71.08 7**

**71.1 11**

**71.12 11**

**71.14 12**

**71.16 9**

**71.18 11**

**71.2 15**

**71.22 11**

**71.24 12**

**71.26 7**

**71.28 10**

**71.3 10**

**71.32 7**

**71.34 14**

**71.36 9**

**71.38 11**

**71.4 12**

**71.42 8**

**71.44 11**

**71.46 11**

**71.48 10**

**71.5 11**

**71.52 10**

**71.54 13**

**71.56 8**

**71.58 11**

**71.6 12**

**71.62 10**

**71.64 4**

**71.66 11**

**71.68 7**

**71.7 11**

**71.72 17**

**71.74 10**

**71.76 12**

**71.78 8**

**71.8 8**

**71.82 11**

**71.84 11**

**71.86 14**

**71.88 10**

**71.9 11**

**71.92 10**

**71.94 12**

**71.96 11**

**71.98 9**

**72 13**

**72.02 11**

**72.04 13**

**72.06 9**

**72.08 9**

**72.1 9**

**72.12 9**

**72.14 8**

**72.16 10**

**72.18 6**

**72.2 9**

**72.22 9**

**72.24 7**

**72.26 7**

**72.28 13**

**72.3 10**

**72.32 7**

**72.34 14**

**72.36 9**

**72.38 8**

**72.4 11**

**72.42 16**

**72.44 8**

**72.46 13**

**72.48 9**

**72.5 7**

**72.52 17**

**72.54 10**

**72.56 7**

**72.58 6**

**72.6 6**

**72.62 17**

**72.64 5**

**72.66 10**

**72.68 8**

**72.7 10**

**72.72 11**

**72.74 9**

**72.76 12**

**72.78 13**

**72.8 9**

**72.82 13**

**72.84 11**

**72.86 8**

**72.88 6**

**72.9 13**

**72.92 10**

**72.94 7**

**72.96 6**

**72.98 9**

**73 12**

**73.02 10**

**73.04 8**

**73.06 11**

**73.08 11**

**73.1 10**

**73.12 10**

**73.14 8**

**73.16 8**

**73.18 14**

**73.2 12**

**73.22 10**

**73.24 13**

**73.26 8**

**73.28 16**

**73.3 12**

**73.32 8**

**73.34 10**

**73.36 13**

**73.38 13**

**73.4 13**

**73.42 10**

**73.44 11**

**73.46 15**

**73.48 13**

**73.5 18**

**73.52 14**

**73.54 17**

**73.56 20**

**73.58 13**

**73.6 17**

**73.62 20**

**73.64 27**

**73.66 16**

**73.68 17**

**73.7 15**

**73.72 14**

**73.74 17**

**73.76 12**

**73.78 20**

**73.8 16**

**73.82 18**

**73.84 23**

**73.86 16**

**73.88 23**

**73.9 14**

**73.92 15**

**73.94 13**

**73.96 10**

**73.98 10**

**74 15**

**74.02 15**

**74.04 9**

**74.06 22**

**74.08 18**

**74.1 12**

**74.12 19**

**74.14 17**

**74.16 16**

**74.18 18**

**74.2 17**

**74.22 21**

**74.24 12**

**74.26 21**

**74.28 14**

**74.3 13**

**74.32 15**

**74.34 18**

**74.36 17**

**74.38 17**

**74.4 20**

**74.42 11**

**74.44 20**

**74.46 20**

**74.48 16**

**74.5 16**

**74.52 22**

**74.54 25**

**74.56 15**

**74.58 25**

**74.6 21**

**74.62 22**

**74.64 15**

**74.66 15**

**74.68 21**

**74.7 11**

**74.72 13**

**74.74 14**

**74.76 15**

**74.78 15**

**74.8 15**

**74.82 16**

**74.84 14**

**74.86 24**

**74.88 18**

**74.9 10**

**74.92 16**

**74.94 13**

**74.96 17**

**74.98 10**

**75 15**

**75.02 13**

**75.04 9**

**75.06 12**

**75.08 12**

**75.1 17**

**75.12 12**

**75.14 19**

**75.16 11**

**75.18 16**

**75.2 15**

**75.22 11**

**75.24 10**

**75.26 18**

**75.28 13**

**75.3 15**

**75.32 21**

**75.34 16**

**75.36 11**

**75.38 11**

**75.4 4**

**75.42 22**

**75.44 17**

**75.46 15**

**75.48 17**

**75.5 13**

**75.52 17**

**75.54 18**

**75.56 11**

**75.58 14**

**75.6 7**

**75.62 17**

**75.64 15**

**75.66 15**

**75.68 14**

**75.7 18**

**75.72 16**

**75.74 9**

**75.76 14**

**75.78 20**

**75.8 15**

**75.82 15**

**75.84 17**

**75.86 11**

**75.88 13**

**75.9 23**

**75.92 13**

**75.94 17**

**75.96 11**

**75.98 16**

**76 14**

**76.02 13**

**76.04 15**

**76.06 13**

**76.08 18**

**76.1 13**

**76.12 18**

**76.14 18**

**76.16 14**

**76.18 10**

**76.2 12**

**76.22 12**

**76.24 15**

**76.26 13**

**76.28 14**

**76.3 17**

**76.32 16**

**76.34 16**

**76.36 22**

**76.38 11**

**76.4 13**

**76.42 17**

**76.44 13**

**76.46 12**

**76.48 17**

**76.5 13**

**76.52 11**

**76.54 18**

**76.56 20**

**76.58 13**

**76.6 14**

**76.62 9**

**76.64 16**

**76.66 14**

**76.68 13**

**76.7 19**

**76.72 15**

**76.74 10**

**76.76 18**

**76.78 19**

**76.8 18**

**76.82 13**

**76.84 15**

**76.86 14**

**76.88 21**

**76.9 11**

**76.92 15**

**76.94 17**

**76.96 12**

**76.98 11**

**77 15**

**77.02 20**

**77.04 12**

**77.06 16**

**77.08 22**

**77.1 17**

**77.12 10**

**77.14 17**

**77.16 18**

**77.18 16**

**77.2 14**

**77.22 10**

**77.24 16**

**77.26 13**

**77.28 15**

**77.3 14**

**77.32 20**

**77.34 10**

**77.36 15**

**77.38 19**

**77.4 15**

**77.42 10**

**77.44 11**

**77.46 14**

**77.48 21**

**77.5 19**

**77.52 9**

**77.54 16**

**77.56 12**

**77.58 19**

**77.6 17**

**77.62 12**

**77.64 12**

**77.66 17**

**77.68 17**

**77.7 13**

**77.72 9**

**77.74 17**

**77.76 12**

**77.78 13**

**77.8 10**

**77.82 13**

**77.84 9**

**77.86 9**

**77.88 17**

**77.9 15**

**77.92 8**

**77.94 17**

**77.96 15**

**77.98 11**

**78 12**

**78.02 9**

**78.04 4**

**78.06 15**

**78.08 9**

**78.1 12**

**78.12 11**

**78.14 7**

**78.16 11**

**78.18 12**

**78.2 10**

**78.22 12**

**78.24 11**

**78.26 11**

**78.28 6**

**78.3 13**

**78.32 11**

**78.34 11**

**78.36 8**

**78.38 7**

**78.4 10**

**78.42 9**

**78.44 7**

**78.46 5**

**78.48 14**

**78.5 11**

**78.52 13**

**78.54 12**

**78.56 13**

**78.58 13**

**78.6 15**

**78.62 12**

**78.64 16**

**78.66 9**

**78.68 11**

**78.7 12**

**78.72 9**

**78.74 9**

**78.76 7**

**78.78 13**

**78.8 11**

**78.82 12**

**78.84 14**

**78.86 8**

**78.88 10**

**78.9 11**

**78.92 13**

**78.94 12**

**78.96 11**

**78.98 4**

**79 9**

**79.02 15**

**79.04 11**

**79.06 13**

**79.08 18**

**79.1 15**

**79.12 12**

**79.14 15**

**79.16 8**

**79.18 9**

**79.2 8**

**79.22 13**

**79.24 11**

**79.26 17**

**79.28 13**

**79.3 16**

**79.32 12**

**79.34 10**

**79.36 15**

**79.38 13**

**79.4 14**

**79.42 12**

**79.44 17**

**79.46 16**

**79.48 11**

**79.5 6**

**79.52 10**

**79.54 9**

**79.56 11**

**79.58 9**

**79.6 11**

**79.62 13**

**79.64 14**

**79.66 9**

**79.68 11**

**79.7 14**

**79.72 11**

**79.74 12**

**79.76 10**

**79.78 8**

**79.8 7**

**79.82 6**

**79.84 11**

**79.86 6**

**79.88 13**

**79.9 9**

**79.92 9**

**79.94 6**

**79.96 10**

**79.98 7**

**80 8**
