# Supplementary figures and images for: Decoration of Ag nanoparticles on CoMoO4 rods for efficient electrochemical reduction of CO2
Source: Sci Rep. 2024 Jan 16;14:1406. doi: 10.1038/s41598-024-51680-w (PMC10792071; doi:10.1038/s41598-024-51680-w)

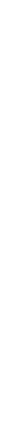

Supplement: Supplementary file 1 — Supplementary Information 1. [file 41598_2024_51680_MOESM1_ESM.zip › raw data for scientific reports/FEs/CoMoO4 FEs .docx]
