# Supplementary material for: Decoration of Ag nanoparticles on CoMoO4 rods for efficient electrochemical reduction of CO2
Source: Sci Rep. 2024 Jan 16;14:1406. doi: 10.1038/s41598-024-51680-w (PMC10792071; doi:10.1038/s41598-024-51680-w)
Supplement: Supplementary file 2 — Supplementary Information 2. [file 41598_2024_51680_MOESM2_ESM.docx]

**Supporting Information**

**Decoration of Ag nanoparticles on CoMoO_4_ rods for efficient electrochemical reduction of CO_2_**

Schindra Kumar Ray^*^, Rabin Dahal, Moses D. Ashie, and Bishnu Prasad Bastakoti^*^

*Department of Chemistry, North Carolina A & T State University, 1601 E Market St, Greensboro, NC 27411, USA* *﻿*

Corresponding authors: skray@ncat.edu and [bpbastakoti@ncat.edu](mailto:bpbastakoti@ncat.edu)


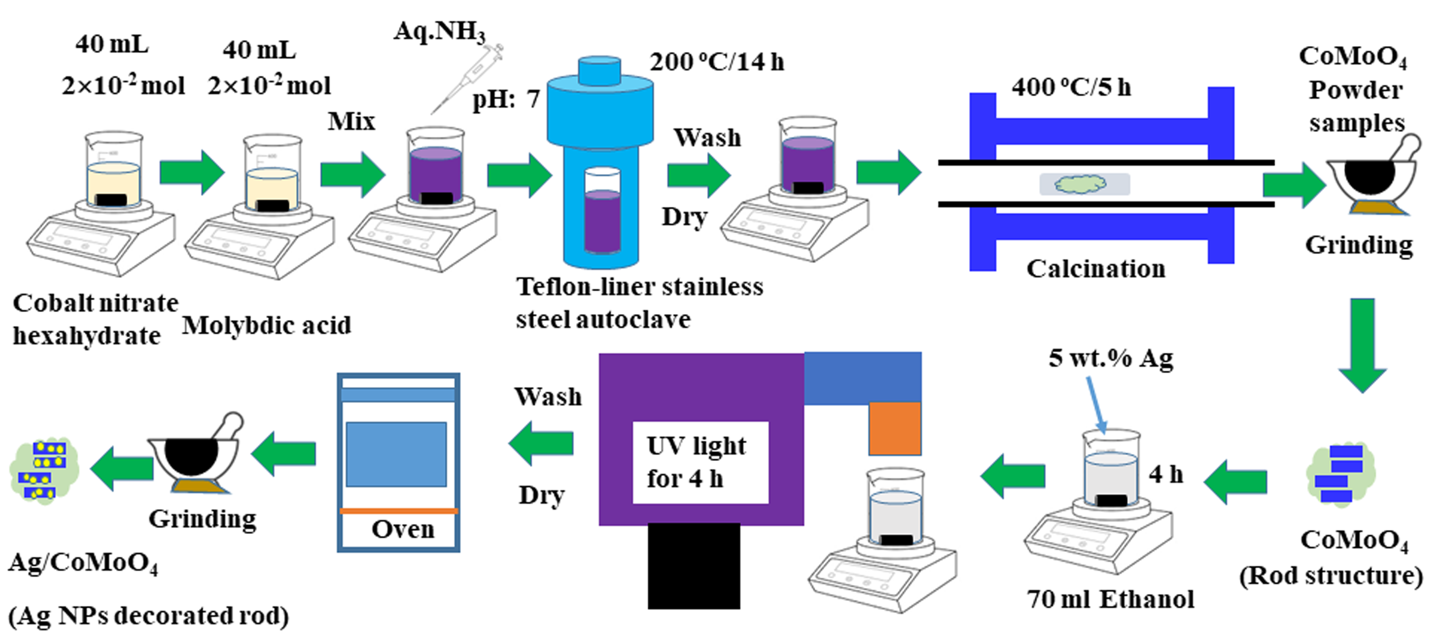


**Fig. S1.** Schematic illustration of Ag/CoMoO_4_ synthesis by hydrothermal and photodeposition techniques.

**ICP-OES analysis Ag/CoMoO_4_**

2.96 mg of solid sample was weighed employing high-precision analytical balances. For acid digestion, 10 ml of concentrated nitric acid (67-70%, Fisher Scientific) and 0.5 ml of hydrofluoric acid (48-51%, VWR Chemicals) were added to the dedicated digestion tubes. These acids serve as reagents for sample digestion, allowing for subsequent elemental analysis. In pre-digestion, acid-treated samples were allowed to stand for 10 minutes. This phase initiates the breakdown of the alloy, priming them for the subsequent digestion process.

According to automated microwave Digestion process, the digestion tubes, post pre-digestion, were subjected to an advanced and automated sequential microwave digester, the MARS 5, manufactured by CEM Microwave Technology Ltd. (North Carolina, USA). Controlled microwave energy was administered to the samples within the digester, ensuring uniform and efficient sample digestion. The digestion parameters (power: 800 W, temperature: 220 °C, ramp time: 30 min and hold time: 15 min) were adjusted. The result of the digestion process yielded a pristine and transparent aqueous solution. This solution contains the solubilized components of the original samples, devoid of solid residues. For preparation for ICP-OES analysis, it was diluted with double deionized (DI) water until a final volume of 50 mL was attained. To perform the leaching of Ag NPs in solution, acid digestion was not performed. The parameters (RF power: 1500 W, nebulizer: GemCone Low flow, nebulizer gas flow rate: 0.85 L/min, plasma gas flow rate-argon: 10 L/min, auxiliary gas flow rate: 0.35 L/min, and sample flow rate: 1 mL/min) were adjusted for ICP-OES elemental analysis of samples.

For quantitative analysis, commercially available standards for Mo, Ag, and Co were acquired from High Purity Standards (Charleston, South Carolina) to ensure accuracy and precision in the analytical process. The following emission lines were used for quantitative analysis for the identification of wt.% of Co, Mo, and Ag.

| Element | State | Emission line (nm) | Wt.% |
| --- | --- | --- | --- |
| Mo | II | 202.031 | 40.48 |
| Ag | I | 328.068 | 23.43 |
| Co | II | 228.616 | 2.16 |


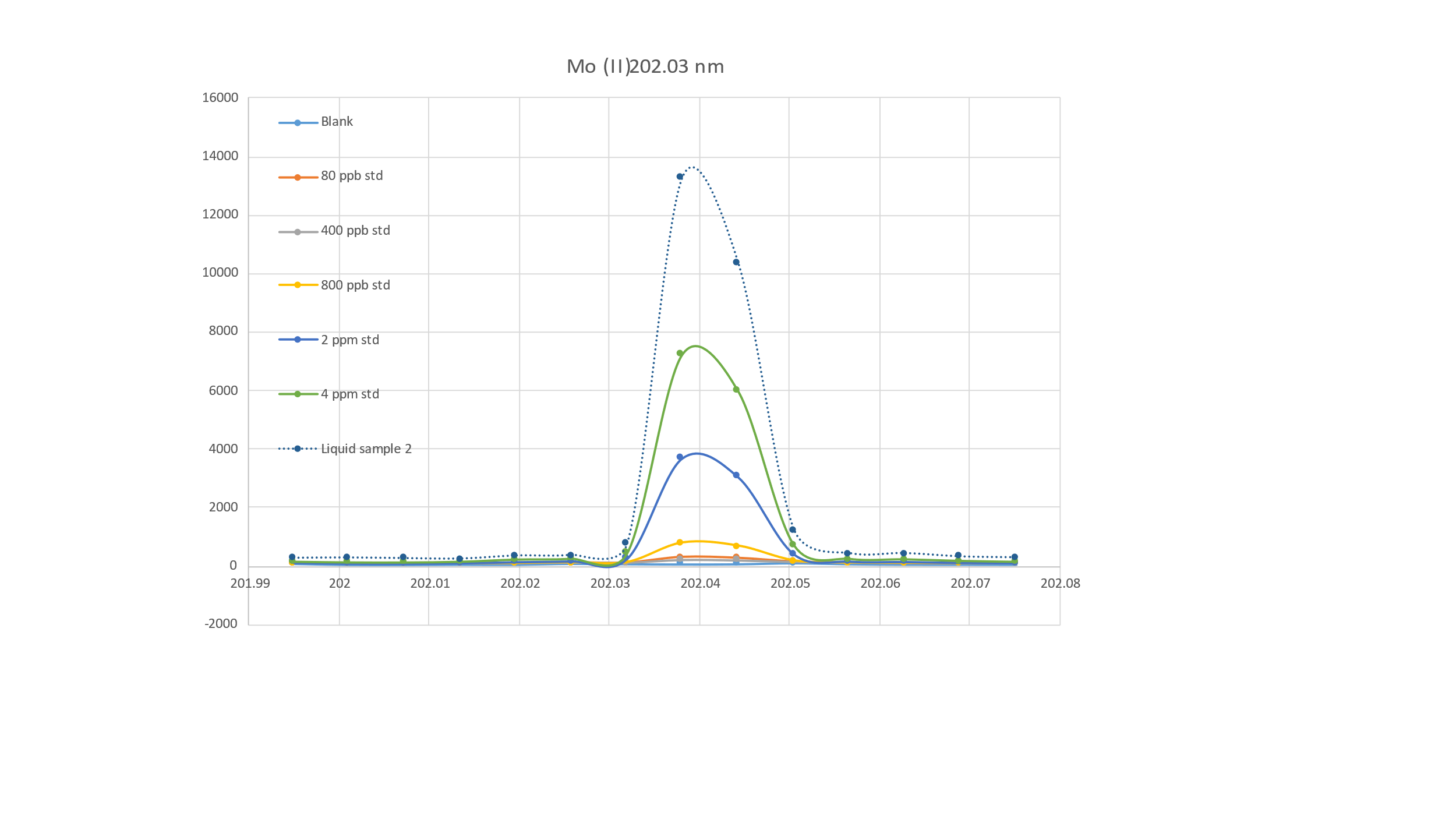


**Fig. S2.** Emission line of Mo.


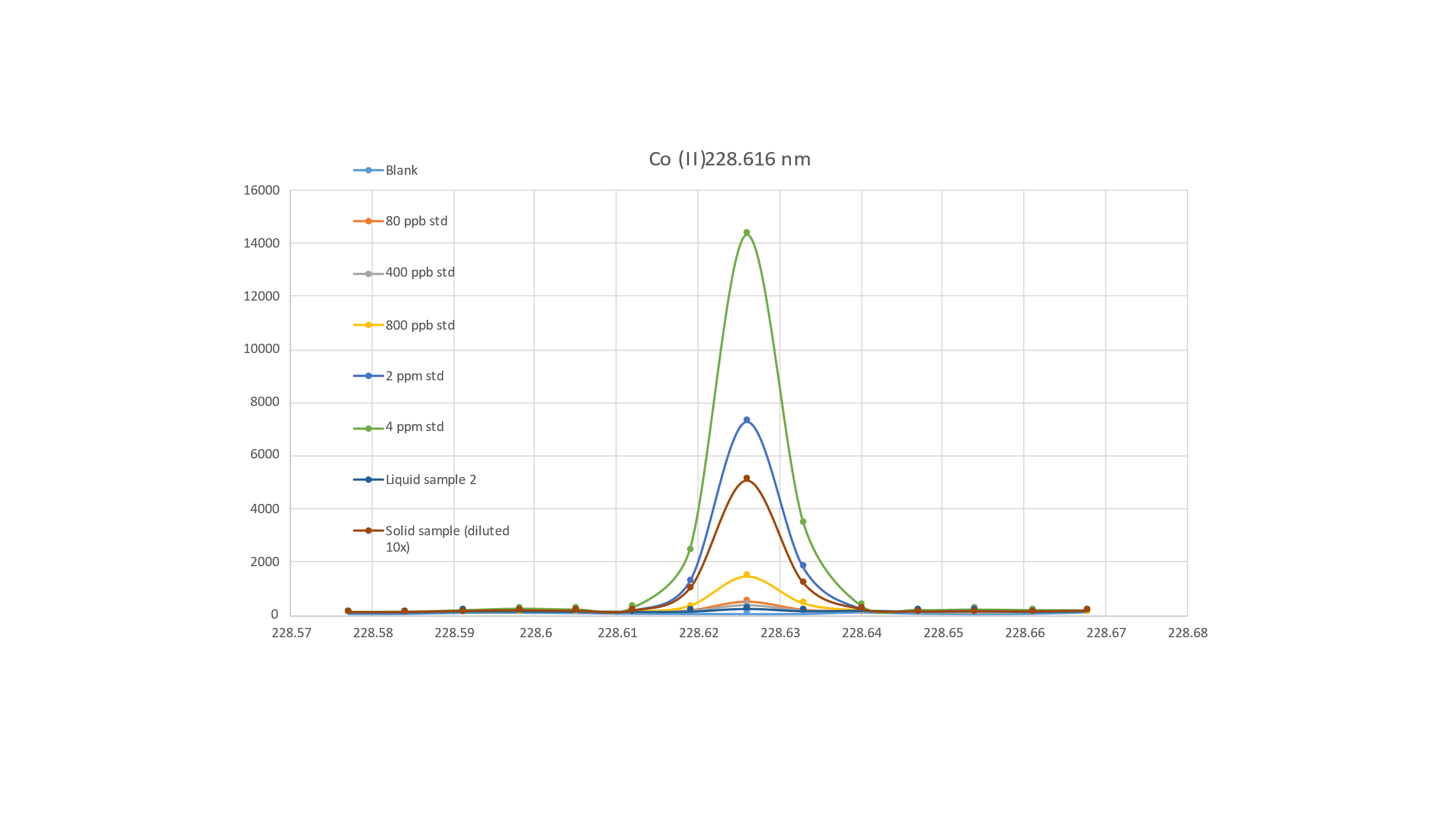


**Fig. S3.** Emission line of Co.


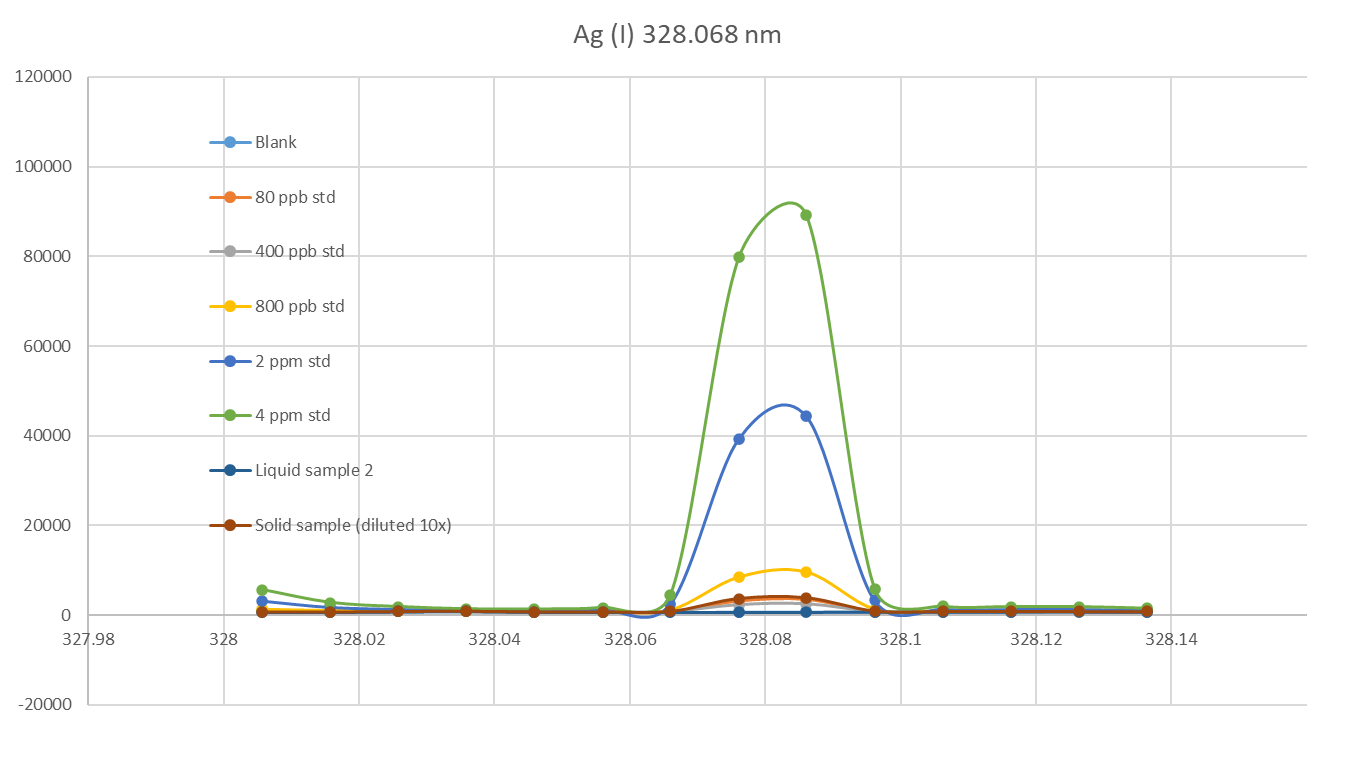


**Fig. S4.** Emission line of Ag.


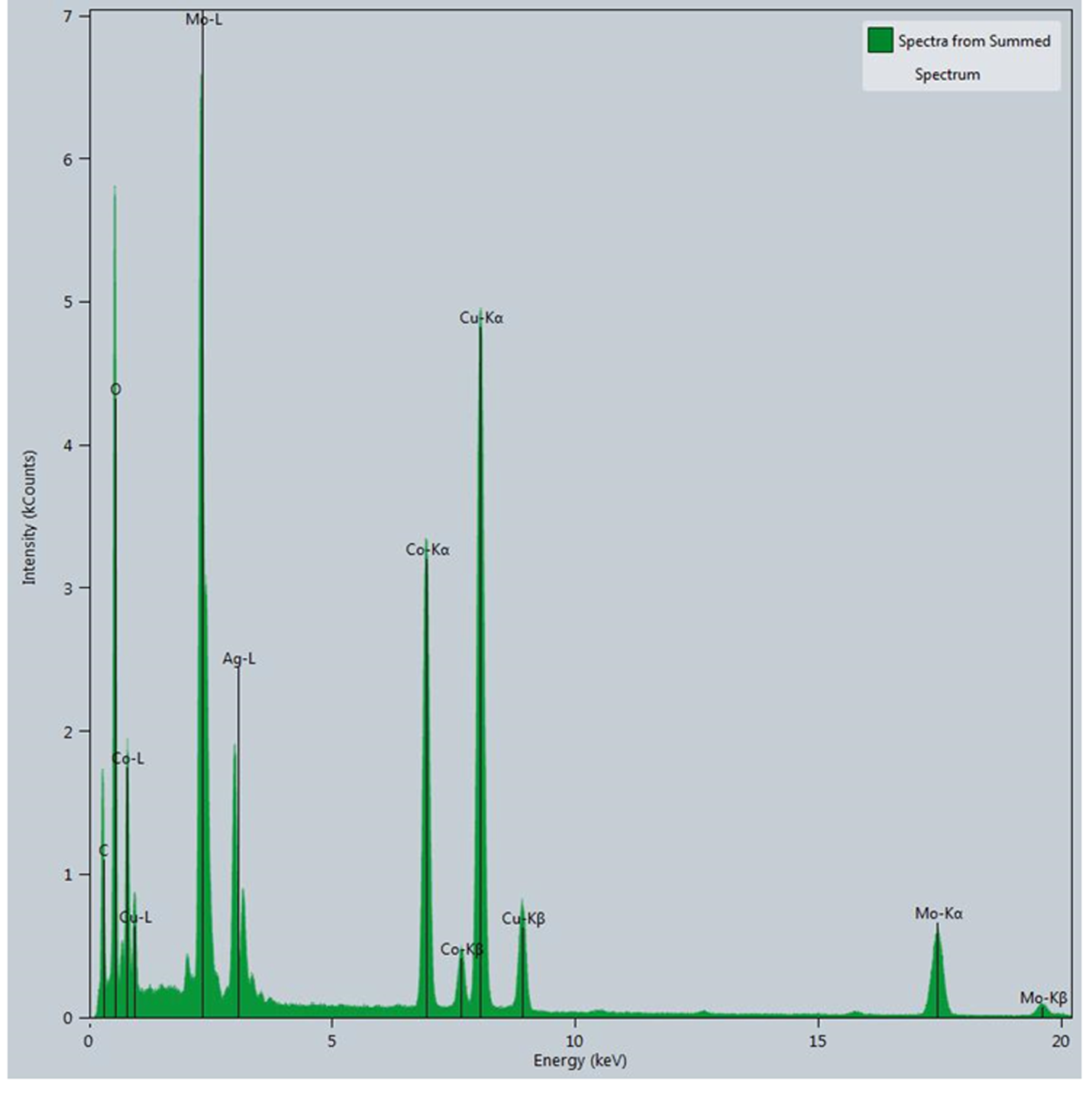


**Fig. S5.** EDS spectrum of Ag/CoMoO_4_

**Table S1**. EDS analysis of Ag/CoMoO_4_.

| Element | Family | Net intensity | Net background | K-factor | Absorption correction | Atomic fraction | Mass fraction |
| --- | --- | --- | --- | --- | --- | --- | --- |
| - | - | counts | counts | - | - | % | % |
| C | K | 1.67E+04 | 2.95E+03 | 1.00E+00 | 1.00E+00 | 1.23E+01 | 2.86E+00 |
| O | K | 6.89E+04 | 4.35E+03 | 7.01E-01 | 1.00E+00 | 2.68E+01 | 8.31E+00 |
| Co | K | 1.11E+05 | 1.08E+04 | 8.77E-01 | 1.00E+00 | 1.46E+01 | 1.67E+01 |
| Cu | K | 1.77E+05 | 1.10E+04 | 9.75E-01 | 1.00E+00 | 2.41E+01 | 2.97E+01 |
| Mo | K | 3.57E+04 | 3.39E+03 | 5.15E+00 | 1.00E+00 | 1.70E+01 | 3.16E+01 |
| Ag | L | 6.44E+04 | 3.36E+04 | 9.49E-01 | 1.00E+00 | 5.17E+00 | 1.08E+01 |


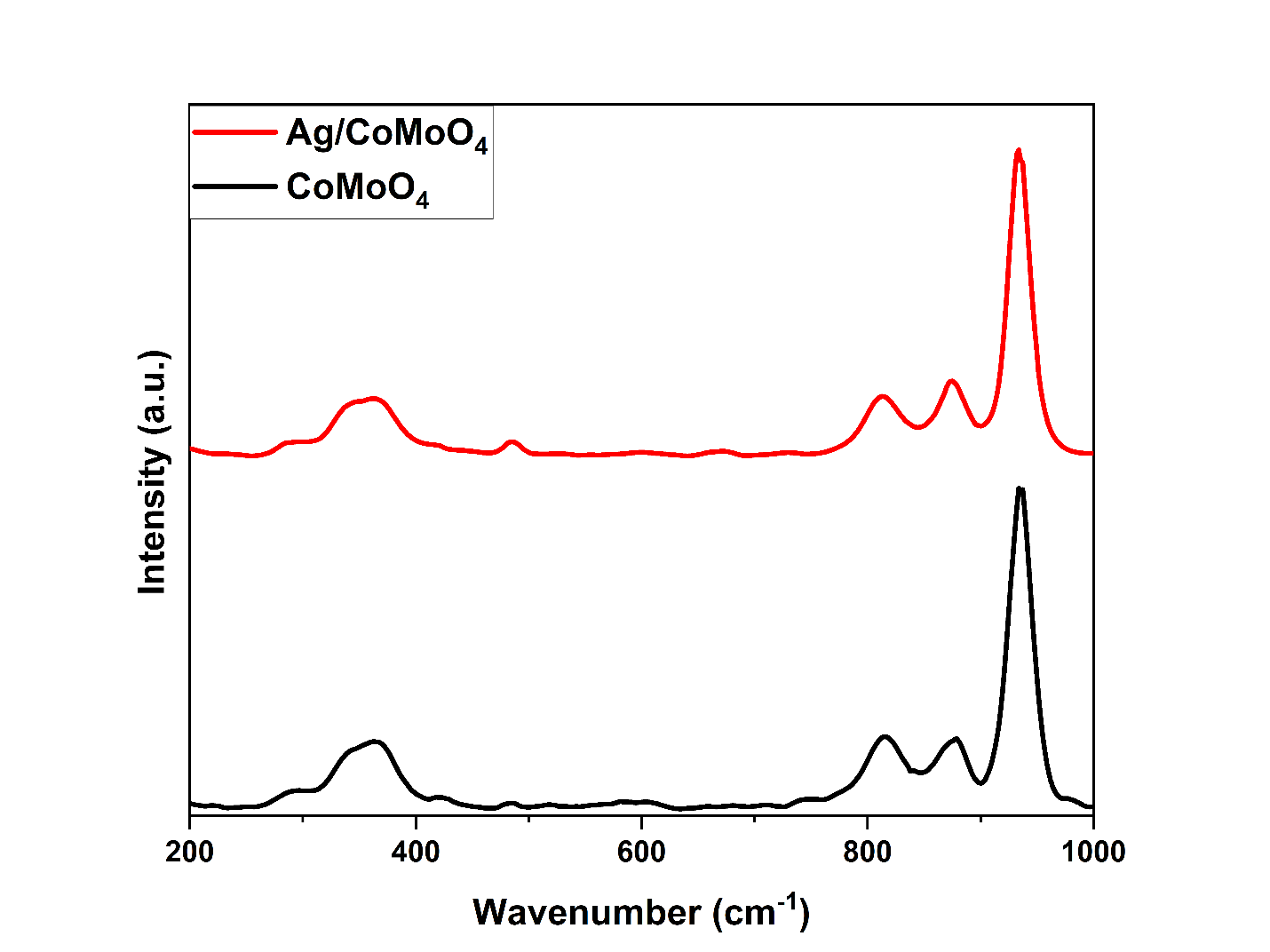


**Fig. S6.** Raman spectra of CoMoO_4_ and Ag/CoMoO_4_.


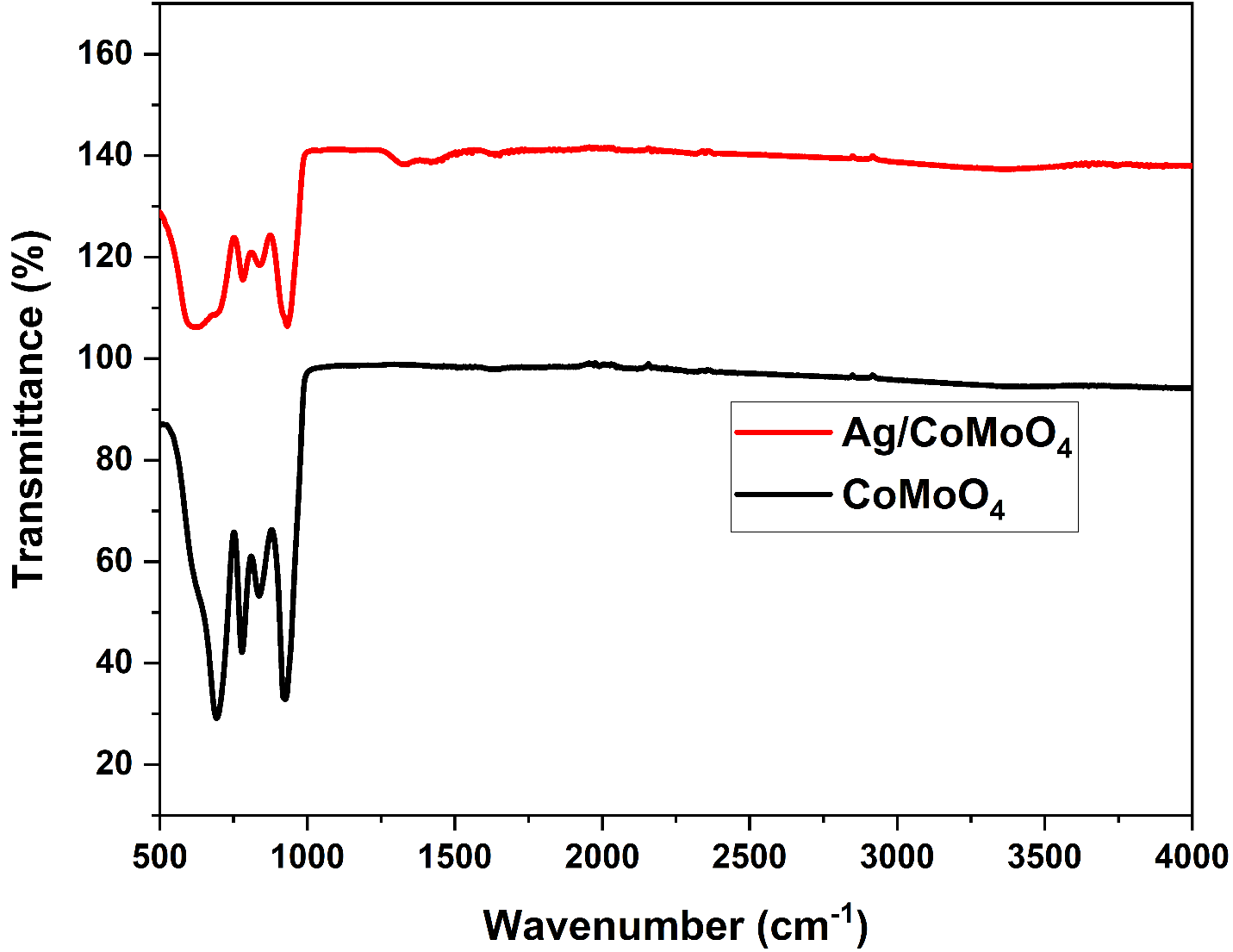


**Fig. S7.** FTIR of samples.


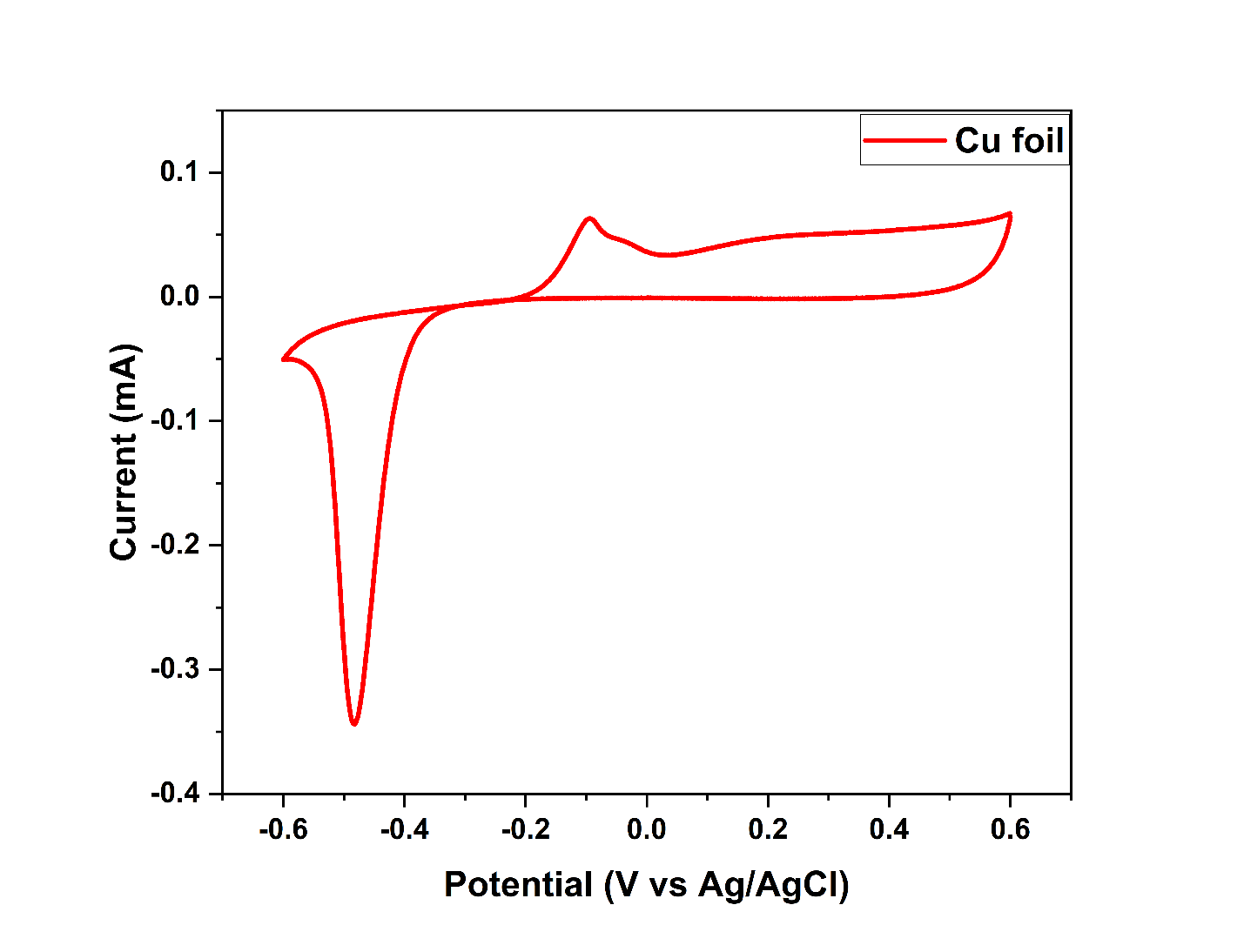


**Fig. S8.** CV curve of Cu-foil at 100 mV/s scan rate.

**
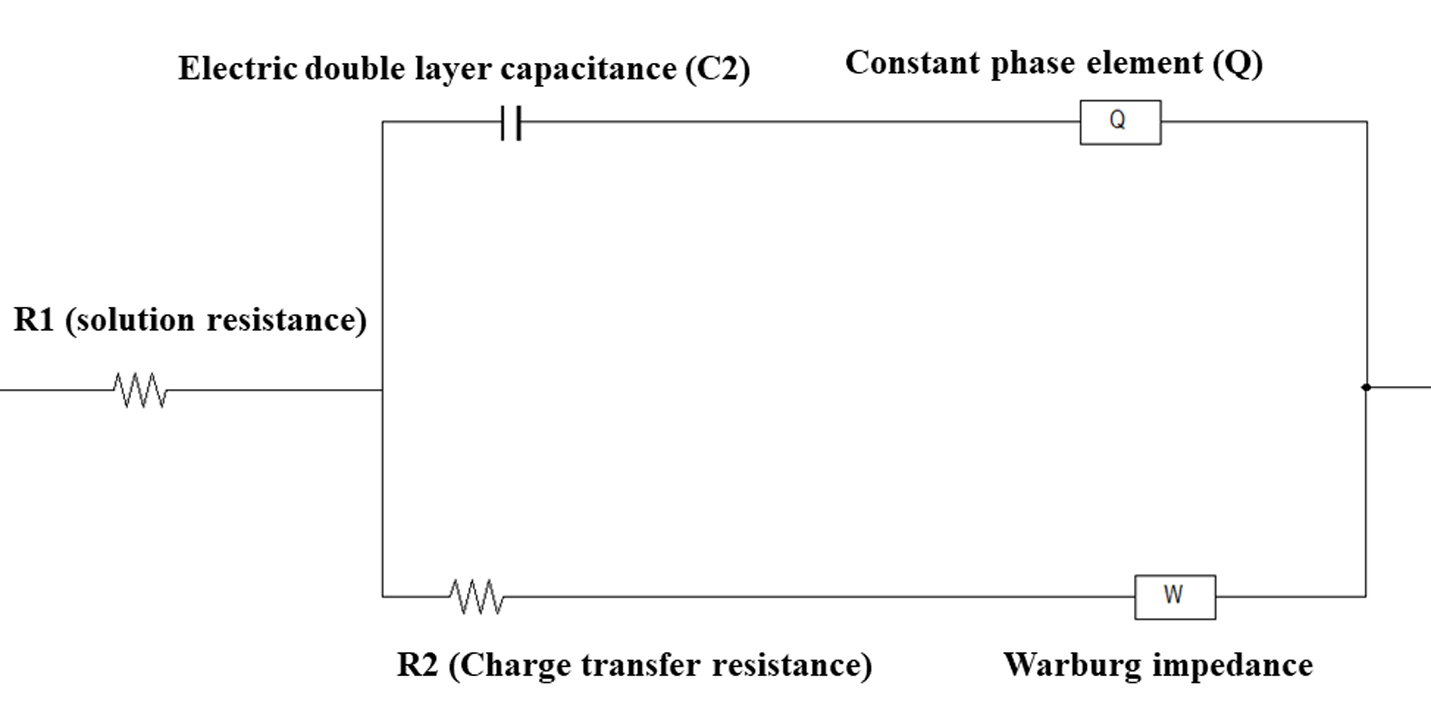
**

**Fig. S9.** Equivalent circuit of CoMoO_4_ and Ag/CoMoO_4_.

**Table S2**. Equivalent series resistance values of CoMoO_4_ and Ag/CoMoO_4_ catalysts obtained from the EIS analysis.

| Samples | R1 (Solution resistance) Ω | Warburg impedance coefficient (σ) | R2 (Charge transfer resistance) Ω | Q (Constant phase element) | Electric double layer Capacitance (F) |
| --- | --- | --- | --- | --- | --- |
| CoMoO4 | 49.68 | 0.000845 | 309.5 | 0.0004296 | 0.0002907 |
| Ag/CoMoO4 | 19.06 | 0.0001 | 41.57 | 0.0004061 | 0.000017965 |

**
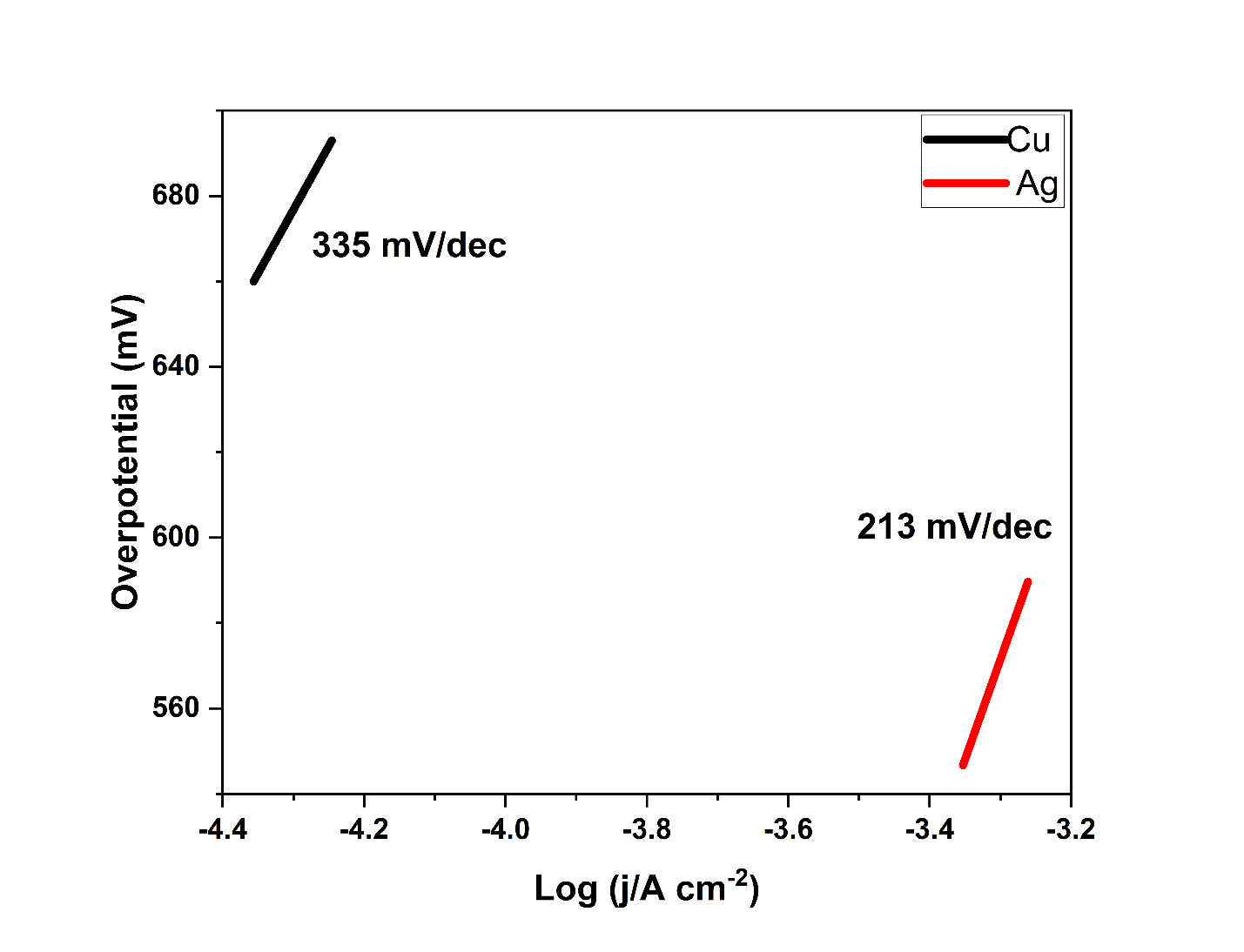
**

**Fig. S10.** Tafel plot of Ag and Cu.

**(a)**

**
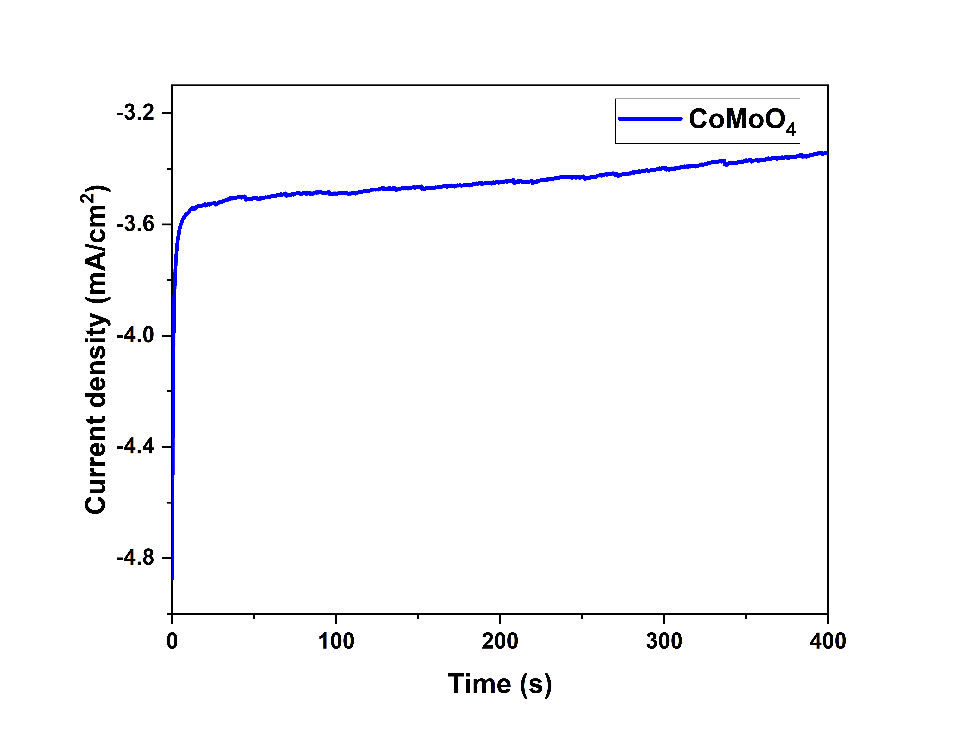
**

**(b)**

**
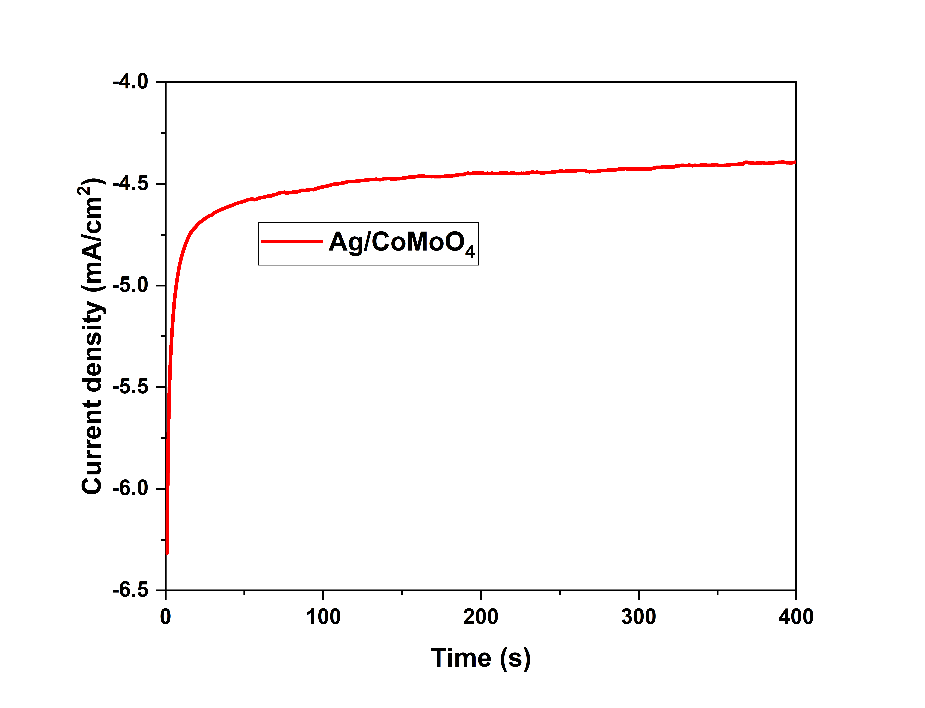
**

**Fig. S11.** Chronoamperometries curves of samples at -1.3 V (V vs RHE) of samples. (a) CoMoO_4_ and (b) Ag/CoMoO_4_.


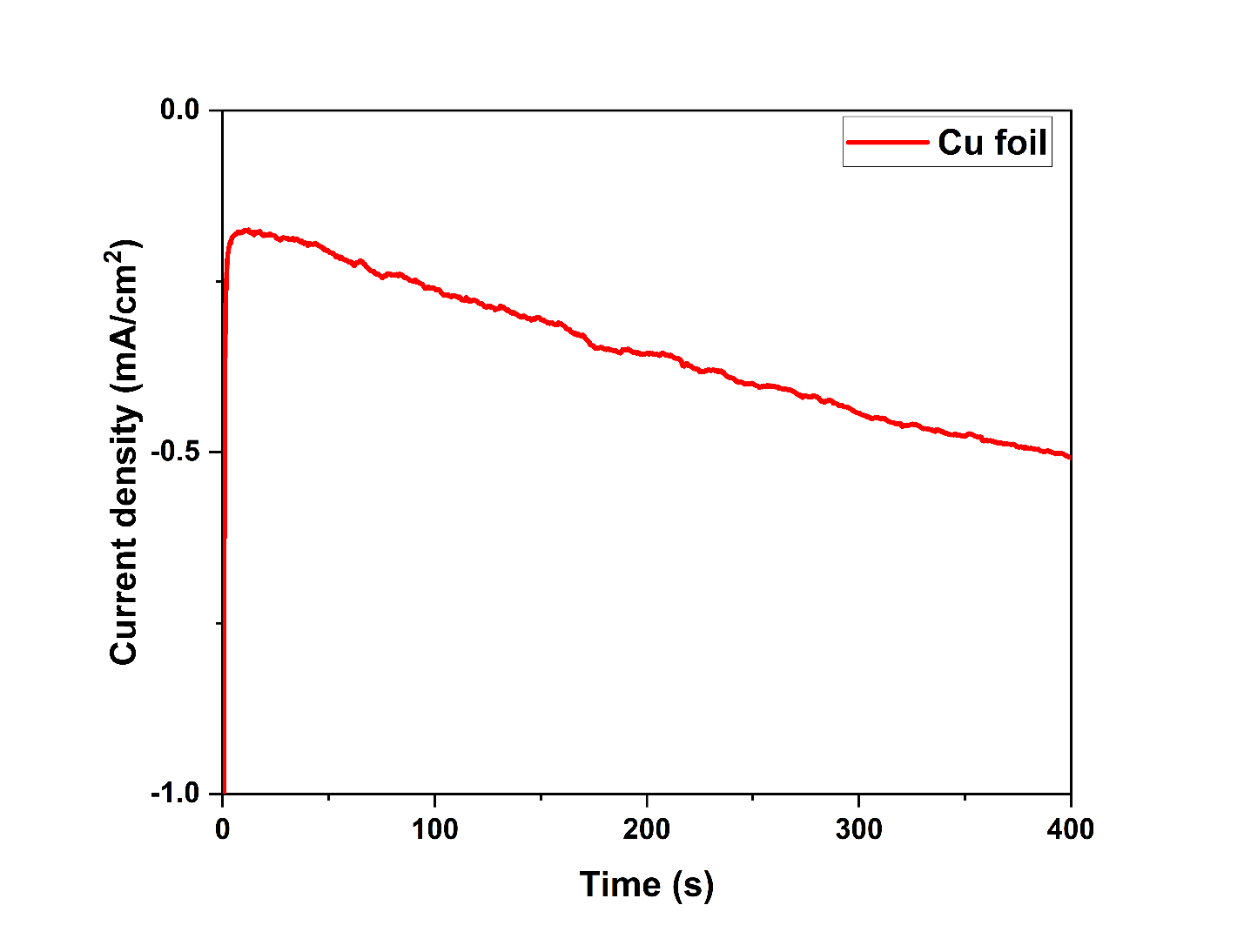


**Fig. S12.** Chronoamperometry curves of Cu-foil at -1.3 V (V vs RHE).


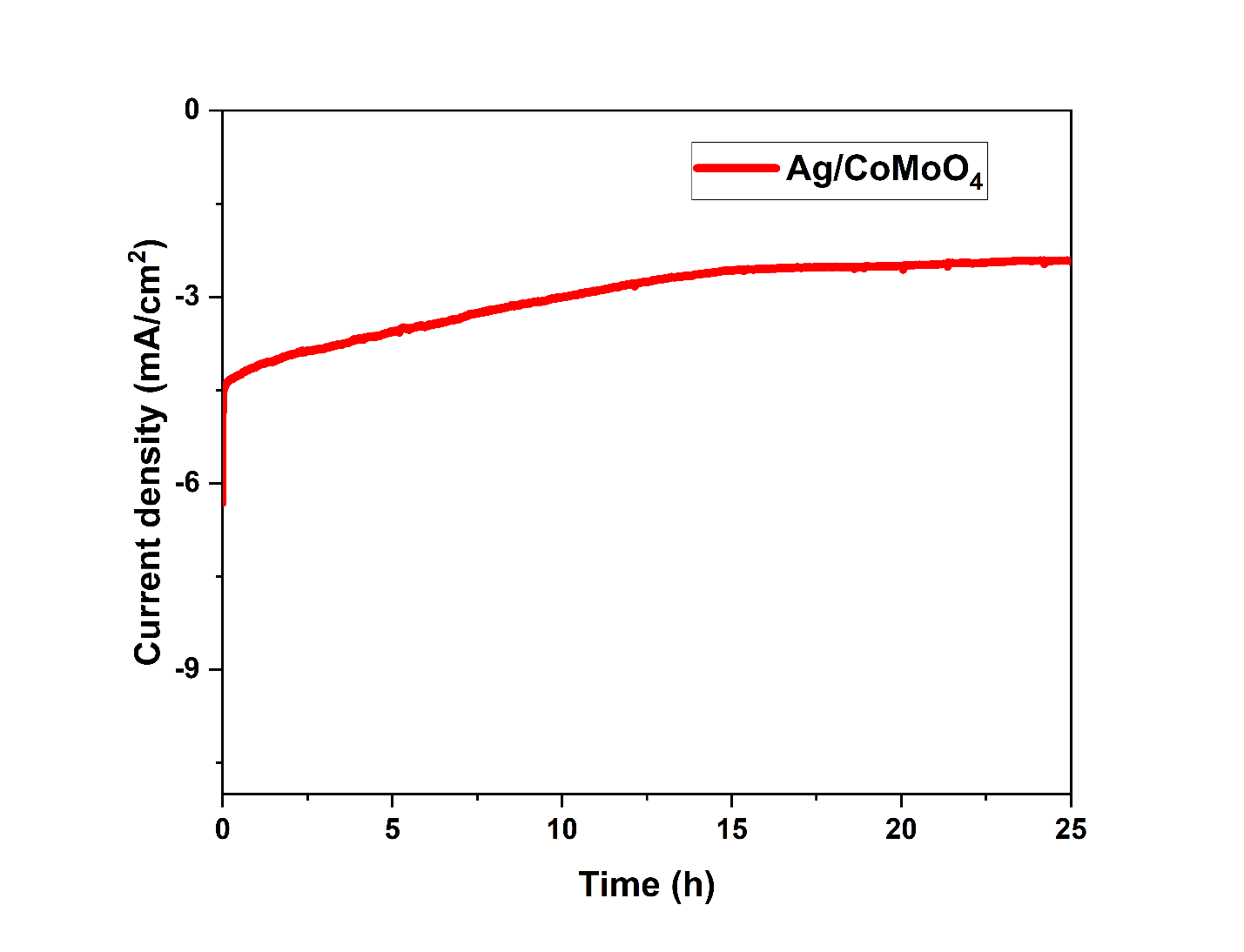


**Fig. S13.** Chronoamperometry stability curve of Ag/CoMoO_4_ at -1.3 V (V vs RHE).

**Table S3.** Data obtained from GC during electrochemical reduction of CO_2_ at -1.3 V (V vs RHE) using various samples.

| CoMoO_4_ | | | | |
| --- | --- | --- | --- | --- |
| Time of sample injection (s) | Current (mA) | Gaseous products (ppm) with Retention time (min) | | |
| 400 | 3.359 | CO | CH_4_ | C_2_H_6_ |
|  |  | 3709.38 (4.45) | 323.93  (1.42) |  |
| Ag/CoMoO_4_ |  |  |  |  |
| 400 | 4.62 | 3032.85  (4.45) | 246.25  (1.43) | 543.25  (7.596) |

**Calculation of FEs of CoMoO_4_ and Ag/CoMoO_4_ for different gaseous products**

**FEs of CoMoO_4_**

1. **Conversion of ppm into mol**

Amount of Co product (mol) = $\frac{3709.38\times0.000001\times101325}{1000000\times8.314\times293}$

Recorded current = 3.359 mA

Flow rate of CO2 = 5 sccm

Volume of sampling loop (V) = 1 cm^3^

Number of moles of electrons required for reducing CO_2_ to CO (*e_output_*) $= \frac{\boldsymbol{2\times3709.38\times0.000001\times101325}}{\boldsymbol{1000000\times8.314\times293}}$

Time required to fill the 1 cm^3^ sampling loop: $\frac{1\times60}{5}$

The total number of moles of electron measured during the sampling period (*e_input_*)

*e_input =_* $\frac{0.003359\times12}{96485}$

FEs for CO = *e_output_*/*e_input_*  ×100

= 56.80 %

In addition, FEs of others were calculated by using same calculation technique.

The number of electrons required to produce a form a molecule of CO, CH_4_, and C_2_H_6_ are 2, 8, and 14 electrons, respectively.

FEs for CH_4_ = 19.80 %

**FEs of Ag/CoMoO_4_**

FEs for CO = 35.30 %

FEs for CH_4_ = 11.40 %

FEs for C_2_H_6_ = 44.20 %
